# The Hidden Value of Adult Informal Care in Europe

**DOI:** 10.1002/hec.4928

**Published:** 2025-01-29

**Authors:** Joan Costa‐Font, Cristina Vilaplana‐Prieto

**Affiliations:** ^1^ London School of Economics and Political Science CESIfo & IZA Bonn London UK; ^2^ University of Murcia Murcia Spain

**Keywords:** caregiving, compensating surplus, daughters, informal care, life satisfaction, the value of time, wellbeing method

## Abstract

The hidden value of adult informal care (IC) refers to the unaccounted value of informal care in overall costs of long‐term care (LTC) estimates. This paper estimates the net value of adult IC in Europe, drawing on a well‐being‐based methodology. We use an instrumental variable strategy and a longitudinal and cross‐country dataset to estimate the causal effect of the extensive and intensive margin of caregiving on subjective well‐being. We estimate the so‐called compensating surplus (CS), namely the income equivalent transfer, to compensate for the net disutility of caregiving. We show that IC reduces average subjective well‐being by about 1% compared to the mean (6% among co‐residential caregivers). Relative to a country's Gross Domestic Product (GDP), the value of IC ranges between 4.2% in France and 0.85% in Germany. Such relative value declines as the country's share of formal LTC spending increases. These results call for a reconsideration of the existing classifications of LTC regimes. We estimate that the average CS per hour for IC is 9.55€, with a range from 22€ per hour in Switzerland to 5€ per hour in Spain. Additionally, we estimate that the long‐term CS (estimated using an individual’s permanent income) tends to be lower than short‐term CS (estimated using an individual’s current income).

## 1 Introduction

In most European countries, informal care (IC), namely non‐professional care aimed at supporting individuals with their basic and instrumental activities of daily living, is the most common type of support provided to older adults (Rocard and Llena‐Nozal 2022). However, while informal carers may receive some government and social support—such as training, financial assistance, and respite services—they generally do not enjoy the same level of social protection as those in formal care employment (Triantafillou et al. 2010). Hence, the social value of the IC provided by them is typically “hidden” from the financial long‐term care cost estimates.

This paper examines such hidden value of IC in Europe, both in terms of its total and relative value, as well as the value per hour of care supplied by caregivers. Given that the value of IC is hidden, a financial perspective to measuring the costs of informal care will result in an estimate that is both largely and systematically biased (Basu and Meltzer 2005). Hence, an ideal approach should recognize that the welfare impact of informal care (IC) extends beyond the direct effects on the care recipients, and should include the indirect effects on caregivers (Bobinac et al. 2011). This paper draws on nationally representative individual data to estimate the economic value of informal caregiving, based on its impact on caregivers' life satisfaction, considering both the negative and, at times, potentially positive effects of caregiving to the caregiver's wellbeing. Additionally, our approach allows for a comprehensive consideration of the impact of both the short and long term effects of caregiving on the wellbeing of caregivers. That is, whether in computing our wellbeing‐based value of IC we consider an individual’s permanent income (income for the entire period of analysis) or, the current income of the year.

Informal caregiving can be costly to caregivers as they tend to spend less time on paid work and leisure (European Commission 2021), exhibit increased morbidity (Vitaliano, Zhang, and Scanlan 2003), stress (Bugge, Alexander, and Hagen 1999), depressive symptoms (Hajek, Kretzler, and König 2021; Pirraglia et al. 2005), and anxiety (Pirraglia et al. 2005; Sklenaroya et al. 2015). Caregivers generally earn lower wages than non‐caregivers (Colombo and Mercier 2012) and tend to retire earlier (Lilly, Laporte, and Coyte 2007; Costa‐Font and Vilaplana‐Prieto 2023). Furthermore, caregivers' burden entails opportunity costs, and externalities to family members (Bobinac et al. 2010; Hurley and Mentzakis 2013). However, under certain circumstances, IC can be beneficial to caregivers wellbeing (Brouwer et al. 2005) if they benefit from the experience of providing IC, either in terms of fulfillment of a social norm and personal development, as well as from the strengthening of their emotional ties with the care receiver (Butcher, Holkup, and Buckwalter 2001; Quinn, Clare, and Woods 2012; Joling et al. 2016).

Nonetheless, in estimating the economic value of IC, the estimation method employed should be sensitive to caregiver preferences (de Meijer et al. 2010). Accordingly, in this paper we estimate the net effect of caregiving on individuals' subjective wellbeing, following a welfare or social perspective to care valuation. The consideration of wider wellbeing effects of caregiving is important insofar as the estimation method can give rise to significant differences in the welfare effects of different long‐term care financing designs (Hoefman, van Exel, and Brouwer 2013).

We contribute to the literature as follows. First, we draw on wellbeing methods and longitudinal data to estimate the compensating surplus (CS) required to restore informal caregivers to the same level of life satisfaction as non‐caregivers both in the short (using an annual income transfer) and longterm (using a permanent income transfer during the period 2007–2020). Additionally, we retrieve an estimate of the cost per hour of care for 10 European countries, across gender and co‐residency status. Second, unlike previous studies, our estimates draw on an instrumental variable strategy to estimate the causal effect of the provision of IC on the well‐being of caregivers and co‐residential caregivers. This is important as otherwise the estimates can be biased by the presence of omitted variables and caregivers selection as well as reverse causality. Third, we show evidence of the robustness of our estimates across different datasets, specifically the use of the Survey of Health, Ageing and Retirement in Europe (SHARE) and the European Quality of Life Survey. Finally, a noveltry of the paper lies in the consideration of informal care hours from wave 8 of SHARE to estimate the value of IC in terms of €/per hour. Previous studies focus on specific illnesses or provide aggregate estimates instead.

We find evidence of an average 7 percentage point (pp) reduction in caregivers life satisfaction (42pp for co‐resident caregiver) resulting from the provision of IC. Our estimates suggest that the individual short‐term compensating surplus (CS) amounts to €13,101 on average (ranging between €28,196 in Spain and €7230 in Sweden). When compared to a country's GDP per capita, our estimates range from a maximum of 4.2% in France and Spain to a minimum of 0.8% in Germany and 1.3% in Sweden. These estimates are in line with previous studies suggesting that the replacement costs of IC in Europe account for approximately 3%–4% of GDP (Ekman et al. 2021).

The long‐term CS for the period 2007–2020 is estimated at €211,365 (ranging from €350,367 in Spain and €279,499 in France, and €116,646 in Sweden and €148,735 in Germany). These estimates suggest that caregivers may experience caregiving as partially rewarding, or they adapt to caregiving as they engage in providing informal care for longer periods. Lastly, the average CS per hour of care is estimated at €9.55 (ranging from €22.09 per hour in Switzerland and €4.97 per hour in Spain).

Next, we discuss the study background, including the main issues at stake in estimating the costs of IC and the literature on the effects of caregiving on life satisfaction. Section three describes the data and empirical strategy used, section four reports the results, heterogeneity and robustness and a final section concludes.

## 2 Background

*Methods for informal care valuation*. The elicitation of the social value of IC involves valuing intangible losses such as fatigue, emotional burden and the impact on the caregiver's work and social life. However, when valuing the time spent on informal care (IC), a key challenge is determining how to quantify the value of that time. A major issue includes the difficulty of separating care activities from other common duties when making such estimations. The literature typically considers two ways of approaching this problem. A common strategy follows the so‐called “revealed preference” methods, which assume that the costs of IC can be inferred by observing the behavior of individuals in markets closely related to the “good” of interest, which include proxy good and opportunity cost approaches. However, such methods do not directly consider individual preferences. Instead, they assume that such preferences are inferred from choices made by caregivers themselves. An alternative approach lies in the use of “stated preference” methods, which elicit respondents' preferences regarding hypothetical or actual caregiving scenarios (e.g., contingent valuation method, conjoint analysis, discrete choice experiments and the well‐being method).

Among revealed preference methods, the “proxy good” method assumes that the value of time invested in caregiving can be estimated by the cost of hiring the services of a professional caregiver in the market. However, the main objection to this method is that the value assigned to care bears little relation to the value of the inputs consumed. Alternatively, the opportunity cost method estimates the monetary value of the best alternative use of the time spent caring for the cared‐for person. However, such monetary values largely depend on the alternative use the caregiver wishes to make of the informal care time provided (e.g., leisure, paid or unpaid work time). A practical advantage is that the caregiver has to distinguish between the different types of normal uses of the time she sacrifices to supply care. However, such time allocation is unknown ex‐ante, and as discussed in Posnett and Jan (1996), the shadow price of unpaid work may differ from its marginal wage. Furthermore, it is challenging to assign an appropriate wage for some groups of carers who might be retired or never employed. Hence, both methods elicit essentially incomplete cost‐of‐time estimates that tend to ignore the preferences of caregivers and care recipients (Van den Berg et al. 2005a).

Given these limitations, alternative stated methods are recommended for the monetary valuation of IC, including contingent valuation (Van den Berg et al. 2005c; De Meijer et al. 2010) as well as the wellbeing valuation methods (Van den Berg and Ferrer‐i‐Carbonell 2007) used in this paper. The alternative to wellbeing mehods include conjoint analysis (Van den Berg et al. 2008) and discrete choice experiments (Mentzakis, Ryan, and McNamee 2011). However, both methods draw values from hypothetical rather than actual scenarios.

The contingent valuation method simulates a hypothetical market in which the individual welfare loss is estimated in terms of the income she would be willing to forgo in the face of changes in her welfare level resulting from the provision of care (Costa‐Font 2017). Preferences are elicited by comparing two states of nature that change an individual utility function.^1^ The drawback of this method includes the potential for protest responses and double counting if the caregiver considers both her preferences and the health of the person being cared for when evaluating the utility effects of providing care (Van den Berg et al. 2004). However, an advantage is that it considers the value of improved health and well‐being independently of the impact on productivity, alongside the indirect and intangible costs. Protest responses can be reduced by using conjoint analysis or discrete choice experiments, where individuals make choices from a sequence of hypothetical scenarios, which are defined in terms of different attributes and levels.^2^ Compared to the contingent valuation method, respondents do not directly elicit a monetary value. Instead, values are inferred from trade‐offs between different dimensions of the presented scenarios, which ameliorate the risk of strategic and protest responses (Van den Berg et al. 2005a). However, one of the main limitations of these two methods is that they do not provide accurate individual‐level valuations (Lancsar et al. 2013) and can be cognitively demanding to elicit.^3^

Finally, the well‐being method used in this study assesses the total impact (costs and benefits) of IC on the caregivers' well‐being (Van den Berg and Ferrer‐i‐Carbonell 2007). The value assigned to an hour of informal care represents the monetary compensation required to maintain the same level of well‐being of the informal caregiver.^4^ An important advantage of well‐being methods is that data collection typically experiences minimal non‐response, it is sensitive to individual caregivers preferences, and targets a sample of informal caregivers, without requiring a prior identification of such caregivers, thus avoiding potential sample selection bias. Our contribution in this field is to use the latter welfare assessment method but, in addition, and unlike previous studies, we draw on longitudinal evidence from several countries, and we consider the endogeneity of caregiver selection. We use an instrumental variable (IV) approach to correct for the potential endogeneity of IC on subjective wellbeing estimates.

*Subjective well‐being and informal care*. Life satisfaction is one of the most frequently adopted proxy indicators of subjective well‐being (Diener, Lucas, and Oishi 2002). Well‐being is a multidimensional concept that captures both perceived (e.g., social norm fulfillment) and objective (e.g., income, hour of care) components (Orgeta, Lo Sterzo, and Orrell 2013). Furthemore, In this particular study, it allows comparing the wellbeing of caregivers and non‐caregivers (George and Gwyther 1986).

Measuring wellbeing of caregivers is important becauase the supply of care can give rise to a significant burden on caregivers wellbeing, even though caregiving plays an essential role in enabling older individuals to age in place (Mitchell et al. 2015; Willemse et al. 2016; Costa‐Font, Elvira, and Mascarilla‐Miró 2009; Costa‐Font and Vilaplana‐Prieto 2022). As a result, caregivers tend to report lower levels of life satisfaction compared to non‐caregivers (Ha et al. 2008; Wagner and Brandt 2018, Pearlin et al. 1990, Costa‐Font, Jiménez‐Martín, and Vilaplana‐Prieto 2022). However, at times, the provision of care can give rise to both beneficial and detrimental effects (Lin, Fee, and Wu 2012; Wang et al. 2018), and such effects arise simultaneously (Pinquart and Sörensen 2005), and are likely not to remain stable over time, as caregivers might adapt to their caregiving role in the longer‐term (Sugihara et al. 2004; Koerner and Kenyon 2007). The consideration of subjective wellbeing as a measure of welfare allows integrating all those effects in on single outcome.

*Gender differences*. Gender differences are important as women are more likely to become caregivers (Billaud and Gramain 2014), though female caregivers' life satisfaction increases over time, and caregiving women report significantly higher levels of life satisfaction than caregiving men (Bookwala 2009).

Nonetheless, some estimates report evidence of gender‐specific differences in caregiving tasks that might influence the effects of IC on wellbeing. Indeed, male caregivers are more likely to perform more instrumental tasks while female caregivers are more likely to provide personal care and tasks with a more intense emotional involvement (Carroll and Campbell 2008). Furthermore, some studies document a greater willingness of women (especially daughters) to delegate part of caregiving duties to paid formal caregivers and to retain the roles of organizers and supervisors (Da Roit 2007).^5^ However, previous literature is split with regard to the differential impact of caregiving on caregiver well‐being. Some studies show a greater burden on female caregivers (Garlo et al. 2010; Kim et al. 2012), while others find no significant differences (Rosdinom et al. 2013).

Consistently, spousal caregivers tend to report lower levels of life satisfaction compared to their non‐caregiving counterparts (Lu, Liu, and Lou 2015; Di Lorito, Völlm, and Dening 2018). Raschick et al. (2004) compare rewards (feeling more life‐appreciation or self‐satisfaction) with costs (fatigue, lack of time for oneself, feeling overwhelmed), and document that women bear greater costs than men and that sons and daughters experience greater rewards than husbands and wives.^6^

*Care receivers' need*. The effect of being a caregiver in the literature, so far, is not conclusive. While Borg and Hallberg (2006) report a lower satisfaction among caregivers providing personal care, Schneider and Kleindiest (2016) document just the opposite effect. Similarly, there is no agreement in the literature on which need is more *burdensome* for the caregiver's wellbeing, the care receivers' behavioral symptoms (Gallagher et al. 2011) or their cognitive or functional decline (Zucchella et al. 2012). The main predictors of lower caregiver well‐being are care intensity or caregiving hours (Vaingankar et al. 2016), co‐residence with the dependent person (Kim et al. 2012), lack of support from other informal (Galvin et al. 2010) or formal caregivers (Zarit et al. 2011), scarcity of economic resources (Robinson et al. 2009) and lower educational level (Navaie‐Waliser et al. 2002).

*Effect of past experiences over life satisfaction*. A final important consideraton when using life satisfaction as a measure of outcome is that life satisfaction during adulthood is affected by experiences during childhood (Frijters, Johnston, and Shields 2014). The Grossman's (1972) model already accounts for the fact that child development does not occur as a cumulative function of family and environment, so that skills acquired in each period of life have a diminishing effect as the individual ages.^7^

Among potential adverse events, it is important to consider some features of a child's living environment may underpin trauma or chronic stress within the first 18 years of life (Hughes et al. 2017).^8^ Similarly, adverse events experienced during childhood can have a long‐term impact (Tani et al. 2016), increasing the likelihood of depression, anxiety, behavioral disorders, personality disorders, substance abuse, high‐risk behaviors and suicide (Afifi et al. 2010) and thus, resulting in lower life satisfaction (Mersky, Topitzes, and Reynolds 2013).^9^ In our analysis, we consider some adverse events such as malnutrition during childhood, since exposure to hunger in early life has been found to increase the risk for chronic disease and multimorbidity later in life (Fall 2013), as well as the likelihood of being overweight and depressed in adulthood and old age (Cui, Smith, and Zhao 2020).

## 3 The Data

The main dataset used in this study comes from the seven main waves of SHARE (1, 2, 4, 5, 6, 7 and 8), covering the period 2004–2020, and wave 3, also known as SHARELIFE, collected in 2009, which refers to a special wave with retrospective life and labor market information.^10^ Given that we cannot include the countries that joined SHARE after wave 3, we only use records of residents in 10 countries (Austria, Belgium, Czech Republic, Denmark, France, Germany, Italy, Spain, Sweden and Switzerland). Our inclusion criteria is that all individuals participate in both the regular waves 2, 4, 5, 6, 7 and 8, and in SHARELIFE to report retrospective information (which we will consider as invariant explanatory variables). The main advantage of SHARE is that it is a longitudinal survey which allows to control for unobserved heterogeneity, and it combines information on income, education, health status and family characteristics (current and retrospective).^11^

Given that we attempt to retrieve a measure of permanent or long‐term income, it is important to maximize both the number of observations and to ensure the use of standard measures across individuals. The final sample consists of 7368 individuals and 44,208 observations distributed roughly as follows: 67% non‐caregivers (56% non‐caregivers and non‐care recipients; 11% care recipients) and 33% caregivers (6% coresident caregivers and 27% non‐coresident caregivers) (see Table A1 in the Appendix for a detailed description).

*Dependent variable*. Life satisfaction is the main outcome of interest and reflects the degree to which he or she perceives that his or her aspirations (or goals) and achievements have been fulfilled (Fugl‐Meyer, Melin, and Fugl‐Meyer 2002). Life satisfaction is assessed with the following question: “*All things considered*, *on a scale of* 0 *to* 10, *where* 0 *denotes not satisfied at all and* 10 *denotes completely satisfied*, *how satisfied are you with your current life*?”. Respondents were asked to indicate their assessment on a visual analog scale.

*Informal caregiving*. We define a coresident caregiver using a binary variable that takes the value 1 if an individual responds affirmatively to the question “*is there someone living in this household whom you have helped regularly with personal care*, *such as washing*, *getting out of bed*, *or dressing?*”, and 0 otherwise. Similarly, non‐residential caregiving is measured as a binary variable that takes the value 1 if it responds affirmatively to the question: “*have you personally provided any kind of help listed on this card to a family member from outside the household*, *a friend or neighbor?”*, and 0 otherwise. In such a case, a limitation in the question lies in that it only refers to informal support in personal tasks, but not household tasks.

Figure A1 in the Appendix displays the density function of life satisfaction (LS) of caregivers and non‐caregivers stratified by gender. We observe that the density function for male and female caregivers is flatter and shifted to the left relative to that of non‐caregivers. When we turn to compare the caregivers to non caregivers by gender, the highest satisfaction corresponds to male non‐caregivers (and non‐care recipients), while the lowest satisfaction corresponds to female caregivers.^12^

Finally, Figure A3 in the Appendix displays the distribution of the sample by wave and type of caregiving status (see Appendix A). Table A3 reveals that the country with the highest percentage of caregivers is Denmark (44.8%), compared to only 18.6% in Spain. The opposite is true when we look at co‐residential caregivers (8.8% in Spain and 4.1% in Denmark).

*Current income and long‐term income*. To estimate a measure of permanent or long‐term income, we use the methodology proposed by Weiss (2012). Given that we cannot observe the individual income in the intermediate years between each two waves,^13^ we draw on the following procedure: (i) income estimates were retrieved using linear imputation when individuals receive the same source of income in two consecutive waves (e.g., retirement pension) and (ii) when individuals retire between two waves, we identify the month and year they retire and the earnings from the wave in which they were employed until retirement, and their retirement pension afterwards. We eliminate the effect of outliers by censoring observations that are above the 99th and below the 1st percentile of the distribution. Finally, we estimate the accumulated income over the entire period 2007–2020 discounted at an interest rate of 2%.^14^ More specifically, we estimate the long‐term individual income (2007–2020 adjusted by purchasing power parity (PPP) using the years 2020 base which we refer as PPP2020.^15^

*Endogeneity of income*. In estimating life satisfaction determinants, an emerging problem in the literature refers to the endogeneity of income. Individuals who are more satisfied with their lives tend to have higher incomes, and time‐varying unobservables can lead to both higher satisfaction and higher incomes (Clark, Frijters, and Shields 2008; Gardner and Oswald 2007). That is, there are several reasons to believe that the impact of income on life satisfaction is downward biased due to endogeneity, and this would lead to inaccurate estimates of compensation surplus (Clark, Frijters, and Shields. 2008), namely our measure of value of care as we explain below.

We use an instrumental variable strategy and consider a number of instruments. Bayer and Juessen (2015) found that long‐term changes in income exert a significant and sizable positive effect on life satisfaction, in contrast to short‐term shocks. Howley (2017) addressed this concern by using parental education as an instrument for earnings. However, parental education is likely to affect well‐being through a variety of other channels, such as social networks, wealth, or expectations. Lachowska (2017), using an economic stimulus tax rebate implemented in the U.S. in 2008, found significantly positive and robust effects of income on affect, but not statistically significant effects on life satisfaction. Finally, McNamee and Mendolia (2019) used as an instrument whether an individual had been affected by a positive income shock in the following 12 months.^16^

Our instrumental variable strategy draws on using the current income of the partner alongside the current income from other household members both adjusted by purchasing power parity in 2020 (PPP2020 and in logarithms) as our instruments. The intuition behind these instruments relies on previous studies (Luttmer 2005; Dolan and Metcalfe 2008), which estimate positive externalities on human capital and education (Benham 1974). In estimating short‐run income, our instruments are expressed in PPP2020, whilst long‐run estimates are retrieved following a linear imputation procedure to obtain the entire 2007–2020 sequence. Furthermore, in Section 6 (Robustness checks) we propose alternative instruments following Bartik (1991). Table A4 reports descriptive statistics for time‐invariant and time‐varying variables.

*Time‐invariant explanatory covariates*. We consider controls related to respondents' childhood and adolescence experienced adverse events which have been shown to impact health status and life satisfaction in adulthood. Among the adverse events we include individual responses to the question about the (i) happiest period in life, the period with most stress, the period with worst health, the period with greatest financial stress, as well as periods of hunger. In all cases, the length of the period and the age of the respondent at the beginning of this period are also included. Next, we consider whether the respondent (ii) had been a victim of persecution and discrimination; (iii) has ever experienced the following circumstances: lived in a children's home, been fostered with another family, evacuated or relocated during a war, lived in a prisoner war camp, lived in a prison, lived in a labor camp, lived in a concentration camp, stayed in a psychiatric hospital, patient in a tuberculosis institution, been homeless for 1 month or more. Furthermore, we consider the (iv) health status during childhood measured as their self‐reported health status, having been in hospital for more than one month, suffered an injury or accident that resulted in permanent disability, and finally we consider (v) family characteristics^17^ and (vi) their performance in education (performance in Maths and Language at age 10 and current level of education).^18^

*Time‐varying explanatory variables*. These variables include individual age, marital status, household size, size of municipality of residence, relationship with economic activity, current self‐reported health status, the Charlston Comorbidity Index and household wealth (adjusted by household size and PPP 2020). The health status of the respondents at the time of the survey is coded using the Charlson Comorbidity Index (CCI) (Charlson et al. 1987). Finally, following Diener and Suh 1998, we consider whether an individual is married, her higher educational attainment, and her highest annual income all of which are associated with her life satisfaction.

## 4 Empirical Strategy

Our specification considers life satisfaction (LS) as a function of a series of time‐varying variables (including current individual income and caregiving status) and time‐invariant variables (including past childhood experiences and long‐term individual income 2007–2020) as follows:

(1) $${LS}_{it}=\beta_{0}+\beta_{1}{\log\left(\text{CI}\right)}_{it}+\beta_{2}{\log\left(\text{LTI}\right)}_{i}+\beta_{3}{\text{IC}}_{it}+W_{it}^{\prime }\gamma +Z_{i}^{\prime }\delta +\zeta_{t}+\vartheta_{i}+\varepsilon_{it}$$

where ${LS}_{it}$ is the life satisfaction of individual *i* at time *t*, *CI* is the current income of the individual *i* at time *t*, *LTI* is the long‐term or permanent income of the individual *i* in the period 2007–2020, ${IC}_{it}$ denotes being an informal caregiver at time *t* (alternatively it will be replaced by two variables: coresident caregiver and non‐coresident caregiver), $W_{it}^{\prime }$ depicts time‐varying explanatory variables and $Z_{i}^{\prime }$ refers to time‐invariant explanatory variables, $\vartheta_{i}$ and $\zeta_{t}$ refer to individual and year fixed effects respectively.^19^ In the econometric specification, current and long‐term income are expressed in logarithms. We also include individual fixed effects in order to account for unobserved time‐invariant characteristics that could have an effect on life satisfaction, such as personality traits, cultural background or risk aversion. Hence, the unobserved heterogeneity parameter may be decomposed as follows:

(2) $$\vartheta_{i}=\alpha +\xi_{i}$$

The key issue at stake in estimating (1) is to identify consistent parameters for $\beta_{2}$ and δ imposing restrictions on $\vartheta_{i}$ as in (2). We draw the approach proposed by Pesaran and Zhou (2018), also known as 'fixed effect filtering (FEF)' model, which provides a two‐step estimation method retrieving consistent estimates of time invariant variables when individual fixed effects are assumed to be correlated with other regressors in the model.^20^

In the first step, a fixed effects model is estimated, but only using the time‐varying regressors ($\beta_{1}$ and γ). From this estimation, we retrieve the residuals as follows: ${\hat{u}}_{it}={LS}_{it}-{\hat{\beta }}_{1}{\log\left(\text{CI}\right)}_{it}-{\hat{\beta }}_{3}{\text{IC}}_{it}-W_{it}^{\prime }\hat{\gamma }$. In the second step, we estimate our equation of interest using the mean of the residuals and the time‐invariant regressors. Therefore, this second step may be regarded as a between panel data estimation in which the time‐varying effects are “filtered out”. Since the time‐invariant variables are uncorrelated with the unobserved fixed effects, the Pesaran and Zhou (2018) model provides consistent estimators.^21^

When one or more of the time‐invariant variables are endogenous, an IV version of the FEF estimator is also provided by Pesaran and Zhou (2018). The advantages of this estimator (FEF‐IV) lie in not requiring a subset of time‐varying regressors to be exogenous (as in Hausman and Taylor, 1981) and being robust to residual serial correlation and heteroscedasticity of errors. For robustness purposes, we proceed by performing the usual instrument validation tests and alternative instruments (see section on “Robustness checks").

*Endogeneity of IC*. As discussed earlier, IC is endogenous in our model of life satisfaction. Therefore, we need instrumental variables to correct for such potential endogeneity.^22^ Instruments should be correlated with the decision to provide informal care, without directly influencing life satisfaction of the potential caregiver. In the empirical literature, we find several potential instruments. Lo Sasso and Johnson (2002) used the number of adult children and the presence of a coresident daughter who has no children as instruments. Alternatively, Van Houtven and Norton (2004) reported the number of children and whether the oldest child is a daughter, and similarly, Coe and Van Houtven (2009) chose number of children in the family, percent of children who are girls and eldest child in the family being a daughter. Other studies include Barnay and Juin (2016) who instrumented IC using the proportion of daughters, having one child who is not parent him/herself, having one child being single, having one child living nearby; and finally, Wu et al. (2018) employed the number of children and the age of older children as instruments for IC.

Throughout the litertaure the number of daughters has been used as a recurrent instrument. It assumes that on the margin, the presence of a daughter increases the likelihood of informal care provision when need arises (Bolin et alii, 2008; Bonsang 2009; Barnay and Juin 2016). However, the effect of daughter availability on care differs across European countries (Bonsang and Costa‐Font 2023). For this reason, we use not only the number of daughters as instruments, but also the number of sons. Birth order variables have been considered for two reasons: (i) older siblings play an instructive role since they are more cognitively and socio‐emotionally mature than their younger siblings (van Berkel et al. 2023) and (ii) in some countries there is a cultural norm according to which older children are “more responsible” for parental care (Zarzycki et al. 2023). These instruments not only consider the availability of informal caregivers in the “*i*” dimension but also in the “it” dimension since changes in these instruments occur throughout the period under consideration. We found that there were 1960 deaths of children (4.43% of the sample). Consequently, our final set of instruments for informal care includes the *number of daughters*, the *number of sons*, *being the eldest son*, *the youngest son* and *being a single child*.

*Estimation of the Compensating Surplus*. Our measure of compensating surplus (CS) is obtained following the procedure proposed by Frey, Luechinger, and Stutzer (2009):

(3) $${CS}_{\text{current}\;\text{income}}=\bar{CI}\left(1-\exp\left(\frac{\beta_{3}}{\beta_{1}}\right)\right)$$

(4) $${CS}_{\text{long}-\text{term}\;\text{income}}=\bar{LTI}\left(1-\exp\left(\frac{\beta_{3}}{\beta_{2}}\right)\right)$$

where $\beta_{1},\beta_{2}$ and $\beta_{3}$ are the estimated coefficients from Equation (1) and $\bar{CI}$ and $\bar{LTI}$ denote the mean of CI and LTI, respectively. We believe that it is important to distinguish between CS associated with current income and permanent or long‐term income as expressed in Equations (3) and (4) insofar as the temporal dimension of informal care provision can entail adaptation effects that affect the value of our estimates.^23^

## 5 Results

*First‐step estimates*. Table B1 in the appendix addresses the question of caregiver selection, namely whether the probability of being a caregiver, both coresident or non‐coresident caregiver, is explained by our instruments. We report the first‐step regressions for both current and long‐term individual income too.^24^ In running such regressions, we consider a number of covariates such as age, sex, marital status, household size, level of education and country fixed effects as displayed in Table B1.

As expected, all five instruments are significant and reveal the expected sign. Being an only child increases the probability of being a caregiver by 14.4 percentage points (pp), and more specifically 9.3pp for men and 18.3pp for women. Being the eldest child increases the probability of being a caregiver by 10.6pp, and the number of brothers increases such probability by 1.8pp. In contrast, being the youngest child decreases this probability by 9.1pp, and the number of sisters decreases it by 10pp.^25^ According to van Berkel et al. (2023), older brothers take the lead in sibling interactions and often play an instructive role, as they are more cognitively and socioemotionally mature than their younger siblings (van Berkel et al. 2023). Additionally, Morosow and Kolk (2020) point out that: (i) daughters also face the duality of being mothers of their immediate family at home and daughters of their parents and (ii) older daughters tend to be more likely to develop caring and responsible personality traits. Social norms might be more influential on the older daughter with the idea that first‐born daughters are more likely to adopt traditional female gender roles than later‐born sisters. Similarly, all instruments for current individual income and long‐term individual income are significant and exhibit a positive sign. Estimates without using an instrumental variable strategy are reported in Table B1 in the appendix for comparison.^26^

*Fixed effects filtered model*. Table B2 in the appendix displays the estimates for the FEF model for the total sample and Table B3 in the appendix displays the results for the main variables for each of the countries. The results of the fixed effects model with time varying regressors suggest that: (i) being a caregiver reduces life satisfaction, with the impact being higher for coresident caregivers and for women; (ii) current individual income (PPP2020) is significant and positive, and is higher for men; and (iii) being unemployed, undertaking housework or exhibiting a higher value of the CCI exerts a negative impact on satisfaction.

The results of the between model with time‐invariant regressors show that, as expected, the effect of long‐term individual income is positive (and higher for men).^27^ Conversely, having experienced unhappy times in the past is associated with lower life satisfaction in the present.^28^ We find that whether an individual has been born in another country, experienced episodes of persecution (−10.6pp) or discrimination exerts a negative effect on their life satisfaction.^29^ Table B4 in the appendix reports the comparison of both short‐ and long‐term income effects with and without correcting for instrumental variables. Consistently, the estimates without IV corrections show lower standard errors, but the magnitude of the coefficients is higher.

### 5.1 Compensating Surplus (CS)

Table 1 shows the following estimates: (i) the individual short‐term CS in euros (PPP 2020); (ii) the aggregate short‐term CS (million €; PPP2020), (iii) the aggregate short‐term CS as a percentage of GDP; (iv) the individual short‐term CS as a percentage of GDP per capita and (v) the individual short‐term CS as a percentage of average wage (by gender). The average individual short‐term CS amounts to €13,101 (with a maximum value in Spain: €28,196 and a minimum value in Sweden: €7230). The CS for co‐resident and non‐co‐resident caregivers amounts to €36,693 and €9,324, exibiting a significant difference between them of €27,369.^30^ When we estimate the CS for all caregivers in each country, the maximum value corresponds to France (90,157 million €) and the minimum corresponds to Denmark (3474 million €). As a percentage of GDP (PPP2020), we find the highest values in France (4.22%) and Spain (3.27%), and the lowest in Germany (0.85%) and Sweden (1.27%).^31^ Finally, the ratio of the CS to the average annual wage ranges between 92.6% for Spain and 84.5% in France and 22.6% for Sweden.^32^

**TABLE 1:** Short‐term compensating surplus (CS) per individual, aggregate CS, percentage of aggregate CS with respect to GDP, percentage of individual CS with respect to per capita GDP, percentage of individual CS with respect to annual average wage.

	Total			Men			Women		
	Caregiver	Coresid. caregiver	No coresid caregiver	Caregiver	Coresid. caregiver	No coresid caregiver	Caregiver	Coresid. caregiver	No coresid caregiver
Individual CS (Euros PPP 2020)									
**Average**	**13.101**	**36.693**	**9.324**	**17.362**	**49.583**	**12.227**	**11.739**	**33.972**	**8.165**
Austria	13,367	35,317	8322	21,464	66,179	11,186	11,506	28,223	7663
Belgium	14,830	49,209	8626	17,624	55,901	10,716	14,326	48,002	8249
Czechia	12,394	28,131	7941	20,091	47,368	12,372	10,216	22,687	6687
Denmark	8846	45,529	5055	11,828	67,578	6066	8538	43,250	4950
France	17,324	49,328	10,314	21,247	55,541	13,734	16,465	47,967	9565
Germany	9219	25,418	5714	11,712	34,740	6730	8679	23,401	5494
Italy	14,003	18,725	11,147	19,817	27,016	15,463	10,487	13,710	8537
Spain	28,196	32,434	24,806	33,113	39,243	28,209	22,051	23,923	20,553
Sweden	7230	36,635	4189	11,755	71,897	5535	6762	32,988	4050
Switzerland	14,161	34,465	11,970	18,216	23,758	17,618	13,723	35,621	11,361
Aggregate CS (million € 2020)									
Austria	3756	702	3054	1451	210	1242	2304	492	1812
Belgium	13,099	2003	11,097	5752	925	4827	7347	1077	6270
Czechia	6170	1361	4809	2128	441	1687	4042	920	3122
Denmark	3474	325	3148	1542	127	1415	1932	198	1734
France	90,157	16,201	73,957	37,868	7508	30,361	52,289	8693	43,596
Germany	26,063	4636	21,428	11,496	1866	9631	14,567	2770	11,797
Italy	41,778	15,745	26,034	14,561	5300	9261	27,218	10,445	16,773
Spain	38,510	21,394	17,115	15,500	9018	6482	23,010	12,376	10,633
Sweden	4820	452	4368	1988	142	1846	2832	310	2523
Switzerland	8736	851	7886	3373	281	3092	5364	570	4794
Percentage aggregate CS with respect to of GDP (2016)									
Austria	1.12	0.21	0.91	0.43	0.06	0.37	0.69	0.15	0.54
Belgium	3.18	0.49	2.69	1.39	0.22	1.17	1.78	0.26	1.52
Czechia	2.06	0.45	1.60	0.71	0.15	0.56	1.35	0.31	1.04
Denmark	1.50	0.14	1.36	0.66	0.05	0.61	0.83	0.09	0.75
France	4.22	0.76	3.46	1.77	0.35	1.42	2.45	0.41	2.04
Germany	0.85	0.15	0.70	0.37	0.06	0.31	0.47	0.09	0.38
Italy	2.49	0.94	1.55	0.87	0.32	0.55	1.62	0.62	1.00
Spain	3.27	1.82	1.45	1.32	0.77	0.55	1.95	1.05	0.90
Sweden	1.27	0.12	1.15	0.52	0.04	0.48	0.74	0.08	0.66
Switzerland	2.11	0.21	1.90	0.81	0.07	0.75	1.30	0.14	1.16
Percentage of individual CS with respect to GDP per capita (2016)									
Austria	21.21	56.75	13.04	61.57	189.82	32.09	33.00	80.95	21.98
Belgium	24.45	79.97	14.43	54.57	173.10	33.18	44.36	148.64	25.54
Czechia	28.35	64.26	18.18	84.16	198.43	51.83	42.80	95.04	28.01
Denmark	14.18	75.69	7.82	33.95	193.95	17.41	24.50	124.13	14.21
France	31.90	88.75	19.44	73.26	191.52	47.36	56.78	165.40	32.98
Germany	14.97	42.08	9.10	35.25	104.55	20.25	26.12	70.42	16.53
Italy	25.00	33.17	20.06	72.29	98.55	56.40	38.25	50.01	31.14
Spain	52.35	58.90	47.12	131.21	155.51	111.78	87.38	94.80	81.45
Sweden	12.43	66.29	6.86	34.46	210.75	16.22	19.82	96.70	11.87
Switzerland	31.30	42.12	52.29	41.03	53.51	39.68	30.91	80.23	25.59
Percentage of individual CS with respect to annual wage (2016)									
Austria	38.57	101.90	24.01	61.93	190.95	32.28	33.20	81.44	22.11
Belgium	36.21	143.34	16.88	44.92	164.20	23.39	34.64	139.58	15.70
Czechia	52.39	118.92	33.57	84.93	200.24	52.30	43.19	95.90	28.27
Denmark	25.56	131.57	14.61	34.18	195.28	17.53	24.67	124.98	14.30
France	70.04	180.95	45.74	83.63	202.48	57.60	67.06	176.23	43.15
Germany	22.95	72.06	12.32	30.51	100.32	15.40	21.31	65.94	11.66
Italy	46.21	63.48	35.77	67.48	93.81	51.55	33.35	45.14	26.22
Spain	88.01	74.54	104.84	107.54	88.05	131.89	63.59	57.65	71.03
Sweden	21.28	107.84	12.33	34.60	211.63	16.29	19.90	97.10	11.92
Switzerland	32.05	78.00	27.09	41.22	53.76	39.87	31.06	80.61	25.71

*Note:* This table reports the short term compensating surplus (CS) estimated as the monetary transferred required to hold utility constant for caregivers in the short‐term (1 year). Annual wage (PPP2020): remuneration in cash and in kind paid to employees, as a rule at regular intervals, for time worked or work done together with remuneration for time not worked, such as annual vacation, another type of paid leave or holidays. Earnings exclude employers' contributions in respect of their employees paid to social security and pension schemes and also the benefits received by employees under these schemes. Earnings also exclude severance and termination pay.

*Source*: Prepared by the authors with Eurostat and ILOSTAT.

Table 2 displays: (i) the individual long‐term CS, (ii) the aggregate long‐term CS with respect to GDP (Euro PPP; 2007–2020) and (iii) the percentage of individual long‐term CS with respect to GDP per capita (Euro PPP; 2007–2020). The long‐term CS amounts to €211,365 (with a maximum value in Spain: €350,367 and France: €279,499; and a minimum value in Sweden: €116,646 and Germany: €148,735). To gain a clearer understanding of these figures, we compare the compensating surpluses (CS) as a share of GDP over both short (using current income) and long term (using permanent income). In Belgium, we note that the long‐term CS exceeds the short‐term CS by 0.48pp, whereas in Italy and Spain such difference is barely 0.06pp and 0.02pp, respectively. These differences are suggestive of a large cross‐country heterogeneity, and also suggest that over a span of 14 years, the significance of CS may not be as pronounced as initially anticipated.^33^

**TABLE 2:** Long‐term compensating surplus (CS) per individual, percentage of aggregate CS with respect to GDP, percentage of individual CS with respect to per capita GDP.

	Total			Men			Women		
	Caregiver	Coresid. caregiver	No coresid caregiver	Caregiver	Coresid. caregiver	No coresid caregiver	Caregiver	Coresid. caregiver	No coresid caregiver
Individual compensating surplus 2007–2020 (Euros PPP 2020)									
**Average**	**211,365**	**586,125**	**148,935**	**277,347**	**792,031**	**195,314**	**187,516**	**542,673**	**130,427**
Austria	215,660	564,155	132,933	342,871	1,057,138	178,691	183,797	450,839	122,415
Belgium	239,263	786,064	137,788	281,519	892,952	171,171	228,846	766,774	131,763
Czechia	199,958	449,356	126,849	320,930	756,653	197,629	163,191	362,397	106,820
Denmark	142,718	727,276	80,745	188,947	1,079,486	96,900	136,387	690,871	79,075
France	279,499	787,961	164,753	339,391	887,204	219,389	263,015	766,222	152,784
Germany	148,735	406,023	91,278	187,088	554,932	107,510	138,641	373,808	87,766
Italy	225,916	299,107	178,064	316,553	431,560	246,998	167,513	219,000	136,373
Spain	350,367	396,255	518,100	528,941	450,603	626,864	352,243	328,320	382,146
Sweden	116,646	585,205	66,913	187,770	1,148,484	88,413	108,016	526,951	64,690
Switzerland	228,472	550,548	191,210	290,977	379,501	281,427	219,212	569,001	181,477
Percentage of long‐term GDP (2007–2020)									
Austria	1.26	0.24	1.03	0.49	0.07	0.42	0.78	0.17	0.61
Belgium	3.66	0.56	3.10	1.61	0.26	1.35	2.05	0.30	1.75
Czechia	2.47	0.55	1.93	0.85	0.18	0.68	1.62	0.37	1.25
Denmark	1.78	0.17	1.61	0.79	0.06	0.72	0.99	0.10	0.89
France	4.73	0.85	3.88	1.99	0.39	1.59	2.74	0.46	2.29
Germany	0.97	0.17	0.80	0.43	0.07	0.36	0.54	0.10	0.44
Italy	2.55	0.96	1.59	0.89	0.32	0.57	1.66	0.64	1.03
Spain	3.29	1.83	1.46	1.32	0.77	0.55	1.96	1.06	0.91
Sweden	1.46	0.14	1.33	0.60	0.04	0.56	0.86	0.09	0.77
Switzerland	2.40	0.23	2.17	0.93	0.08	0.85	1.47	0.16	1.32
Percentage of GDP per capita (2007–2020)									
Austria	38.57	101.90	24.01	61.93	190.95	32.28	33.20	81.44	22.11
Belgium	46.21	153.34	26.88	54.92	174.20	33.39	44.64	149.58	25.70
Czechia	52.39	118.92	33.57	84.93	200.24	52.30	43.19	95.90	28.27
Denmark	25.56	131.57	14.61	34.18	195.28	17.53	24.67	124.98	14.30
France	60.04	170.95	35.74	73.63	192.48	47.60	57.06	166.23	33.15
Germany	27.95	77.06	17.32	35.51	105.32	20.40	26.31	70.94	16.66
Italy	51.21	68.48	40.77	72.48	98.81	56.55	38.35	50.14	31.22
Spain	112.01	98.54	128.84	131.54	112.05	155.89	87.59	81.65	95.03
Sweden	21.28	107.84	12.33	34.60	211.63	16.29	19.90	97.10	11.92
Switzerland	32.05	78.00	27.09	41.22	53.76	39.87	31.06	80.61	25.71

*Note:* This table reports the long term compensating surplus (CS) estimated as the monetary transferred required to hold utility constant for caregivers in the period 2007–2020. GDP per capita (2007–2020): is the sum of GDP per capita (PPP2020) discounted at the 2% interest rate for the period 2007–2020. GDP (2007–2020): is the sum of GDP (PPP 2020) discounted at the 2% interest rate for the period 2007–2020.

Although the aim of this paper is not to quantify the effect of long‐term care expenditure on life satisfaction, we extend our analysis to examine whether there is a relationship between CS and long‐term care expenditure. Table B5 in the Appendix B presents the results of the estimation of a model using long‐term care expenditure (€1000; PPP2020) as an explanatory variable. The coefficient shows that an increase of €1000(PPP) exerts a significant and positive effect on life satisfaction (0.046 or 4.6 percentage points (pp)), an effect that is stronger for women (0.064 or 6.4 pp) compared to men.

**FIGURE 1:**
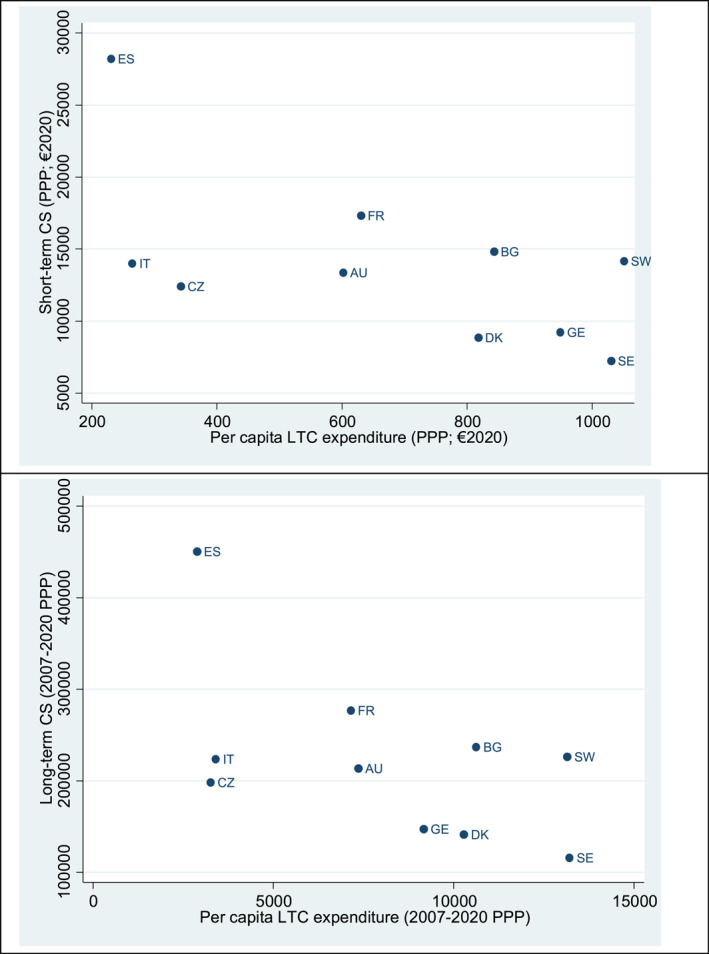
Individual short‐term and long‐term compensating surplus (CS) estimated plotted against average per capita long‐term care expenditure 2007–2020 adjusted by purchasing power parity (PPP). *Source*: Calculations using data from our own estimates and data from Eurostat Statistics | Eurostat (europa.eu). AU: Austria; BG: Belgium; CZ: Czech Republic: DK: Denmark; ES: Spain; FR: France; GE: Germany; IT: Italy; SE: Sweden; SW: Switzerland.

Given that in classifying long‐term care systems, the expenditure amount is always accounted for, next we examine whether combining the two variables can add value to the rankings of long‐term care systems. We do so by comparing short‐ and long‐term CS with respect to long‐term care expenditure. It turns out that the countries examined exhibit important differences in their long‐term care systems classifications, and we do not anticipate that two countries with similar levels of expenditure will show comparable levels of CS.^34^ Table B6 in the appendix consistently reports the estimates following the classification from Ilinca, Leichsenring, and Rodrigues (2015 2022) and Jiménez‐Martín and Vilaplana‐Prieto (2015).^35^ Figure 1 reports the individual short‐ and long‐term CS to per capita long‐term care expenditure.

Our CS estimations appear to inversely vary with national long‐term care expenditure estimates which capture the extent of formalization of caregiving in a country. Indeed, Figure 1 depicts three groups of countries: (i) low LTC expenditure and high CS (Spain) or moderate CS (Czech Republic, Italy); (ii) intermediate LTC expenditure and CS (Austria and France) and (iii) high LTC with low CS (Belgium, Denmark and Denmark) or intermediate (Sweden and Switzerland). In the classification reported in Table B6 in the Appendix, our results consider (i) differences in the type of family model in Europe which influences the funding of long‐term‐ care (Costa‐Font 2010; Costa‐Font and Vilaplana‐Prieto 2017; Costa‐Font, Jiménez‐Martínez, and Vilaplana‐Prieto 2018; Costa‐Font, Jiménez‐Martín, and Vilaplana‐Prieto 2022), since CS is considerably higher in Spain compared to Italy; our estimates identify significant differences in the standard care mix across countries (because Austria and France, and especially France, exhibit a higher CS than Belgium and Switzerland).^36^

### 5.2 Heterogeneity

Table 3 displays the heterogeneity of the individual short‐term CS estimates as a function of age, place of residence and level of education. Compared to the average individual CS, we observe that the CS describes an inverted U shape: it increases for the 60–69 years cohort (0.70%), but decreases among the youngest cohort (−1.58%), especially for non‐residents (−2.20%) and reveals a rise again among the oldest cohort (0.42%). Similarly, it exhibits a smaller effect in smaller cities and rural areas as well as a significantly larger value in the suburbs of large cities. In large cities, the CS decreases for co‐resident caregivers (−1.84%) but increases among non‐coresident caregivers (0.44%), and for such group the CS increases more among women compared to for men.

**TABLE 3:** Individual short‐term compensating surplus by age, place of residence and level of education.

	Total			Men			Women		
	Caregiver	Coresid. caregiver	No coresid caregiver	Caregiver	Coresid. caregiver	No coresid caregiver	Caregiver	Coresid. caregiver	No coresid caregiver
Individual short‐term CS (Euros PPP 2020)									
Average	13.101	36.693	9.324	17.362	49.583	12.227	11.739	33.972	8.165
Age									
50–59	12.893	36.254	9.119	17.180	49.044	12.021	11.558	33.780	7.986
60–69	13.193	36.565	9.370	17.454	49.509	12.258	11.839	33.882	8.221
70–79	13.098	36.559	9.360	17.376	49.202	12.261	11.706	33.942	8.188
80+	13.058	36.448	9.305	17.274	49.548	12.229	11.709	33.977	8.139
Size of municipality									
Big city	13.146	36.017	9.365	17.569	49.638	12.397	11.740	32.909	8.171
Suburbs/outskirts	13.358	38.039	9.445	17.545	51.837	12.271	12.043	34.938	8.325
Large town	13.103	36.692	9.341	17.317	50.543	12.170	11.776	33.456	8.225
Small town	13.095	36.755	9.327	17.346	49.078	12.235	11.729	34.271	8.161
Rural	13.033	36.526	9.284	17.313	49.203	12.197	11.650	33.949	8.110
Education									
No studies	12.441	35.149	8.990	16.719	47.825	11.993	11.101	32.533	7.826
Elementary	12.782	36.521	9.046	17.004	50.149	11.921	11.448	33.521	7.914
Secondary	13.193	37.273	9.345	17.415	49.357	12.219	11.843	34.942	8.192
Higher	13.594	38.998	9.608	17.790	52.131	12.460	12.284	36.285	8.478
Growth rate with respect to average CS (2007–2020)									
Age									
50–59	−1.58	−1.19	−2.20	−1.05	−1.09	−1.68	−1.54	−0.56	−2.19
60–69	0.70	−0.35	0.50	0.53	−0.15	0.25	0.85	−0.26	0.68
70–79	−0.02	−0.37	0.39	0.08	−0.77	0.28	−0.28	−0.09	0.28
80+	−0.33	−0.67	−0.20	−0.51	−0.07	0.01	−0.25	0.01	−0.32
Size of municipality									
Big city	0.35	−1.84	0.44	1.19	0.11	1.39	0.01	−3.13	0.08
Suburbs/outskirts	1.96	3.67	1.30	1.05	4.55	0.36	2.59	2.84	1.96
Large town	0.02	0.00	0.18	−0.26	1.94	−0.46	0.32	−1.52	0.73
Small town	−0.05	0.17	0.04	−0.09	−1.02	0.07	−0.09	0.88	−0.05
Rural	−0.52	−0.46	−0.43	−0.29	−0.77	−0.25	−0.76	−0.07	−0.68
Education									
No studies	−5.03	−4.21	−3.58	−3.70	−3.54	−1.91	−5.43	−4.24	−4.15
Elementary	−2.43	−0.47	−2.98	−2.06	1.14	−2.50	−2.48	−1.33	−3.07
Secondary	0.71	1.58	0.22	0.30	−0.45	−0.07	0.89	2.85	0.33
Higher	3.77	6.28	3.05	2.46	5.14	1.91	4.65	6.81	3.84

*Note:* this table reports the estimates of the CS retrieved using estimating Equation (1) restricting the sample by age, size of municipality and level of education, but including the other explanatory variables.

Finally, when we consider an individual's education attainment, and we document that the CS decreases for caregivers with no education (−5.03% for no education and −2.43% for primary education) but increases significantly among those with a higher education attainment (3.77%). In the latter case, the increase is higher for women (4.65%) than for men (2.46%). The opposite is true among caregivers with no or only elementary education. In fact, the greatest reduction in the CS is estimated for female caregivers.

### 5.3 Estimation of the CS per Hour

**FIGURE 2:**
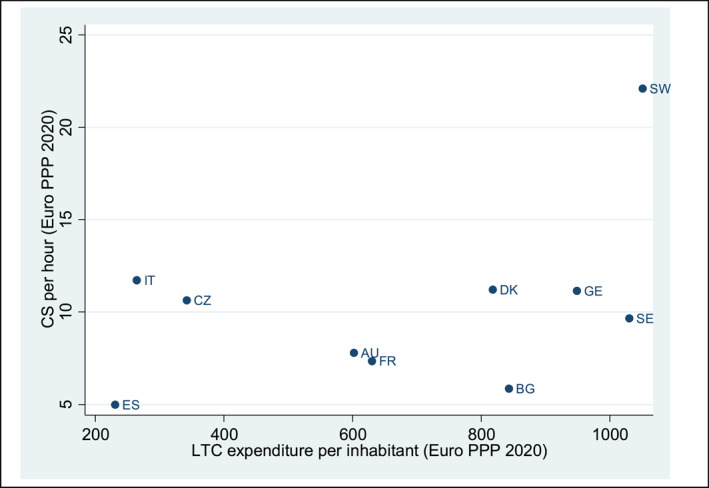
Relationship between individual CS per hour of informal care and per capita LTC expenditure. *Source*: Calculations using data from our estimates and data from Eurostat Statistics | Eurostat (europa.eu). AU: Austria; BG: Belgium; CZ: Czech Republic: DK: Denmark; ES: Spain; FR: France; GE: Germany; IT: Italy; SE: Sweden; SW: Switzerland.

Next, we estimate the CS per hour of informal care using wave 8 of SHARE. Unlike other studies, we retrieve CS estimates for each of the countries analyzed (as opposed to all countries as Schneider and Kleindienst (2016)) and our estimates are not disease‐specific.^37^ We have estimated Equation (1), considering the number of hours of informal caregiving instead of a binary variable, but we have drawn on the same set of instruments. Table 4 displays the average number of hours of IC and the CS per hour of caregiving (PPP2020), and Figure 2 illustrates the CS per hour alongside per capita long‐term care (LTC) expenditure. These results complement those observed in Figure 1, further reinforcing the conclusions regarding country differences.

**TABLE 4:** Daily caregiving hours and short‐term compensating surplus (€; PPP2020). Wave 8 of SHARE.

	Total			Men			Women		
	Caregiver	Coresid. caregiver	No coresid caregiver	Caregiver	Coresid. caregiver	No coresid caregiver	Caregiver	Coresid. caregiver	No coresid caregiver
Daily caregiving hours									
Average	3.76	4.55	3.79	3.72	5.00	3.78	3.80	4.25	3.79
Austria	4.71	7.18	5.40	6.00	13.50	7.67	3.65	3.57	3.55
Germany	3.65	4.11	3.28	3.44	4.50	3.24	3.93	3.89	3.34
Sweden	3.52	3.77	3.40	3.29	3.00	3.14	3.77	4.11	3.67
Spain	4.88	4.62	4.95	5.25	5.31	5.28	4.52	4.00	4.58
Italy	4.05	5.59	4.06	3.96	5.78	3.93	4.14	5.48	4.19
France	3.44	3.65	3.29	3.47	3.78	3.14	3.42	3.57	3.42
Denmark	3.42	3.92	3.39	3.28	3.64	3.31	3.60	4.14	3.50
Switzerland	3.50	3.20	3.54	3.40	3.17	3.40	3.60	3.25	3.67
Belgium	3.38	3.68	3.48	3.50	4.17	3.66	3.26	3.23	3.24
Czech Republic	3.65	4.89	3.52	3.61	6.00	3.50	3.70	4.50	3.55
CS per hour (Euros PPP 2020)									
Average	9.55	22.11	6.75	12.80	27.17	8.86	8.46	21.88	5.90
Austria	7.78	13.47	4.22	9.80	13.43	4.00	8.64	21.65	5.92
Germany	11.13	32.83	7.20	14.05	34.03	9.06	9.98	33.82	6.76
Sweden	9.64	20.45	6.40	16.74	43.26	10.79	7.42	15.12	5.00
Spain	4.97	27.01	2.80	6.17	34.85	3.15	5.18	29.62	2.96
Italy	11.72	24.18	6.96	14.71	26.34	9.58	10.91	23.96	6.25
France	7.34	19.07	4.76	9.24	25.19	5.87	6.96	17.95	4.40
Denmark	11.21	13.09	9.00	16.56	20.35	12.79	7.98	9.07	6.68
Switzerland	22.09	21.24	25.13	26.70	24.41	31.62	16.79	17.33	17.85
Belgium	5.85	27.27	3.30	9.20	47.27	4.14	5.68	27.97	3.42
Czech Republic	10.63	19.31	9.31	13.83	10.85	13.79	10.17	21.69	8.78

*Note:* This tble reports the cost in euros of the daily caregiving caost per hours and the hort term CS.

*Source*: own work using wave 8 of SHARE.

The average compensating surplus (CS) in PPP 2020 is of about 9.5 €/hour for all caregivers, 22 €/hour for coresident caregivers, and 6.7 €/hour for non‐coresident caregivers. As expected, the average compensating surplus is greater for men than for women, regardless of the type of caregiver. By country, the CS for caregivers ranges from 5 €/hour (Spain) to 22 €/hour (Switzerland), which also holds true for non‐coresident caregivers.^38^

Our estimates suggest that *co‐resident caregivers experience a greater burden on their wellbeing, which is explained by the* finding that there are no significant differences in the number of care hours, yet notable variations in caregiver support (CS) between co‐resident and non‐co‐resident carers (with higher CS observed for co‐resident carers in Table 1). This finding can be explained by more stringent demands on this type of caregivers , who are expected to address the need to provide care tasks on spot as they arise, which may also be more complex than those typically handled by non‐co‐resident carers.

## 6 Robustness Checks

Finally, in this section we perform three robustness analyses: (i) whether the estimates are robust to the use of an alternative instrument, namely a Bartik instrument to explain selection into caregiving; (ii) a placebo test to corroborate the suitability of our instruments for informal care; and (iii) the computation of short‐term CS using an alternative survey (European Quality of Life Survey).

### 6.1 Bartik Instrument for Individual Income

As an alternative to the instruments used we use a Bartik‐type instrument that combines employment rates at the national level across industries with differences in the initial industry structure across regions (Bartik 1991). Our alternative instrument is a weighted average of national‐level employment rates in each of the 14 industries,^39^ where the weights are the fraction of the working‐age population employed in each industry in the year prior to the beginning of the sample period. The national employment rates are exogenous to the characteristics of workers in each region (NUTS‐2),^40^ since the regions are small relative to the overall size. The initial industrial structure of a region is likely to be correlated with the characteristics of its workers, which poses a threat to the validity of the instrument. However, the initial industrial structure is, by definition, time invariant at the region level, so we can address this threat by controlling for region‐level fixed effects, which we do in all regressions. Table C1 in the appendix shows the short‐term and long‐term CS obtained using the Bartik instruments, and Figure C1 shows the difference between average CS and long‐term CS (short‐term from Table 1 and long‐term from Table 2) and the CS using Bartik. On average, the Bartik instrument reports similar estimates although overestimates the CS (2.58% in the short‐term and 2.18% in the long‐term). Hence, we conclude that our initial estimates are reliable and slighly conservative compared to the estimates using a Bartik instrument.

### 6.2 Placebo Test for Informal Care Instruments

To ensure the robustness of our analysis, we examine whether the instruments used to instrument caregiving status exert a direct impact on life satisfaction.^41^ Hence, to address these challenges, we have evaluated the impact of our set of instruments (number of brothers, number of sisters, oldest child, youngest child, only child) on life satisfaction while controlling for other explanatory variables in Table C2. Reassuringly, we find that except for the number of siblings (which is only significant at the 10% level in the female sample), the instruments are not statistically significant. These findings remain consistent irrespectively of the instruymental variable strategy employed.

Next, we performed other placebo tests using the income of the partner and other household members. The results (Table C3 in the appendix) show that both variables fail to exert a significant effect on life satisfaction. Although these results may seem somewhat surprising, estimates are actually in line with previous research (Howell and Howell 2008; Easterlin 2015; Kushlev, Dunn, and Lucas 2015). Instead, an individual's subjective wellbeing depends on that of others wellbeing drivers (e.g., economic status) or on their own wellbeing in the past. Therefore, rather than the income of the partner or other household members, we include in our specification the individual's income relative to that of the partner or other household members. This is known as the “relative income hypothesis,” which states that what really matters to an individual’s wellbeing is their income relative to that of others (Huang, Wu, and Deng 2016) or to the person's own income in the past (Ekici and Koydemir 2016).

### 6.3 Alternative dataset: European Quality of Life Survey

Finally, to assess the reliability of our short‐term CS estimates, we compared our estimates with the last wave of the European Quality of Life Survey (EQLS), corresponding to 2016. Appendix D describes the characteristics of the survey and the details of the estimation process. The advantage of the EQLS is that it allows disentangling the CS of older and younger caregivers, although we cannot distinguish between co‐resident and non‐corresident caregivers, nor the long‐term CS. We compare the CS of caregivers over 50 years of age using the two different sources of information available (SHARE and EQLS), and we compare our estimates with those of previous studies (Peña‐Longobardo and Oliva‐Moreno 2022) which draw only on opportunity cost methods (See Appendix D for a detailed description.).

Table 5 reports the ratio of CS to GDP, which can be compared to the value of informal care relative to GDP (following estimates using the opportunity cost method carried out by Peña‐Longobardo and Oliva‐Moreno 2022). We find that the CS relative to GDP is higher than the value of informal care using opportunity cots methods in all countries, ranging between 0.21pp (Belgium) and 1.82pp (Czech Republic), even though in most countries it is half a percentage point higher.^42^ The estimated higher value of (short‐term) CS suggests that there are relevant effects that are not captured by the opportunity cost method.

**TABLE 5:** Comparison of compensating surplus using SHARE and European Quality of Life Survey and estimation of the value of informal care (see Appendix D for an extension of the results).

	Compensating surplus with respect to GDP (using SHARE)		Compensating surplus with respect to GDP (2016) age: 50+	Estimation value informal care with respect to GDP (2016) Peña‐Longobardo et al. (2022)
	Short‐term	Long‐term		
Austria	1.12	1.26	1.02	1.90
Belgium	3.18	3.66	2.90	4.38
Czechia	2.06	2.47	1.93	2.05
Denmark	1.50	1.78	1.32	1.74
France	4.22	4.73	4.16	6.50
Germany	0.85	0.97	0.79	1.28
Italy	2.49	2.55	2.26	3.25
Spain	3.27	3.29	3.11	4.01
Sweden	1.27	1.46	1.18	1.20
Switzerland	2.11	2.40	—	—

*Note:* Columns (1) and (2) are retrieved from Tables 1 and 2. See Appendix C for detail of the explanation of compensating surplus using European Quality of Life Survey (2016). Estimations of the value of informal care with respect to GDP are retrieved from Peña‐Lonbgobardo et al. (2022) and refer to all informal caregivers, regardless their age. To value paid work time, they used the average gross hourly wage in purchasing power parity in each country, taking into account the caregiving hours provided by those caregivers who were employed. To value unpaid work time, they used the minimum gross hourly wage.

*Source*: Own work using SHARE and EQLS (2016).

## 7 Conclusion

Although informal caregiving (IC) is still the most common form of care for old age individuals in need of care in many European countries, its value is “hidden” in most estimates of long‐term care costs. Long term care expenditures only estimate the “observed” financial spending in paid care. However, estimating the true hidden costs of IC is far from straightforward insofar as the provision of care can exert both negative and positive effects on individuals' wellbeing. This paper draws on the application of the wellbeing‐based estimation method which account for all effects on wellbeing, and considers both short‐ and long‐term effects of caregiving on the life satisfaction (or subjective wellbeing) of caregivers. The primary contribution of the study is the application of a well‐being approach to the valuation of informal care across several countries, while considering the endogeneity of caregiving on life satisfaction. Specifically, we estimate the equivalent income transfer required for caregivers to attain the same level of life satisfaction as non‐caregivers, which we refer to as the compensating surplus (CS). We use longitudinal evidence from a number of European countries, and we draw on incremental variable approach to estimate an unbiased effect of income of both caregiving as well as current and permanent or long term income on life satisfaction. The latter allows us to estimate the effect of informal caregiving on subjective wellbeing, and an unbiased estimate of the CS. Hence, the CS can in turn be compared with estimates of the value of informal care drawn from other methods such as replacement methods and opportunity cost estimates.

We document evidence of a net negative effect of informal care (IC) on life satisfaction, which is estimated to be 7 percentage points (pp) or about an average 1% reduction among the entire caregiver’s sample, and 42pp or 6% reduction in life satisfaction among residential caregivers.^43^ Our estimates indicate that the compensating surplus (CS) as a percentage of GDP ranges from 4.2% in France to 3.3% in Spain. On the lower end of the country distribution, we estimate a CS of 0.85% in Germany and 1.3% in Sweden. Furthermore, we document evidence of *an inverse relationship between the CS estimates of IC and the share of formal long‐term care spending as a share of GDP*. These estimates suggests that although at the individual level formal and informal care are not always substitutes, at a societal level it might not be the case as formal care is not always available or afforable. In those circumstances, individuals can, only rely on IC or end up facing unmet needs. The observation that the value of caregiver support (CS) is higher in the short term than in the long term highlights that, over the course of their caregiving journey, caregivers may also experience benefits from providing care (e.g., fulfilment of a social norm), which can positively impact their overall life satisfaction.

This paper provides novel estimates of the CS per hour of informal care across several European countries. Our analysis has used data on caregiving hours from wave 8 of SHARE to estimate the CS of 1 hour of informal care in several European countries. Our estimates are in line with results from the literature,^44^ and specifically with estimates obtained using an alternative source of information (European Quality of Life Survey). We estimate the compensating surplus (CS) (PPP 2020) per hour of care in Europe to be at 9.55€/hour, ranging from 22 €/hour in Switzerland and 5 €/hour in Spain.

This study suggests that in valuing informal care from a societal perspective, it is essential to estimate the value of informal caregiving net of all of its positive effects to caregivers. This paper addresses this issue by employing wellbeing methods, which suggest that as expected, caregiving results in an overall net welfare loss for caregivers. Finally, our estimates offer valuable insights for policymakers to ensure that the value of informal care is not overlooked or underestimated.

## Conflicts of Interest

The authors declare no conflicts of interest.

## Supporting information

Supporting Information S1

## Data Availability

The data that support the findings of this study are available in SHARE at https://share‐eric.eu/. These data were derived from the following re sources available in the public domain: ‐ SHARE, https://share‐eric.eu/.

## Endnotes

^1^
Thus, in the valuation of informal care, the contingent valuation method asks individuals about their maximum willingness to pay (WTP) or the minimum willingness to accept (WTA) to provide a given or additional amount of informal care to the dependent person. Berg et al. (2005b) and de Meijer et al. (2010) argue that it is more appropriate to use WTA rather than WTP when subjects are asked to value a potential welfare loss, whereas WTP is more appropriate when subjects value a potential welfare gain.

^2^
In conjoint analysis studies, respondents are usually asked to rank or rate the scenarios presented, and in the case of discrete choice analysis, respondents are requested to choose between different scenarios.

^3^
For example, in a conjoint analysis study for the monetary valuation of informal care performed by Van den Berg et al. (2008), only 26.6% of respondents completed the questionnaire, and the response rate obtained in a discrete choice analysis study by Mentzakis, Ryan, and McNamee (2011) was only 20%.

^4^
Van den Berg and Ferrer‐i‐Carbonell (2007) found lower values for an additional hour of informal care using the well‐being method compared to willingness to accept estimates.

^5^
This change is not based on greater participation of men in caregiving tasks, but rather on structural factors (increase in female labor participation) and social factors (filial feelings of obligation to provide care).

^6^
Conde‐Sala et al. (2010) document evidence of the following ordering of caregiver burden from lowest to highest: husbands, wives, daughters, and then sons.

^7^
However, recent evidence reveals that skill formation technology follows a linear process and that childhood events may be relevant in earlier and later stages of adulthood (Case, Fertig, and Paxson 2005; Cunha, Heckman, and Schennach 2010).

^8^
These include household dysfunction (divorce, death of a parent, not living with biological parents, parental alcohol or drug use, parental illness), abuse (emotional and physical), financial stress (scarcity of resources, lack of heating), having experienced war (forced displacement, lack of food).

^9^
Low socioeconomic status during childhood is associated with economic stressors, limited social resources, physical symptoms in adulthood (Bromberger et al. 2017), insufficient medical and health resources in middle and old age (Sha, Yan, and Cheng 2018) and a higher incidence of depressive symptoms in adulthood (Mäkinen et al. 2005; Sheikh, Abelsen, and Olsen 2016).

^10^
The data have shown to be eliciting reliable information referring to the past. Havari and Mazzonna (2011) and Garrouste and Paccagnella (2011) have found that interview techniques that first start with the most immediate events and then delve into more distant domains in time lead to a small recall bias. On the other hand, combining data from administrative files does not guarantee that the result is of better quality, due to mismatch problems (Kapteyn and Ypma 2007; Meijer, Rohwedder, and Wansbeek 2012).

^11^
In contrast, Weiss (2012) used retrospective data alone (SHARELIFE) to estimate lifetime earnings, drawing on respondents' recollections of earnings throughout their entire employment history. In our study, income estimates are less affected by recollection bias as self‐reported income refers to the latest wave of SHARE.

^12^
Consistent with a detrimental overall effect of IC on LS, Figure A2 suggests a lower LS of non‐coresident female caregivers, which reveals a difference of 0.76% (Table A2), and especially large differences in certain countries: −3.60% in Italy, −5.02% in Czech Republic and −5.64% in Spain.

^13^
More specifically for each wave, SHARE provides information on individual income divided into the following categories: labor income, unemployment benefit, retirement pension, disability pension, social assistance and other benefits. This allows calculation of the current individual income expressed in PPP2020.

^14^
Brenner (2010) and Weiss (2012) also assume a constant discount rate of 2%.

^15^
Figure A4 and A5 reveal the evolution of long‐term individual income (PPP2020) over the period 2007–2020, and Figure A6 displays the distribution of long‐term income (accumulated over the period 2007–2020) by sex and caregiving status. Similar, Figures A7 and A8 show the distribution of long‐term income (2007–2020; PPP2020) by sex and caregiving status (see Appendix A for comments).

^16^
Other instruments for lagged income in the literature include lagged assets and holdings (Fields et al. 2007) and lagged expenditures (McCulloch and Baulch 2000).

^17^
(i) The number of books in the household at age 10; (ii) living with biological father/stepmother/stepfather at age 10; (iii) features of accommodation at age 10: fixed bath, cold/hot running toilet supply, inside toilet, central heating; (iii) household composition (only child, oldest child, youngest child, number of brothers, number of sisters); (iv) born in other country.

^18^
As for the reliability of personal information about past adverse events Zebrack et al. (2008) argue that subjective perceptions of physical conditions are better predictors of well‐being than objective measures of health status.

^19^
Although life satisfaction responses are ordinal in nature (on a scale from 0 to 10), in the empirical literature, linear models are usually estimated to facilitate the interpretation of the results. Ferrer‐i‐Carbonel and Frijters (2004) show that linear and ordinal models provide, essentially, the same results for life satisfaction.

^20^
The coefficients in Equation (1) can be retrieved from a panel data model with clustered or random effects. We cannot use a fixed effects model because the time invariant variables are eliminated by the demeaning operation that subtracts the mean value from all the variables in the model. However, it is susceptible to omitted variable bias. For example, individual's response to the question regarding informal care provision may be influenced by family situation, moral norms or social conditioning. The intensity of these variables is difficult to quantify and is usually controlled by individual dummy variables in a fixed effects panel model, which, as mentioned above, fails to estimate the coefficients for time invariant variables.

^21^
The FEF estimator is asymptotically consistent for a sufficiently large number of observations and a finite temporal sample, unbiased in the presence of serial correlation, and robust in arbitrary heteroscedasticity in $\varepsilon_{it}$ (Pesaran and Zhou 2018).

^22^
There is an alternative approach to estimate the causal effects of informal caregiving on life satisfaction, including the matching method (Brenna and Di Novi 2016). However, this method assumes that selection into caregiver status is based solely on observable variables included in the propensity score model. Although this assumption cannot be tested, it is expected to hold if all relevant variables are observable. In our case, it is plausible that if the parents' or spouse's health worsens, the probability of becoming an informal caregiver increase. But the availability of formal care (e.g., nursing homes), may increase the use of formal care, thus reducing the probability of informal caregiving. In SHARE, we do not have information on people who have entered residential care on a permanent basis; they disappear from the sample and cannot be followed up. For this reason, we consider that the use of this method is not entirely reliable in addressing the potential endogeneity of the decision to become a caregiver.

^23^
While some research suggests that the length of time spent caregiving is associated with greater demands (Liu et al. 2022; Rahmani et al. 2022), other studies suggest no difference in caregiver outcomes as a function of the amount of time spent caregiving (Morimoto, Schreiner, and Asano 2003; Gaugler 2010; Jeong et al. 2020). Evidence of an inverse relationship between caregiving burden and length of caregiving has also been documented (Zainuddin, Arokiasamy, and Poi 2003; Gbiri, Olawale, and Isaac 2015; Hidru et al. 2016).

^24^
In the first‐step regression for long‐term individual income (2007–2020; PPP2020), long‐term income from partner (2007–2020; PPP2020) and long‐term income from other household members (2007–2020; PPP2020), also in logarithms, are used.

^25^
In the case of coresident caregivers, the probability of being a caregiver increases 20.91pp for the only child (11.65pp for men and 30.32pp for women) and 20.47pp for the oldest child (12.72pp for men and 35.26pp for women).

^26^
Conventional statistical tests suggest that the instruments are relevant. Anderson canonical correlations likelihood‐ratio tests reject the null of under identification, and none of the Hansen's J‐statistics reject the null that the instruments are satisfying the orthogonality condition.

^27^
Consistently, the effect of long‐term income is greater than that of short‐term income. Having experienced past periods of stress (13.23pp), financial strain (16.51pp) or poor health (15.25pp) is associated with higher life satisfaction in the present.

^28^
Other variables that influence life satisfaction include having lived with a biological mother, having central heating, or having hot water in the home (during childhood).

^29^
Empirical evidence on the effects of adverse childhood events seems to indicate that in some cases (e.g., low socioeconomic status) there is an increase in life satisfaction (which indirectly acts to decrease the incidence of depression) (Tang et al. 2022), while in other cases, there is a lasting impairment in life satisfaction (i.e., maltreatment) (Pierce, Abbey, and Wegner 2018).

^30^
This difference is the highest in Belgium (€40,583) and lowest in Italy (€7578). The CS for men and women amounts to €17,362 and €11,739, respectively, representing a difference of €5263. This difference is the highest in Spain (€11,062) and the lowest in Denmark (€3290).

^31^
With respect to the percentage of individual CS short‐term with respect to GDP per capita (PPP2020), the maximum corresponds to Spain (52.35%) and the minimum to Sweden (12.43%). We highlight some cases in which this percentage is above 100%: male co‐residents in all countries except Italy and Switzerland; male non‐coresidents in Spain; female co‐residents in Belgium, Denmark and France.

^32^
In some cases, this percentage is higher than 200% (co‐resident curators in France, both men and women, and co‐resident men in the Czech Republic).

^33^
Furthermore, the role of a caregiver can lead to the discovery of latent skills, social norm fulfillment as well as a bond with the care recipient, all of which can positively impact life satisfaction (Lin, Fee, and Wu 2012; Wang et al. 2018).

^34^
However, such a comparison may be worthwhile to detect countries exhibiting both high LTC expenditure and high CS.

^35^
This classification does not include Belgium and Switzerland. In order to include them we have relied on other works. Belgium and Switzerland are included in standard care mix model following Jiménez‐Martín and Vilaplana‐Prieto (2015) and Ilinca et al. (2022), respectively.

^36^
It is important to note that our analysis lacks information on the institutionalised population, as SHARE does not interview people living in nursing homes. This fact may explain the correlation between long‐term care expenditure and the value of informal care (proxied by the CS). Indeed, in countries with higher long‐term care expenditures, more people can access a place in a nursing home. Conversely, in countries with lower long‐term care expenditure, more people in need have to continue to live at home, receiving informal care.

^37^
Previous studies refer to informal caregivers with regards to dementia (König and Wettstein 2002); cerebrovascular accident (Oliva‐Moreno et al. 2013); Alzheimer's disease (Peña‐Longobardo and Oliva‐Moreno 2015); myocardial infarction (Rabier et al. 2020)).

^38^
However, when it comes to coresident caregivers, Spain is one of the countries with the highest CS per hour (27 €/hour).

^39^
Agriculture, hunting, forestry, fishing; Mining and quarrying; Manufacturing; Electricity, gas and water supply; Construction; Wholesale and retail trade; repair of motor vehicles, motorcycles and personal and household goods; Hotels and restaurants; Transport, storage and communication; Financial intermediation; Real estate, renting and business activities; Public administration and defense; compulsory social security; Education; Health and social work; Other community, social and personal service activities.

^40^
The European Union has created a common nomenclature of territorial statistical units, called “NUTS”, to enable the collection, generation and dissemination of harmonized regional statistics in the Union. This hierarchical system is also used in socio‐economic analyses of the regions and in the formulation of interventions in the context of the Union's cohesion policy. The NUTS nomenclature is hierarchical in that it subdivides each Member State into three levels: NUTS 1, NUTS 2 and NUTS 3. The second and third levels are subdivisions of the first and second levels respectively. Each Member State may establish further levels of hierarchical detail by subdividing NUTS level 3. The current NUTS nomenclature distinguishes 242 NUTS level 2 regions.

^41^
Previous research has yielded inconclusive findings in this regard. While the number of siblings has been linked to improved lung function in children (Mattes et al. 1999), children with older siblings are also at higher risk of contracting whooping cough and experiencing delayed vaccination (Reading, Surridge, and Adamson 2004). Moreover, family dynamics, such as favoritism or imbalances in caregiving, can influence children's development (Adler 1998). However, studies like that of Khodarahimi and Ogletree (2011) have noted a significant correlation between larger family size and lower life satisfaction. Additionally, research indicates that children with siblings may be less likely to trust their parents (Hesketh, Qu, and Tomkins 2003).

^42^
However, it is important to bear in mind that the estimates of Peña‐Longobardo and Oliva‐Moreno 2022 refer to all caregivers, while our CS is only calculated for caregivers aged over the age of 50 alone. These results suggest some level of consistency between the two methods of valuing informal caregiving.

^43^
Our estimates refer to care provided in the community, although a limitation of our approach is that our dataset does not contain information of on caregiver in residential settings. Furthermore, our estimates on the cost per hour are retrieved form only one wave (wave 8) of the survey where the information was available.

^44^
These estimates offer detailed insights and align with previous findings in the literature. Schneider and Kleindienst (2016) estimated the value of an hour of informal care across various countries (Austria, Belgium, the Czech Republic, Denmark, France, Germany, Italy, the Netherlands, Poland, Spain, Sweden, and Switzerland). Their analysis, based on data from earlier waves of SHARE where hours were censored by intervals, yielded less precise results, suggesting that providing less than 10 h of care per week has a value of €2.54/hour. For specific conditions, the values range from 12.71 €/hour for stroke (Oliva‐Moreno et al. 2013) to 18.68 €/hour for Alzheimer's disease (Peña‐Longobardo and Oliva‐Moreno 2015), and 10.9 €/hour for myocardial infarction (Rabier et al. 2020).
