# Supplementary material for: The Hidden Value of Adult Informal Care in Europe
Source: Health Econ. 2025 Jan 29;34(4):791–812. doi: 10.1002/hec.4928 (PMC11890091; doi:10.1002/hec.4928)
Supplement: Supplementary file 1 — Supporting Information S1 [file HEC-34-791-s001.docx]

**Appendix for Online Publication**

**Appendix A**

**Figure A1. Density function for life satisfaction by sex and caregiving status.**

|  |
| --- |

Source: own work using SHARE (waves 2, 4, 5,6, 7 and 8).

Notes: Blue straight line represents the density function of life satisfaction for men who are not informal caregivers and do not receive care.Blue dashed line represents the density function of life satisfaction for men who are informal caregivers.

Red straight line represents the density function of life satisfaction for women who are not informal caregivers and do not receive care. Red dashed line represents the density function of life satisfaction for women who are informal caregivers.

**Figure A2. Density function for life satisfaction by sex and type of caregiver**

| **** |
| --- |

Source: own work using SHARE (waves 2, 4, 5,6, 7 and 8).

Notes: Blue straight line represents the density function of life satisfaction for men who are non-coresident caregivers

Blue dashed line represents the density function of life satisfaction for men who are coresident caregivers.

Red straight line represents the density function of life satisfaction for women who are non-coresident caregivers

Red dashed line represents the density function of life satisfaction for women who are coresident caregivers.

**Figure A3. Distribution of the sample by wave.**

|  |
| --- |
|  |

Source: own work using SHARE (waves 2, 4, 5,6, 7 and 8).

Notes: Each bar represents the percentage distribution of the sample by wave and sex in the following groups: “not caregiver and not receive care”, “receive care”, “coresident informal caregiver (but not non-coresident caregiver)”, “non-coresident informal caregiver (but not coresident caregiver)” and “coresident and non-coresident informal caregiver”. We observe that: (i) as expected the percentage of non-coresident caregivers (and non-coresident caregivers) decreases over time as we follow the same individuals in the period 2007-2020; (ii) similarly, the percentage receiving care increases (from 5.53% to 16.32% for men and from 9.51% to 21.89% for women); (iii) the percentage of coresident caregivers increases (from 2.73% to 5.36% for men and from 9.51% to 21.89% for women); (iv) the percentage of coresident caregivers increases (from 2.73% to 5.36% for men and from 3.8% to 5.68% for women); (iv) by the contrary, the percentage of non-coresident caregivers decreases (from 37.16% to 26.78% for men and from 37.35% to 23.87% for women); (v) the percentage of coresidents & non-coresident caregivers remains stable (below 2%) for men and decreases from 2.58% to 1.70% for women

**Figure A4. Annual income (reported and imputed) by sex and caregiving status (PPP2020).**

|  |
| --- |

Source: own work using SHARE (waves 2, 4, 5, 6, 7, and 8). Total current income includes earnings, unemployment benefits, retirement benefits, disability benefits, other benefits and social assistance.

Note: Blue straight line represents individual current income (PPP2020) for men who are not caregivers and do not receive any type of care. Blue dashed line represents individual current income (PPP2020) for men who are caregivers.

Red straight line represents individual current income (PPP2020) for women who are not caregivers and do not receive any type of care. Red dashed line represents individual current income (PPP2020) for women who are caregivers.

**Figure A5. Annual income (reported and imputed) by sex and type of caregiver (PPP2020).**

|  |
| --- |

Source: own work using SHARE (waves 2, 4, 5, 6, 7, and 8). Total current income includes earnings, unemployment benefits, retirement benefits, disability benefits, other benefits and social assistance.

Note: Blue straight line represents individual current income (PPP2020) for men who are non-coresident caregivers.

Blue dashed line represents individual current income (PPP2020) for men who are coresident caregivers.

Red straight line represents individual current income (PPP2020) for women who are non-coresident caregivers.

Red dashed line represents individual current income (PPP2020) for women who are coresident caregivers. We observe that: (i) long-term income increases until 2011 and decreases thereafter, which is consistent with the fact that in 2007, 40% of the employed were between 56-64 years old in 2007, so that after four years, there is a high percentage reaching the normal retirement age or opting for early retirement; (ii) the income of male and female non-caregivers is higher than the income of male and female caregivers; (iii) the income of males (both caregivers and non-caregivers) is higher than the income of females (both caregivers and non-caregivers); (iv) the income of non-coresident caregivers (both males and females) is higher than the income of co-resident caregivers (both males and females).

**Figure A6. Composition of long-term income by caregiving status and sex (%)**

|  |
| --- |
|  |

Each bar shows the percentage distribution of long-term income 2007-2020 expressed in PPP2020 and accumulated to 2020 with a discount rate of 2%.

Source: own work using SHARE (waves 2, 4, 5,6, 7, and 8)

Note: We display the percentage represented by labor earnings for coresident caregivers (21.21% for men and 23.26% for women) is lower than that of non-caregivers (30.13% and 27.57%, respectively). In contrast, the percentage represented by retirement benefits (73.45% for men and 69.50% for women) is higher than for non-caregivers (63.98% and 65.92%, respectively).

**Figure A7. Density function for long-term income (2004-2020; PPP2020) by sex and caregiving status.**

|  |
| --- |

Source: own work using SHARE (waves 2, 4, 5,6, 7 and 8).

Note: Blue straight line represents individual long-term income (2007-2020; PPP2020) for men who are not caregivers and do not receive care. Blue dashed line represents individual long-term income (2007-2020; PPP2020) for men who are caregivers

Red straight line represents individual long-term income (2007-2020; PPP2020) for women who are not caregivers and do not receive care. Red dashed line represents individual long-term income (2007-2020; PPP2020) for women who are caregivers.

**Figure A8. Density function for long-term income (2004-2020; PPP2020) by sex and type of caregiver**

|  |
| --- |

Source: own work using SHARE (waves 2, 4, 5,6, 7 and 8).

Note: Blue straight line represents individual long-term income (2007-2020; PPP2020) for men who are non-coresident caregivers.

Blue dashed line represents individual long-term income (2007-2020; PPP2020) for men who are coresident caregivers.

Red straight line represents individual long-term income (2007-2020; PPP2020) for women who are non-coresident caregivers.

Red dashed line represents individual long-term income (2007-2020; PPP2020) for women who are coresident caregivers.

We display: (i) the long-term individual income of men is higher than that of women; (ii) the long-term income of male caregivers is higher than that of female non-caregivers (who do not receive care); (iii) the long-term income of non-coresident caregivers is higher than that of coresident caregivers; (iv) the density function of the long-term income of female coresident caregivers is considerably more shifted to the left with respect to the density function of male coresident caregivers.

**Table A1. Description of the sample**

|  | # individuals | # observations | | | | | | |
| --- | --- | --- | --- | --- | --- | --- | --- | --- |
|  |  | Total | No informal caregivers | | | Informal caregivers | | |
|  |  |  | Total | No receive care | Receive care | Total | Coresident IC | Non-coresid. IC |
| Austria | 278 | 1,668 | 1,149 | 881 | 268 | 519 | 97 | 449 |
| Belgium | 1,115 | 6,690 | 4,453 | 3,644 | 809 | 2,237 | 342 | 2,001 |
| Czech Republic | 668 | 4,008 | 2,734 | 2,097 | 637 | 1,274 | 281 | 1,106 |
| Denmark | 952 | 5,712 | 3,086 | 2,496 | 590 | 2,626 | 246 | 2,487 |
| France | 655 | 3,930 | 2,561 | 2,119 | 442 | 1,369 | 246 | 1,205 |
| Germany | 628 | 3,768 | 2,413 | 1,917 | 496 | 1,355 | 241 | 1,192 |
| Italy | 1,072 | 6,432 | 4,824 | 4,189 | 635 | 1,608 | 606 | 1,120 |
| Spain | 804 | 4,824 | 3,987 | 3,401 | 586 | 837 | 465 | 425 |
| Sweden | 596 | 3,576 | 2,253 | 1,883 | 370 | 1,323 | 124 | 1,252 |
| Switzerland | 600 | 3,600 | 2,419 | 2,076 | 343 | 1,181 | 115 | 1,108 |
| Total | 7.368 | 44.208 | 29.879 | 24.703 | 5.176 | 14.329 | 2.763 | 12.345 |
| % |  |  | 67.59 | 55.88 | 11.71 | 32.41 | 6.25 | 27.92 |

Source: Own work using SHARE (waves 2, 4, 5, 6, 7 and 8). Netherlands was not included in wave 6.

**Table A2. Life satisfaction by country, sex and caregiving status**

|  | No informal caregiver | No informal caregiver & no receive care | No informal caregiver & receive care | Informal caregiver |
| --- | --- | --- | --- | --- |
| **Total** |  |  |  |  |
| Austria | 8.038 | 8.318 | 7.530 | 8.218 |
| Belgium | 7.896 | 7.970 | 7.617 | 7.876 |
| Czech Republic | 7.617 | 7.822 | 6.976 | 7.429 |
| Denmark | 8.573 | 8.670 | 8.234 | 8.660 |
| France | 7.634 | 7.711 | 7.278 | 7.584 |
| Germany | 7.938 | 8.017 | 7.645 | 7.865 |
| Italy | 7.601 | 7.697 | 7.020 | 7.420 |
| Spain | 7.691 | 7.831 | 6.949 | 7.389 |
| Sweden | 8.493 | 8.602 | 7.959 | 8.446 |
| Switzerland | 8.455 | 8.495 | 8.219 | 8.420 |
| Total | 7.945 | 8.048 | 7.493 | 7.987 |
| **Men** |  |  |  |  |
| Austria | 8.165 | 8.299 | 7.631 | 8.289 |
| Belgium | 7.979 | 8.070 | 7.718 | 8.036 |
| Czech Republic | 7.783 | 7.886 | 7.291 | 7.716 |
| Denmark | 8.580 | 8.654 | 8.202 | 8.647 |
| France | 7.819 | 7.850 | 7.593 | 7.746 |
| Germany | 7.880 | 7.966 | 7.503 | 7.862 |
| Italy | 7.748 | 7.811 | 7.231 | 7.586 |
| Spain | 7.936 | 8.022 | 7.208 | 7.719 |
| Sweden | 8.534 | 8.600 | 8.102 | 8.513 |
| Switzerland | 8.498 | 8.564 | 7.907 | 8.559 |
| Total | 8.045 | 8.116 | 7.610 | 8.130 |
| **Women** |  |  |  |  |
| Austria | 7.973 | 8.331 | 7.498 | 8.176 |
| Belgium | 7.830 | 7.913 | 7.554 | 7.726 |
| Czech Republic | 7.509 | 7.774 | 6.854 | 7.273 |
| Denmark | 8.569 | 8.683 | 8.248 | 8.671 |
| France | 7.501 | 7.598 | 7.157 | 7.472 |
| Germany | 7.987 | 8.064 | 7.741 | 7.867 |
| Italy | 7.480 | 7.597 | 6.912 | 7.328 |
| Spain | 7.507 | 7.671 | 6.844 | 7.188 |
| Sweden | 8.465 | 8.604 | 7.893 | 8.397 |
| Switzerland | 8.423 | 8.441 | 8.341 | 8.330 |
| Total | 7.870 | 7.992 | 7.438 | 7.886 |

Source: Own work using SHARE (waves 2, 4, 5, 6, 7 and 8)

**Table A3. Percentage of informal caregivers by co-residence, sex and country**

|  | N | Percentage with respect to total population | | |
| --- | --- | --- | --- | --- |
|  |  | Coresident caregiver | Non-coresident caregiver | Total |
| **Total** |  |  |  |  |
| Austria | 519 | 5.08 | 27.50 | 32.58 |
| Belgium | 2,237 | 5.02 | 32.51 | 37.53 |
| Czech Rep. | 1,274 | 5.66 | 22.81 | 28.47 |
| Denmark | 2,626 | 4.10 | 40.75 | 44.84 |
| France | 1,369 | 5.61 | 30.18 | 35.79 |
| Germany | 1,355 | 5.44 | 30.76 | 36.20 |
| Italy | 1,608 | 8.65 | 18.58 | 27.23 |
| Spain | 837 | 8.82 | 9.77 | 18.59 |
| Sweden | 1,323 | 3.03 | 33.40 | 36.43 |
| Switzerland | 1,181 | 2.58 | 29.36 | 31.94 |
| All | 14,329 | 5.66 | 27.26 | 32.92 |
| **Men** |  |  |  |  |
| Austria | 211 | 5.91 | 25.41 | 31.31 |
| Belgium | 973 | 4.91 | 32.90 | 37.81 |
| Czech Rep. | 447 | 6.65 | 24.02 | 30.67 |
| Denmark | 1,180 | 4.43 | 39.44 | 43.87 |
| France | 562 | 5.29 | 31.80 | 37.09 |
| Germany | 609 | 5.95 | 32.15 | 38.10 |
| Italy | 572 | 10.11 | 21.24 | 31.35 |
| Spain | 317 | 8.93 | 11.75 | 20.69 |
| Sweden | 559 | 3.36 | 31.98 | 35.34 |
| Switzerland | 463 | 3.15 | 29.59 | 32.75 |
| All | 5,893 | 5.07 | 26.44 | 31.51 |
| **Women** |  |  |  |  |
| Austria | 308 | 3.76 | 30.82 | 34.59 |
| Belgium | 1,264 | 5.15 | 32.05 | 37.20 |
| Czech Rep. | 827 | 4.27 | 21.10 | 25.38 |
| Denmark | 1,446 | 3.69 | 42.31 | 46.01 |
| France | 807 | 6.02 | 28.12 | 34.14 |
| Germany | 746 | 4.86 | 29.14 | 34.00 |
| Italy | 1,036 | 6.79 | 15.18 | 21.97 |
| Spain | 520 | 8.67 | 7.30 | 15.97 |
| Sweden | 764 | 2.60 | 35.23 | 37.84 |
| Switzerland | 718 | 1.83 | 29.05 | 30.87 |
| All | 8,436 | 6.13 | 27.91 | 34.04 |

Source: Own work using SHARE (waves 2, 4, 5, 6, 7 and 8)

**Table A4. Descriptive statistics**

|  | Men | | | | Women | | | |
| --- | --- | --- | --- | --- | --- | --- | --- | --- |
|  | Total | No caregiver  No receive care | Receive care | Caregiver | Total | No caregiver  No receive care | Receive care | Caregiver |
| N | 18,648 | 11,098 | 1,657 | 5,893 | 25,560 | 13,605 | 3,519 | 8,436 |
| **Time invariant characteristics (past information from SHARELIFE)** |  |  |  |  |  |  |  |  |
| Period during which he/she felt happier than during the rest of his/her life (%) | 40.99 | 40.77 | 42.00 | 41.12 | 52.25 | 50.72 | 54.05 | 53.97 |
| Age when happiness period started | 28.09 | 28.31 | 27.99 | 27.71 | 26.38 | 26.33 | 26.04 | 26.59 |
|  | (13.69) | (13.66) | (13.71) | (13.72) | (12.67) | (12.48) | (13.30) | (12.69) |
| Length of happiness period (years) | 22.13 | 22.26 | 24.05 | 21.34 | 22.34 | 22.54 | 25.63 | 20.67 |
|  | (17.12) | 17.24) | (18.05) | (16.56) | (16.93) | (16.94) | (18.88) | (15.80) |
| Period during which he/she felt under more stress than during the rest of his/her life (%) | 50.80 | 48.04 | 50.94 | 55.96 | 59.79 | 56.41 | 57.63 | 66.13 |
| Age when stress period started | 41.74 | 41.95 | 42.26 | 41.27 | 41.67 | 41.53 | 42.74 | 41.48 |
|  | (12.97) | (13.00) | (14.06) | (12.61) | (14.03) | (14.00) | (16.43) | (13.09) |
| Length of stress period (years) | 7.90 | 7.85 | 9.78 | 7.50 | 8.40 | 8.31 | 10.14 | 7.89 |
|  | (8.86) | (8.89) | (10.15) | (8.39) | (9.39) | (9.37) | (10.69) | (8.83) |
| Period during which health was poorer compared to the rest of his/her your life (%) | 38.35 | 36.57 | 48.46 | 38.88 | 44.65 | 41.35 | 53.48 | 46.28 |
| Age when poor health period started | 48.40 | 49.33 | 51.43 | 45.67 | 46.25 | 46.17 | 49.87 | 44.63 |
|  | (16.26) | (15.77) | (16.26) | (16.73) | (15.67) | (15.45) | (16.98) | (15.03) |
| Length of poor health period (years) | 6.20 | 6.15 | 7.73 | 5.76 | 7.83 | 7.58 | 9.70 | 7.28 |
|  | (7.91) | (7.85) | (8.73) | (7.64) | (9.61) | (9.41) | (10.95) | (9.07) |
| Period during which there was a distinct financial hardship (%) | 31.37 | 30.12 | 33.80 | 33.04 | 35.31 | 32.11 | 38.59 | 39.08 |
| Age when financial hardship started | 34.46 | 34.50 | 35.64 | 34.04 | 32.86 | 32.74 | 32.57 | 33.14 |
|  | (13.88) | (14.08) | (15.47) | (13.02) | (13.80) | (13.81) | (15.74) | (12.90) |
| Length of financial hardship period (years) | 8.17 | 8.57 | 8.82 | 7.29 | 9.33 | 9.10 | 11.72 | 8.68 |
|  | (10.02) | (10.71) | (11.78) | (7.99) | (10.59) | (10.32) | (12.93) | (9.73) |
| Period during which suffered from hunger (%) | 6.92 | 6.77 | 11.71 | 5.85 | 6.17 | 5.70 | 10.49 | 5.14 |
| Age when hunger period period started | 8.08 | 7.46 | 8.72 | 9.09 | 10.88 | 9.81 | 11.34 | 12.40 |
|  | 7.03) | 6.61) | 6.67) | 7.95) | 11.98) | 11.22) | 11.75) | 13.25) |
| Length of hunger period (years) | 5.48 | 5.81 | 5.04 | 4.99 | 5.64 | 5.64 | 5.82 | 5.49 |
|  | (4.54) | (4.64) | (4.00) | (4.55) | (4.80) | (4.75) | (4.61) | (5.06) |
| Ever been the victim of such persecution or discrimination | 5.41 | 5.04 | 6.34 | 5.84 | 4.60 | 3.79 | 6.28 | 5.20 |
| Health during childhood |  |  |  |  |  |  |  |  |
| Excellent | 38.16 | 37.13 | 36.93 | 40.44 | 33.31 | 32.61 | 33.13 | 34.51 |
| Very good | 32.88 | 33.61 | 32.77 | 31.55 | 33.03 | 33.68 | 30.18 | 33.17 |
| Good | 20.53 | 21.06 | 20.70 | 19.48 | 23.76 | 24.21 | 24.84 | 22.57 |
| Fair | 5.50 | 5.49 | 6.16 | 5.35 | 6.62 | 6.50 | 8.47 | 6.05 |
| Poor | 2.28 | 2.01 | 3.08 | 2.58 | 2.68 | 2.42 | 2.56 | 3.14 |
| Health varied a great deal | 0.55 | 0.59 | 0.30 | 0.53 | 0.54 | 0.45 | 0.82 | 0.57 |
| During your childhood. ever in hospital for >=1 month | 6.27 | 5.61 | 6.76 | 7.38 | 6.34 | 5.40 | 7.76 | 7.25 |
| A physical injury that has led to any permanent handicap | 15.25 | 14.31 | 23.05 | 14.83 | 12.46 | 11.30 | 17.79 | 12.11 |
| Books in the place where lived at age 10 |  |  |  |  |  |  |  |  |
| None or very few (0-10 books) | 39.38 | 42.67 | 44.96 | 31.61 | 37.51 | 39.95 | 43.65 | 31.02 |
| Enough to fill one shelf (11-25 books) | 20.40 | 20.46 | 18.53 | 20.80 | 20.80 | 20.98 | 20.60 | 20.59 |
| Enough to fill one bookcase (26-100 books) | 23.58 | 21.50 | 21.85 | 28.00 | 24.32 | 23.42 | 20.74 | 27.26 |
| Enough to fill two bookcases (101-200 books) | 7.88 | 7.29 | 6.76 | 9.32 | 8.54 | 7.53 | 7.39 | 10.66 |
| Enough to fill two or more bookcases (+ 200 books) | 8,75 | 8,07 | 7,91 | 10,27 | 8,83 | 8,12 | 7,62 | 10,47 |
| Performance in Maths at age 10 compared to other children |  |  |  |  |  |  |  |  |
| Much better | 15,06 | 14,16 | 13,52 | 17,19 | 10,47 | 9,61 | 9,52 | 12,25 |
| Better | 28.44 | 27.98 | 26.43 | 29.88 | 23.47 | 23.08 | 21.51 | 24.93 |
| About the same | 43.40 | 43.78 | 43.27 | 42.73 | 48.99 | 49.76 | 48.31 | 48.03 |
| Worse | 8.85 | 9.49 | 9.96 | 7.33 | 11.69 | 11.88 | 12.22 | 11.17 |
| Much worse | 1.80 | 1.77 | 2.41 | 1.70 | 2.54 | 2.64 | 2.81 | 2.25 |
| Did not go to school | 2.45 | 2.83 | 4.41 | 1.17 | 2.84 | 3.03 | 5.63 | 1.38 |
| Performance in Language at age 10 compared to other children |  |  |  |  |  |  |  |  |
| Much better | 10.14 | 9.45 | 10.74 | 11.25 | 13.40 | 12.30 | 11.51 | 15.98 |
| Better | 23.91 | 22.63 | 24.44 | 26.17 | 29.48 | 28.40 | 28.05 | 31.83 |
| About the same | 46.07 | 47.43 | 41.64 | 44.76 | 43.90 | 45.55 | 43.56 | 41.37 |
| Worse | 14.67 | 15.03 | 16.11 | 13.59 | 8.57 | 9.06 | 9.04 | 7.59 |
| Much worse | 2.16 | 2.08 | 2.23 | 2.27 | 1.27 | 1.12 | 1.62 | 1.36 |
| Did not go to school | 3,06 | 3,38 | 4,83 | 1,95 | 3,38 | 3,58 | 6,22 | 1,87 |
| At age 10 lived with |  |  |  |  |  |  |  |  |
| Biological father | 89.54 | 89.75 | 88.11 | 89.55 | 88.85 | 89.14 | 86.84 | 89.22 |
| Biological mother | 94.72 | 94.53 | 94.68 | 95.35 | 94.01 | 94.20 | 94.68 | 94.29 |
| Stepfather | 1.64 | 1.68 | 1.87 | 1.49 | 1.95 | 1.77 | 2.59 | 1.97 |
| Stepmother | 2.28 | 2.29 | 1.75 | 2.43 | 2.49 | 2.21 | 2.76 | 2.82 |
| Features of accommodation when age 10 | 37.55 | 35.45 | 30.78 | 43.41 | 38.97 | 38.57 | 28.76 | 43.86 |
| Fixed bath |  |  |  |  |  |  |  |  |
| Cold running water supply | 72.43 | 70.36 | 62.76 | 79.03 | 72.35 | 71.36 | 62.12 | 78.21 |
| Hold running water supply | 36.55 | 34.14 | 26.98 | 43.78 | 38.12 | 37.28 | 25.97 | 44.55 |
| Inside toilet | 53.89 | 52.64 | 44.72 | 58.83 | 55.12 | 54.51 | 45.27 | 60.21 |
| Central heating | 23.20 | 20.60 | 17.14 | 29.80 | 23.59 | 21.81 | 16.65 | 29.36 |
| Ever experienced any of these events |  |  |  |  |  |  |  |  |
| Lived in a children’s home | 1.54 | 1.46 | 2.47 | 1.44 | 2.02 | 2.01 | 2.27 | 1.93 |
| Been fostered with another family | 1.71 | 1.51 | 1.93 | 2.00 | 1.88 | 1.65 | 2.39 | 2.03 |
| Evacuated or relocated during a war | 3.86 | 3.85 | 5.91 | 3.31 | 3.38 | 2.96 | 5.71 | 3.08 |
| Lived in a prisoner of war camp | 0.29 | 0.25 | 0.78 | 0.22 | 0.09 | 0.10 | 0.06 | 0.09 |
| Lived in a prison | 0.58 | 0.48 | 1.03 | 0.64 | 0.12 | 0.10 | 0.09 | 0.17 |
| Lived in a labor camp | 0.32 | 0.33 | 0.42 | 0.27 | 0.26 | 0.31 | 0.28 | 0.17 |
| Lived in a concentration camp | 0.00 | 0.00 | 0.00 | 0.00 | 0.14 | 0.11 | 0.00 | 0.17 |
| Stayed in a psychiatric hospital | 0.61 | 0.59 | 0.60 | 0.64 | 0.63 | 0.57 | 0.94 | 0.62 |
| Been an inpatient in a tuberculosis institution | 0.42 | 0.35 | 0.60 | 0.49 | 0.63 | 0.68 | 0.80 | 0.49 |
| Been homeless for 1 month or more | 0.26 | 0.27 | 0.36 | 0.20 | 0.23 | 0.20 | 0.23 | 0.30 |
| **Other time invariant characteristics (regular SHARE waves)** |  |  |  |  |  |  |  |  |
| Level of education | 0.83 | 0.84 | 0.76 | 0.82 | 0.87 | 0.85 | 0.76 | 0.90 |
| Pre-primary education and primary educ. | 21.41 | 23.22 | 30.30 | 15.51 | 27.22 | 27.93 | 39.70 | 20.87 |
| Lower secondary education | 16.42 | 17.45 | 16.23 | 14.53 | 16.94 | 16.69 | 17.76 | 17.01 |
| Upper secondary education | 28.42 | 25.38 | 26.98 | 34.55 | 27.64 | 26.25 | 24.98 | 30.99 |
| Postsecondary nontertiary education | 3.91 | 3.61 | 3.02 | 4.73 | 3.52 | 3.23 | 2.47 | 4.42 |
| First stage of tertiary education | 24.51 | 21.73 | 23.05 | 30.15 | 20.17 | 17.69 | 14.89 | 26.36 |
| Second stage of tertiary education | 0.65 | 0.75 | 0.42 | 0.53 | 0.25 | 0.20 | 0.20 | 0.34 |
| Family characteristics |  |  |  |  |  |  |  |  |
| Single child | 2.33 | 1.98 | 2.72 | 2.88 | 1.96 | 1.84 | 2.70 | 1.85 |
| Oldest child | 2.02 | 1.88 | 1.03 | 2.56 | 1.95 | 1.76 | 1.05 | 2.64 |
| Youngest child | 1.73 | 1.68 | 1.09 | 1.99 | 1.89 | 1.72 | 1.02 | 2.52 |
| Has brothers | 50.91 | 49.73 | 46.05 | 54.51 | 53.00 | 51.38 | 47.54 | 57.91 |
| Has sisters | 54.52 | 53.10 | 54.38 | 57.22 | 55.40 | 53.67 | 53.03 | 59.16 |
| Number of brothers | 1.77 | 1.78 | 1.65 | 1.77 | 1.67 | 1.69 | 1.63 | 1.66 |
|  | (1.14) | (1.13) | (1.00) | (1.17) | (1.06) | (1.08) | (1.06) | (1.01) |
| Number of sisters | 1.72 | 1.74 | 1.68 | 1.69 | 1.79 | 1.80 | 1.79 | 1.78 |
|  | (1.07) | (1.12) | (1.04) | (1.00) | 1.14) | (1.14) | (1.18) | (1.13) |
| Born in other country | 12.58 | 12.71 | 11.65 | 12.71 | 13.90 | 13.70 | 11.65 | 14.44 |
| Long-term individual income (PPP2020) | 324,453 | 313,704 | 308,030 | 352,402 | 200,900 | 187,068 | 308,030 | 222,275 |
|  | (268,332) | 2(63,082) | (239,410) | (281,127) | (174,362) | (172,541) | (239,410) | (172,684) |
| Earnings | 99,580 | 94,012 | 78,607 | 124,573 | 55,889 | 51,428 | 78,607 | 76,389 |
|  | (170,816) | (170,070) | (155,324) | (180,258) | (108,528) | (102,434) | (155,324) | (125,026) |
| Unemployment benefits | 3,383 | 3,401 | 2,643 | 3,625 | 3,154 | 2,929 | 2,643 | 3,837 |
|  | (22,978) | (23,265) | (18,755) | (22,465) | (30,814) | (29,679) | (18,755) | (34,191) |
| Social assistance | 678 | 639 | 1,157 | 484 | 833 | 513 | 1,157 | 863 |
|  | (9,743) | (11,799) | (5,617) | (5,064) | (11,266) | (6,851) | (5,617) | (16,121) |
| Retirement benefits | 205,525 | 201,280 | 208,202 | 207,853 | 131,103 | 123,726 | 208,202 | 129,979 |
|  | (232,744) | (222,960) | (220,668) | (249,055) | (150,850) | (150,447) | (220,668) | (142,373) |
| Other benefits | 7,768 | 7,087 | 6,148 | 9,619 | 4,186 | 3,669 | 6,148 | 4,936 |
|  | (52,190) | (48,756) | (24,046) | (63,174) | (24,376) | (21,370) | (24,046) | (27,702) |
| Disability benefits | 7,519 | 7,284 | 11,273 | 6,248 | 5,735 | 4,803 | 11,273 | 6,271 |
|  | (42,642) | (44,072) | 48,248) | (32,713) | (31,173) | (27,586) | (48,248) | (35,548) |
| Long-term income from partner (2007-2020; PPP2020) | 269,043 | 258,047 | 235,585 | 296,103 | 312,955 | 349,090 | 323,342 | 270,984 |
|  | (264,189) | (234,644) | (157,221) | (310,785) | (277,910) | (281,547) | (135,190) | (302,988) |
| Long-term income from other household members (2007-2020; PPP2020) | 118,560 | 121,132 | 117,793 | 117,331 | 119,137 | 119,488 | 119,635 | 122,711 |
|  | (57,234) | (65,797) | (35,159) | (49,183) | (88,678) | (69,052) | 38,729) | (118,002) |
| **Time varying characteristics** |  |  |  |  |  |  |  |  |
| Age (years) | 69.72 | 69.65 | 75.34 | 68.26 | 69.17 | 68.87 | 75.90 | 66.82 |
|  | (8.70) | (8.46) | (9.41) | (8.26) | (9.42) | (9.02) | (9.86) | (8.45) |
| 50-59 | 11.40 | 10.87 | 4.83 | 14.25 | 14.67 | 14.23 | 5.29 | 19.30 |
| 60-69 | 37.55 | 36.02 | 23.17 | 44.48 | 37.00 | 35.76 | 21.88 | 45.31 |
| 70-79 | 32.57 | 32.92 | 35.00 | 31.24 | 29.82 | 30.30 | 33.42 | 27.56 |
| 80 and + | 18.47 | 20.19 | 36.99 | 10.03 | 18.51 | 19.71 | 39.41 | 7.84 |
| Marital status |  |  |  |  |  |  |  |  |
| Married/cohabiting | 77.35 | 77.12 | 68.20 | 80.35 | 61.42 | 65.28 | 39.81 | 64.21 |
| Single | 5.47 | 4.76 | 8.87 | 5.85 | 4.66 | 3.96 | 5.06 | 5.61 |
| Separated/divorced | 6.26 | 5.40 | 6.34 | 7.86 | 9.50 | 7.34 | 10.77 | 12.45 |
| Widow | 6.25 | 4.87 | 16.60 | 5.94 | 20.16 | 15.41 | 44.36 | 17.73 |
| Household size | 2.14 | 2.18 | 1.93 | 2.15 | 1.95 | 2.00 | 1.93 | 1.99 |
|  | (0.83) | (0.84) | (0.76) | (0.82) | (0.87) | (0.85) | (0.76) | (0.90) |
| Size of municipality |  |  |  |  |  |  |  |  |
| Big city | 19.93 | 18.94 | 17.26 | 22.55 | 20.71 | 19.06 | 20.63 | 23.40 |
| Large town | 14.61 | 14.41 | 15.39 | 14.76 | 15.43 | 14.64 | 16.43 | 16.28 |
| Small town | 26.51 | 25.88 | 29.69 | 26.81 | 25.89 | 25.66 | 28.16 | 25.31 |
| Rural area | 31.29 | 29.83 | 34.22 | 33.23 | 30.31 | 29.28 | 31.17 | 31.63 |
| Charlston Comorbidity Index, Items |  |  |  |  |  |  |  |  |
| Item 1, A heart attack. myocardial infarction or coronary thrombosis (1 point) | 11.78 | 10.97 | 18.53 | 11.40 | 8.21 | 6.64 | 16.65 | 7.23 |
| Item 2, A stroke or cerebral vascular disease (1 point) | 3.11 | 2.39 | 9.17 | 2.77 | 2.44 | 1.64 | 5.91 | 2.28 |
| Item 3, Chronic lung disease such as chronic bronchitis or emphysema (1 point) | 6.01 | 5.48 | 11.16 | 5.55 | 5.50 | 4.43 | 9.83 | 5.41 |
| Item 4, Arthritis. including osteoarthritis. or rheumatism (1 point) | 18.51 | 16.83 | 28.97 | 18.72 | 33.37 | 29.21 | 48.00 | 33.99 |
| Item 4, Stomach or duodenal ulcer. peptic ulcer (1 point) | 2.42 | 2.19 | 4.04 | 2.39 | 2.64 | 2.19 | 4.29 | 2.69 |
| Item 6, Diabetes or high blood sugar (1 point) | 13.25 | 12.70 | 20.94 | 12.12 | 10.64 | 9.75 | 18.16 | 8.94 |
| Item 7, Cancer or malignant tumour. including leukaemia or lymphoma. but excluding minor skin cancers (2 points) | 7.12 | 5.82 | 14.24 | 7.57 | 7.06 | 6.19 | 11.20 | 6.73 |
| Charlston Comorbidity Index (final score) | 0.62 | 0.56 | 1.07 | 0.61 | 0.70 | 0.60 | 1.14 | 0.67 |
|  | (0.89) | (0.84) | (1.12) | (0.86) | (0.90) | (0.83) | (1.07) | (0.86) |
| Relation with economic activity |  |  |  |  |  |  |  |  |
| Working | 19.54 | 19.04 | 7.66 | 23.81 | 15.79 | 15.18 | 5.26 | 21.18 |
| Retired | 70.98 | 68.58 | 83.71 | 71.93 | 57.50 | 53.91 | 69.00 | 58.50 |
| Unemployed | 1.64 | 1.83 | 0.91 | 1.49 | 1.63 | 1.48 | 0.77 | 2.22 |
| Houseworking | 0.13 | 0.11 | 0.06 | 0.19 | 16.28 | 17.88 | 15.35 | 14.08 |
| Health at current moment |  |  |  |  |  |  |  |  |
| Excellent | 8.82 | 8.12 | 3.44 | 11.64 | 7.31 | 6.70 | 3.15 | 10.03 |
| Very good | 21.04 | 19.42 | 11.71 | 26.71 | 18.69 | 18.28 | 10.68 | 22.70 |
| Good | 38.33 | 39.46 | 29.93 | 38.57 | 37.48 | 38.55 | 30.07 | 38.85 |
| Fair | 21.47 | 20.82 | 33.49 | 19.29 | 24.80 | 23.02 | 35.69 | 23.13 |
| Poor | 5.59 | 4.23 | 21.30 | 3.75 | 7.38 | 5.32 | 20.35 | 5.29 |
| Noncoresident caregiver | 27.42 | 0.00 | 0.00 | 86.76 | 28.29 | 0.00 | 0.00 | 85.73 |
| Coresident caregiver | 5.69 | 0.00 | 0.00 | 18.02 | 6.65 | 0.00 | 0.00 | 20.16 |
| Current individual income (PPP2020) | 20,310 | 19,134 | 18,870 | 22,929 | 12,646 | 11,430 | 13,161 | 14,392 |
|  | (28,925) | (26,527) | (23,807) | (33,960) | (16,163) | (15,878) | (15,483) | (16,718) |
| Wealth adjusted by household size (PPP2020) | 238,084 | 234,182 | 200,858 | 255,193 | 211,920 | 214,580 | 169,888 | 224,024 |
|  | (309,965) | (298,253) | (252,344) | (342,333) | (295,297) | (285,803) | (281,510) | (312,856) |
| Current individual income (PPP2020) | 20,310 | 19,134 | 19,713 | 22,929 | 12,646 | 11,430 | 19,713 | 14,392 |
|  | (28,925) | (26,527) | (22,227) | (33,960) | (16,163) | (15,878) | (22,227) | (16,718) |
| Earnings | 5,869 | 5,558 | 4,254 | 7,400 | 3,317 | 3,026 | 4,254 | 4,683 |
|  | (14,165) | (14,212) | (11,731) | (15,007) | (8,705) | (8,250) | (11,731) | (10,256) |
| Unemployment benefits | 200 | 191 | 146 | 245 | 183 | 161 | 146 | 237 |
|  | (2,755) | (2,645) | (1,494) | (3,197) | (3,005) | (2,847) | (1,494) | (3,151) |
| Social assistance | 44 | 31 | 103 | 39 | 55 | 22 | 103 | 53 |
|  | (1,272) | (1,468) | (895) | (847) | (1,286) | (425) | (895) | (1,941) |
| Retirement benefits | 13,199 | 12,466 | 13,812 | 14,199 | 8,448 | 7,711 | 13,812 | 8,649 |
|  | (25,655) | (21,930) | (20,081) | (32,380) | (14,104) | (14,141) | (20,081) | (13,896) |
| Other benefits | 502 | 476 | 519 | 550 | 266 | 213 | 519 | 328 |
|  | (7,396) | (9,012) | (3,351) | (4,049) | (3,179) | (2,213) | (3,351) | (3,925) |
| Disability benefits | 496 | 413 | 879 | 494 | 377 | 296 | 879 | 443 |
|  | (4,982) | (4,402) | (6,651) | (4,561) | (3,422) | (2,839) | (6,651) | (4,214) |
| Current income from partner (PPP2020) | 14,322 | 13,540 | 12,277 | 16,253 | 20,333 | 22,999 | 19,494 | 20,596 |
|  | (28,478) | (23,660) | (14,597) | (37,543) | (25,762) | (25,909) | (12,551) | (29,333) |
|  |  |  |  |  |  |  |  |  |
| Current income from other household members (PPP2020) | 11,162 | 11,289 | 10,499 | 11,128 | 11,205 | 11,191 | 9,617 | 11,470 |
|  | (6,170) | (6,634) | (3,264) | (5,941) | (8,220) | (6,355) | (3,596) | (11,424) |

Source: own work using SHARE (waves 1, 2, 3, 4, 5, 6, 7 and 8).

Long-term income: discounted sum of total individual income (PPP2020) for the period 2007-2020, using an interest rate of 2%.

**Appendix B**

**Table B1. First step regressions for endogenous variables**

|  | Total | Men | Women |
| --- | --- | --- | --- |
| **Dependent variable: caregiver** |  |  |  |
| Single child | 0.1443*** | 0.0929*** | 0.1830*** |
|  | (0.016) | (0.023) | (0.021) |
| Eldest child | 0.1056*** | 0.0874*** | 0.1197*** |
|  | (0.016) | (0.024) | (0.021) |
| Youngest child | -0.0915*** | -0.0532** | -0.1165*** |
|  | (0.017) | (0.026) | (0.022) |
| Number of brothers | 0.0176*** | 0.0162*** | 0.0189*** |
|  | (0.002) | (0.003) | (0.003) |
| Number of sisters | -0.1004*** | -0.2833*** | -0.0822 *** |
|  | (0.003) | (0.004) | (0.003) |
| Constant | 0.3032*** | 0.3012*** | 0.3056*** |
|  | (0.004) | (0.007) | (0.006) |
| N | 44,208 | 18,648 | 25,560 |
| R2 | 0.3036 | 0.3032 | 0.3045 |
| F | 319.357 | 119.227 | 232.853 |
| p | 0.0000 | 0.0000 | 0.0000 |
| Partial R2 | 0.014 | 0.013 | 0.021 |
| F-statistic of excluded instruments | 1,201.23 | 983.09 | 1029.02 |
| Anderson LR statistic (p-value) | 0.0000 | 0.0000 | 0.0000 |
| Hansen J statistic (p-value) | 0.2467 | 0.3896 | 0.1468 |
| **Dependent variable: coresident caregiver** |  |  |  |
|  |  |  |  |
| Single child | 0.2091*** | 0.1165*** | 0.3032*** |
|  | (0.002) | (0.0103) | (0.001) |
| Eldest child | 0.2047*** | 0.1272*** | 0.3526*** |
|  | (0.002) | (0.002) | (0.001) |
| Youngest child | -0.0154**** | -0.0295** | -0.0064** |
|  | (0.003) | (0.013) | (0.001) |
| Number of brothers | 0.0026*** | 0.0025*** | 0.0027*** |
|  | (0.001) | (0.001) | (0.001) |
| Number of sisters | -0.0142*** | -0.0280*** | -0.0123*** |
|  | (0.001) | (0.002) | (0.002) |
| Constant | 0.0616*** | 0.0600*** | 0.0632*** |
|  | (0.002) | (0.003) | (0.003) |
| R2 | 0.2562 | 0.3676 | 0.2751 |
| F | 323.915 | 220.821 | 100.6198 |
| p | 0.0000 | 0.0000 | 0.0000 |
| Partial R2 | 0.010 | 0.011 | 0.017 |
| F-statistic of excluded instruments | 1,134.98 | 1,035.02 | 912.98 |
| Anderson LR statistic (p-value) | 0.0000 | 0.0000 | 0.0000 |
| Hansen J statistic (p-value) | 0.1348 | 0.1467 | 0.1853 |
| **Dependent variable: non-coresident caregiver** |  |  |  |
|  |  |  |  |
| Single child | 0.0404*** | 0.0069*** | 0.0796*** |
|  | (0.015) | (0.010) | (0.022) |
| Eldest child | 0.1144*** | 0.1007*** | 0.1249*** |
|  | (0.015) | (0.023) | (0.020) |
| Youngest child | -0.1043*** | -0.0769*** | -0.1222*** |
|  | (0.016) | (0.025) | (0.021) |
| Number of brothers | 0.0165*** | 0.0149*** | 0.0179*** |
|  | (0.002) | (0.003) | (0.003) |
| Number of sisters | 0.0031*** | -0.0051*** | -0.0024*** |
|  | (0.001) | (0.000) | (0.001) |
| Constant | 0.2595*** | 0.2571*** | 0.2617*** |
|  | (0.004) | (0.006) | (0.005) |
| R2 | 0.2440 | 0.4135 | 0.3248 |
| F | 358.690 | 130.807 | 248.456 |
| p | 0.0000 | 0.0000 | 0.0000 |
| Partial R2 | 0.022 | 0.021 | 0.028 |
| F-statistic of excluded instruments | 1,267.89 | 1,012.67 | 1,187.23 |
| Anderson LR statistic (p-value) | 0.0000 | 0.0000 | 0.0000 |
| Hansen J statistic (p-value) | 0.2789 | 0.4137 | 0.4214 |
| **Dependent variable: Log(current individual income PPP2020)** |  |  |  |
| Log(income partner PPP2020) | 0.0716*** | 0.0590*** | 0.0912*** |
|  | (0.017) | (0.011) | (0.012) |
| Log(income other household members PPP2020) | 0.0278*** | 0.0345*** | 0.0423*** |
|  | (0.004) | (0.005) | (0.004) |
| Constant | 9.2374*** | 9.1268*** | 9.3899*** |
|  | (0.005) | (0.006) | (0.006) |
| R2 | 0.1923 | 0.1259 | 0.2908 |
| F | 45,430.270 | 15,157.025 | 35,070.472 |
| p | 0.0000 | 0.0000 | 0.0000 |
| Partial R2 | 0.015 | 0.016 | 0.018 |
| F-statistic of excluded instruments | 986.23 | 914.05 | 1,003.11 |
| Anderson LR statistic (p-value) | 0.0000 | 0.0000 | 0.0000 |
| Hansen J statistic (p-value) | 0.1680 | 0.2375 | 0.2021 |
| **Dependent variable: Log(Long-term individual income 2007-2020; PPP2020)** |  |  |  |
| Log(income partner; 2007-2020, PPP2020) | 0.4032*** | 0.3219*** | 0.6210*** |
|  | (0.043) | (0.045) | (0.021) |
| Log(income other household members; 2007-2020; PPP2020) | 0.0681*** | 0.0490*** | 0.0801*** |
|  | (0.011) | (0.012) | (0.02) |
| Constant | 12.0224*** | 12.2721*** | 11.8476*** |
|  | (0.004) | (0.005) | (0.006) |
| R2 | 0.2207 | 0.2307 | 0.2737 |
| F | 27,995.370 | 26,617.574 | 9,160.795 |
| p | 0.0000 | 0.0000 | 0.0000 |
| Partial R2 | 0.026 | 0.029 | 0.031 |
| F-statistic of excluded instruments | 984.98 | 782.98 | 1,112.87 |
| Anderson LR statistic (p-value) | 0.0000 | 0.0000 | 0.0000 |
| Hansen J statistic (p-value) | 0.2361 | 0.1634 | 0.1789 |

Note: In the regressions for caregiver, coresident caregiver and non-coresident caregiver, the following are introduced as explanatory variables: age, sex, marital status, level of education, size of municipality of residence, wave fixed effects and country fixed effects.

In the regressions for current individual income (PPP2020) and long-term individual income (2007-2020; PPP2020), the following are introduced as explanatory variables: age, sex, marital status, household size, level of education and country fixed effects.

Instruments for caregiver, coresident caregiver and non-coresident caregiver: number of daughters, number of sons, being the eldest child, being the youngest child and being single child. Instruments for current individual income (PPP2020): income from partner and income from other household members (PPP2020).Instruments for long-term individual income (2007-2020; PPP2020): long-term income from partner (2007-2020; PPP2020) and long-term income from other household members (2007-2020; PPP2020). ***denotes significance at the 99% level, **at the 95% level, *at the 90% level.

**Table B2. Regression results for Fixed Effects Filtered Panel Models**

|  | Total | | Men | | Women | |
| --- | --- | --- | --- | --- | --- | --- |
|  | R1 | R2 | R1 | R2 | R1 | R2 |
| **First-step: Fixed effects model with time varying regressors** |  |  |  |  |  |  |
| IV(Caregiver) | -0.0780*** |  | -0.0403* |  | -0.1049*** |  |
|  | (0.017) |  | (0.024) |  | (0.024) |  |
| IV(Coresident caregiver) |  | -0.4322*** |  | -0.3603*** |  | -0.4860*** |
|  |  | (0.033) |  | (0.048) |  | (0.046) |
| IV(Non-coresident caregiver) |  | -0.0224*** |  | -0.0174*** |  | -0.0273*** |
|  |  | (0.008) |  | (0.011) |  | (0.002) |
| IV(log current individual income PPP2020) | 0.0247*** | 0.0262*** | 0.0331*** | 0.0346*** | 0.0247*** | 0.0264*** |
|  | (0.007) | (0.007) | (0.012) | (0.012) | (0.010) | (0.010) |
| Age | 0.0136*** | 0.0150*** | 0.0120*** | 0.0136*** | 0.0152*** | 0.0166*** |
|  | (0.001) | (0.001) | (0.002) | (0.002) | (0.002) | (0.002) |
| Married/cohabiting | 0.3959*** | 0.4073*** | 0.3530*** | 0.3656*** | 0.4201*** | 0.4335*** |
|  | (0.026) | (0.026) | (0.049) | (0.049) | (0.034) | (0.034) |
| Separated/divorced | -0.0360 | -0.0401 | 0.0375 | 0.0426 | -0.0799* | -0.0874** |
|  | (0.035) | (0.035) | (0.064) | (0.064) | (0.043) | (0.043) |
| Single | -0.0608 | -0.0512 | -0.1234* | -0.1024 | -0.0395 | -0.0382 |
|  | (0.041) | (0.041) | (0.067) | (0.066) | (0.056) | (0.055) |
| Household size | -0.0235** | -0.0069 | -0.0222 | -0.0101 | -0.0228 | -0.0008 |
|  | (0.012) | (0.012) | (0.016) | (0.016) | (0.017) | (0.017) |
| Big city | 0.0819*** | 0.0799*** | 0.0227 | 0.0244 | 0.1334*** | 0.1277*** |
|  | (0.022) | (0.022) | (0.031) | (0.031) | (0.031) | (0.031) |
| Large town | 0.0912*** | 0.0926*** | 0.0230 | 0.0293 | 0.1529*** | 0.1498*** |
|  | (0.024) | (0.024) | (0.034) | (0.034) | (0.034) | (0.034) |
| Small town | 0.0765*** | 0.0750*** | 0.0511* | 0.0535* | 0.1002*** | 0.0950*** |
|  | (0.021) | (0.021) | (0.028) | (0.028) | (0.029) | (0.029) |
| Charlston Comorbidity Index | -0.0573*** | -0.0574*** | -0.0675*** | -0.0678*** | -0.0508*** | -0.0509*** |
|  | (0.010) | (0.010) | (0.014) | (0.014) | (0.014) | (0.014) |
| Working | 0.2270*** | 0.2177*** | 0.3115*** | 0.3080*** | 0.1834*** | 0.1694*** |
|  | (0.046) | (0.046) | (0.071) | (0.071) | (0.061) | (0.061) |
| Retired | 0.1519*** | 0.1447*** | 0.2554*** | 0.2478*** | 0.0931* | 0.0877 |
|  | (0.042) | (0.042) | (0.067) | (0.067) | (0.055) | (0.055) |
| Unemployed | -0.2615*** | -0.2766*** | -0.1015*** | -0.1092*** | -0.3614*** | -0.3802*** |
|  | (0.075) | (0.075) | (0.011) | (0.011) | (0.102) | (0.101) |
| Homemaker | -0.0998* | -0.1014* | -0.0540*** | -0.0235** | -0.1567** | -0.1593** |
|  | (0.057) | (0.056) | (0.405) | (0.404) | (0.067) | (0.067) |
| Current health status: execellent | 1.2017*** | 1.1692*** | 0.1263 | 0.0738 | 2.1148*** | 2.1023*** |
|  | (0.260) | (0.259) | (0.356) | (0.356) | (0.373) | (0.372) |
| Current health status: very good | 0.8358*** | 0.8048*** | -0.2171 | -0.2679 | 1.7221*** | 1.7108*** |
|  | (0.259) | (0.258) | (0.355) | (0.354) | (0.371) | (0.370) |
| Current health status: good | 0.3930 | 0.3684 | -0.6145* | -0.6576* | 1.2444*** | 1.2384*** |
|  | (0.259) | (0.258) | (0.355) | (0.354) | (0.371) | (0.370) |
| Current health status: fair | -0.1024 | -0.1165 | -1.0486*** | -1.0830*** | 0.7041* | 0.7099* |
|  | (0.259) | (0.258) | (0.355) | (0.354) | (0.371) | (0.370) |
| Log (Wealth adjusted by household size PPP2020) | 0.0022*** | 0.0021*** | 0.0034*** | 0.0036*** | 0.0011*** | 0.0010*** |
|  | (0.000) | (0.000) | (0.000) | (0.000) | (0.000) | (0.000) |
| Constant | 5.3461*** | 5.2841*** | 6.3367*** | 6.2535*** | 4.4092*** | 4.3169*** |
|  | (0.294) | (0.293) | (0.415) | (0.414) | (0.420) | (0.419) |
| N | 312,890 | 312,890 | 140,470 | 140,470 | 172,420 | 172,420 |
| R2 | 0.3665 | 0.3704 | 0.3498 | 0.3532 | 0.3753 | 0.3797 |
| F | 249.8893 | 247.0370 | 98.8121 | 97.5271 | 146.3345 | 145.0108 |
| p | 0.0000 | 0.0000 | 0.0000 | 0.0000 | 0.0000 | 0.0000 |
| **Second step: Between model with time-invariant regressors** |  |  |  |  |  |  |
| IV(Log long-term individual income (PPP2020)) | 0.0904*** | 0.0846*** | 0.1002*** | 0.0968*** | 0.0896*** | 0.0848*** |
|  | (0.010) | (0.010) | (0.015) | (0.015) | (0.015) | (0.015) |
| Man | -0.0503*** | -0.0569*** |  |  |  |  |
|  | (0.011) | (0.011) |  |  |  |  |
| Period during which were happier than during the rest of his/her life | -0.5146*** | -0.5098*** | -0.5150*** | -0.5110*** | -0.5075*** | -0.5024*** |
|  | (0.023) | (0.023) | (0.033) | (0.033) | (0.031) | (0.031) |
| Age when happiness period started | 0.0095*** | 0.0094*** | 0.0077*** | 0.0077*** | 0.0109*** | 0.0108*** |
|  | (0.001) | (0.001) | (0.001) | (0.001) | (0.001) | (0.001) |
| Length of happiness period (years) | 0.0067*** | 0.0067*** | 0.0072*** | 0.0073*** | 0.0063*** | 0.0063*** |
|  | (0.000) | (0.000) | (0.001) | (0.001) | (0.001) | (0.001) |
| Period during which were under more stress than during the rest of his/her life | 0.1323*** | 0.1266*** | -0.0508 | -0.0554 | 0.2396*** | 0.2326*** |
|  | (0.026) | (0.026) | (0.038) | (0.038) | (0.036) | (0.036) |
| Age when stress period started | -0.0046*** | -0.0046*** | -0.0013* | -0.0013* | -0.0065*** | -0.0064*** |
|  | (0.001) | (0.001) | (0.001) | (0.001) | (0.001) | (0.001) |
| Length of stress period (years) | -0.0086*** | -0.0083*** | -0.0002 | -0.0000 | -0.0131*** | -0.0127*** |
|  | (0.001) | (0.001) | (0.001) | (0.001) | (0.001) | (0.001) |
| Period during which health was poorer compared to the rest of his/her your life | 0.1525*** | 0.1564*** | 0.2200*** | 0.2257*** | 0.1349*** | 0.1362*** |
|  | (0.028) | (0.028) | (0.040) | (0.040) | (0.039) | (0.039) |
| Age when poor health period started | -0.0020*** | -0.0021*** | -0.0030*** | -0.0031*** | -0.0020*** | -0.0020*** |
|  | (0.001) | (0.001) | (0.001) | (0.001) | (0.001) | (0.001) |
| Length of poor health period (years) | 0.0026*** | 0.0023*** | 0.0011 | 0.0009 | 0.0045*** | 0.0042*** |
|  | (0.001) | (0.001) | (0.001) | (0.001) | (0.001) | (0.001) |
| Period during which there was a distinct financial hardship | 0.1651*** | 0.1636*** | 0.1247*** | 0.1189*** | 0.1667*** | 0.1691*** |
|  | (0.026) | (0.026) | (0.039) | (0.039) | (0.035) | (0.035) |
| Age when financial hardship started | -0.0046*** | -0.0046*** | -0.0044*** | -0.0043*** | -0.0044*** | -0.0044*** |
|  | (0.001) | (0.001) | (0.001) | (0.001) | (0.001) | (0.001) |
| Length of financial hardship period (years) | -0.0121*** | -0.0120*** | -0.0081*** | -0.0080*** | -0.0142*** | -0.0141*** |
|  | (0.001) | (0.001) | (0.001) | (0.001) | (0.001) | (0.001) |
| Period during which suffered from hunger | 0.0608 | 0.0691* | 0.0730 | 0.0864 | -0.0356 | -0.0305 |
|  | (0.040) | (0.040) | (0.061) | (0.061) | (0.056) | (0.056) |
| Age when hunger period period started | -0.0049** | -0.0055*** | 0.0043 | 0.0033 | -0.0044* | -0.0048** |
|  | (0.002) | (0.002) | (0.004) | (0.004) | (0.002) | (0.002) |
| Length of hunger period (years) | -0.0180*** | -0.0186*** | -0.0284*** | -0.0294*** | -0.0047 | -0.0049 |
|  | (0.004) | (0.004) | (0.006) | (0.006) | (0.006) | (0.006) |
| Ever been the victim of such persecution or discrimination | -0.1063*** | -0.1087*** | -0.0178 | -0.0202 | -0.1506*** | -0.1538*** |
|  | (0.023) | (0.023) | (0.030) | (0.030) | (0.033) | (0.033) |
| Health during childhood: Excellent | -0.0412 | -0.0223 | 0.1814 | 0.1977 | -0.0978 | -0.0904 |
|  | (0.178) | (0.178) | (0.225) | (0.225) | (0.272) | (0.272) |
| Health during childhood: Very good | -0.0957 | -0.0732 | 0.0898 | 0.1084 | -0.1217 | -0.1102 |
|  | (0.178) | (0.178) | (0.225) | (0.225) | (0.273) | (0.272) |
| Health during childhood: Good | -0.1592 | -0.1388 | -0.0163 | 0.0027 | -0.1565 | -0.1494 |
|  | (0.178) | (0.178) | (0.225) | (0.225) | (0.273) | (0.272) |
| Health during childhood: Fair | -0.1177 | -0.1003 | 0.0348 | 0.0553 | -0.1161 | -0.1156 |
|  | (0.179) | (0.179) | (0.227) | (0.227) | (0.274) | (0.273) |
| Health during childhood: Poor | -0.1538 | -0.1287 | -0.0897 | -0.0537 | -0.0913 | -0.0891 |
|  | (0.180) | (0.180) | (0.229) | (0.229) | (0.275) | (0.274) |
| During your childhood. ever in hospital for >=1 month | 0.0042 | 0.0035 | 0.0381 | 0.0336 | -0.0294 | -0.0260 |
|  | (0.021) | (0.021) | (0.028) | (0.028) | (0.029) | (0.029) |
| A physical injury that has led to any permanent handicap | 0.0441*** | 0.0434*** | -0.0373* | -0.0370* | 0.0993*** | 0.0980*** |
|  | (0.014) | (0.014) | (0.019) | (0.019) | (0.021) | (0.021) |
| None or very few (0-10 books) | -0.0740*** | -0.0700*** | -0.0363 | -0.0327 | -0.1081*** | -0.1036*** |
|  | (0.021) | (0.021) | (0.029) | (0.029) | (0.030) | (0.029) |
| Enough to fill one shelf (11-25 books) | -0.0816*** | -0.0833*** | -0.0375 | -0.0411 | -0.1219*** | -0.1215*** |
|  | (0.021) | (0.021) | (0.029) | (0.029) | (0.029) | (0.029) |
| Enough to fill one bookcase (26-100 books) | 0.0476** | 0.0439** | 0.1593*** | 0.1518*** | -0.0365 | -0.0372 |
|  | (0.019) | (0.019) | (0.027) | (0.027) | (0.027) | (0.027) |
| Enough to fill two bookcases (101-200 books) | 0.0454* | 0.0438* | 0.1188*** | 0.1147*** | -0.0083 | -0.0075 |
|  | (0.023) | (0.023) | (0.033) | (0.033) | (0.032) | (0.032) |
| Performance Maths: Much better | 0.1437*** | 0.1378*** | 0.1122** | 0.1016* | 0.0750 | 0.0755 |
|  | (0.038) | (0.038) | (0.055) | (0.055) | (0.052) | (0.052) |
| Performance Maths: Better | 0.0776** | 0.0745** | 0.0386 | 0.0283 | 0.0356 | 0.0409 |
|  | (0.036) | (0.036) | (0.054) | (0.054) | (0.048) | (0.048) |
| Performance Maths: About the same | 0.1250*** | 0.1187*** | 0.0477 | 0.0361 | 0.1093** | 0.1102** |
|  | (0.035) | (0.035) | (0.053) | (0.053) | (0.047) | (0.047) |
| Performance Maths: Worse | 0.0273 | 0.0191 | -0.1065* | -0.1204** | 0.0570 | 0.0562 |
|  | (0.037) | (0.037) | (0.056) | (0.056) | (0.050) | (0.049) |
| Performance Maths: Much worse | 0.0981 | 0.0904 | 0.3334*** | 0.3215*** | -0.1028 | -0.0946 |
|  | (0.077) | (0.077) | (0.101) | (0.101) | (0.115) | (0.115) |
| Performance Language: Much better | 0.0396 | 0.0237 | 0.2886*** | 0.2775*** | -0.1065 | -0.1175 |
|  | (0.071) | (0.071) | (0.096) | (0.096) | (0.105) | (0.104) |
| Performance Language: Better | -0.0016 | -0.0128 | 0.2231** | 0.2172** | -0.1321 | -0.1387 |
|  | (0.070) | (0.070) | (0.095) | (0.094) | (0.103) | (0.103) |
| Performance Language: About the same | -0.0309 | -0.0421 | 0.2323** | 0.2259** | -0.1766* | -0.1836* |
|  | (0.070) | (0.070) | (0.094) | (0.094) | (0.103) | (0.103) |
| Performance Language: Worse | -0.0266 | -0.0401 | 0.2872*** | 0.2784*** | -0.2505** | -0.2592** |
|  | (0.071) | (0.071) | (0.095) | (0.095) | (0.106) | (0.105) |
| Performance Language: Much worse | 0.0635 | 0.0559 | 0.4060*** | 0.3912*** | -0.2548** | -0.2390** |
|  | (0.078) | (0.078) | (0.100) | (0.100) | (0.119) | (0.119) |
| Age 10: lived with biological father | -0.0251 | -0.0196 | -0.0628* | -0.0543 | 0.0079 | 0.0100 |
|  | (0.027) | (0.027) | (0.038) | (0.038) | (0.037) | (0.037) |
| Age 10: lived with biological mother | 0.0885*** | 0.0879*** | 0.1062*** | 0.1038*** | 0.0756*** | 0.0770*** |
|  | (0.019) | (0.019) | (0.026) | (0.026) | (0.026) | (0.026) |
| Age 10: lived with stepfather | 0.2112*** | 0.2066*** | 0.0327 | 0.0354 | 0.3387*** | 0.3258*** |
|  | (0.042) | (0.042) | (0.060) | (0.060) | (0.057) | (0.057) |
| Age 10: lived with stepmother | -0.0686* | -0.0730** | 0.0897* | 0.0852* | -0.1440*** | -0.1479*** |
|  | (0.035) | (0.035) | (0.050) | (0.050) | (0.049) | (0.049) |
| Fixed bath | -0.0395*** | -0.0333** | -0.0242 | -0.0187 | -0.0586*** | -0.0521** |
|  | (0.015) | (0.015) | (0.021) | (0.021) | (0.021) | (0.021) |
| Cold running water supply | 0.0500*** | 0.0487*** | 0.0028 | 0.0038 | 0.0811*** | 0.0781*** |
|  | (0.014) | (0.014) | (0.019) | (0.019) | (0.020) | (0.020) |
| Hot running water supply | 0.0376** | 0.0337** | 0.0400* | 0.0385* | 0.0414* | 0.0351 |
|  | (0.016) | (0.016) | (0.022) | (0.022) | (0.022) | (0.022) |
| Inside toilet | 0.0073 | 0.0069 | 0.0298 | 0.0243 | -0.0161 | -0.0112 |
|  | (0.014) | (0.014) | (0.019) | (0.019) | (0.019) | (0.019) |
| Central heating | 0.0733*** | 0.0665*** | 0.0602*** | 0.0539** | 0.0726*** | 0.0650*** |
|  | (0.015) | (0.015) | (0.021) | (0.021) | (0.021) | (0.021) |
| Lived in a children’s home | 0.1674*** | 0.1694*** | 0.3204*** | 0.3156*** | 0.0337 | 0.0418 |
|  | (0.037) | (0.037) | (0.056) | (0.056) | (0.051) | (0.051) |
| Been fostered with another family | 0.1129*** | 0.1146*** | 0.0739 | 0.0603 | 0.1509*** | 0.1660*** |
|  | (0.038) | (0.038) | (0.054) | (0.054) | (0.053) | (0.053) |
| Evacuated or relocated during a war | -0.0619** | -0.0697*** | -0.1294*** | -0.1351*** | 0.0146 | 0.0054 |
|  | (0.027) | (0.026) | (0.035) | (0.035) | (0.039) | (0.039) |
| Lived in a prisoner of war camp | 0.4343*** | 0.4611*** | 0.2847** | 0.3030** | 0.6023*** | 0.6461*** |
|  | (0.114) | (0.114) | (0.128) | (0.128) | (0.218) | (0.218) |
| Lived in a prison | -0.0496 | -0.0567 | -0.0224 | -0.0320 | -0.3152 | -0.2988 |
|  | (0.086) | (0.086) | (0.090) | (0.090) | (0.192) | (0.192) |
| Lived in a labor camp | 0.1059 | 0.1122 | 0.3917*** | 0.3989*** | -0.1172 | -0.1129 |
|  | (0.091) | (0.091) | (0.118) | (0.118) | (0.136) | (0.136) |
| Lived in a concentration camp | -0.7567*** | -0.7574*** | 0.0000 | 0.0000 | -0.7296*** | -0.7301*** |
|  | (0.166) | (0.166) | (.) | (.) | (0.176) | (0.175) |
| Been an inpatient in a tuberculosis institution | -0.0342 | -0.0184 | -0.1847** | -0.1418 | 0.0931 | 0.0879 |
|  | (0.061) | (0.061) | (0.086) | (0.086) | (0.085) | (0.084) |
| Stayed in a psychiatric hospital | -0.2813*** | -0.2854*** | -0.8318*** | -0.8481*** | -0.0164 | -0.0168 |
|  | (0.067) | (0.067) | (0.105) | (0.105) | (0.087) | (0.087) |
| Been homeless for 1 month or more | -0.0637 | -0.0537 | 0.2562* | 0.2493* | -0.2812** | -0.2568* |
|  | (0.097) | (0.097) | (0.135) | (0.135) | (0.138) | (0.137) |
| Born in other country | -0.1021*** | -0.1009*** | -0.0831*** | -0.0808*** | -0.1340*** | -0.1332*** |
|  | (0.015) | (0.015) | (0.021) | (0.021) | (0.020) | (0.020) |
| Lower secondary education | 0.0175 | 0.0139 | -0.0461** | -0.0476** | 0.0570*** | 0.0530** |
|  | (0.016) | (0.016) | (0.022) | (0.022) | (0.022) | (0.022) |
| Upper secondary education | 0.0416*** | 0.0363** | -0.0549*** | -0.0572*** | 0.0973*** | 0.0886*** |
|  | (0.014) | (0.014) | (0.020) | (0.020) | (0.020) | (0.020) |
| Postsecondary nontertiary education | 0.1381*** | 0.1318*** | 0.1701*** | 0.1648*** | 0.0815** | 0.0762* |
|  | (0.028) | (0.028) | (0.039) | (0.039) | (0.040) | (0.040) |
| First stage of tertiary education | 0.0017 | -0.0034 | -0.0872*** | -0.0911*** | 0.0643*** | 0.0569** |
|  | (0.017) | (0.017) | (0.023) | (0.023) | (0.024) | (0.024) |
| Second stage of tertiary education | -0.1853** | -0.1587** | -0.1427* | -0.1368 | -0.2528* | -0.1775 |
|  | (0.075) | (0.075) | (0.085) | (0.085) | (0.134) | (0.134) |
| Constant | -0.2433 | -0.2604 | -0.7336*** | -0.7501*** | -0.0691 | -0.0901 |
|  | (0.188) | (0.188) | (0.266) | (0.266) | (0.271) | (0.270) |
| r2 | 0.2551 | 0.2539 | 0.2698 | 0.2682 | 0.2618 | 0.2600 |
| F | 380.835 | 372.246 | 221.189 | 215.836 | 245.061 | 237.304 |
| p | 0.0000 | 0.0000 | 0.0000 | 0.0000 | 0.0000 | 0.0000 |

Note: Omitted variables in the regressions for life satisfaction: widow, other relation with economic activity, living in rural area, current health status (poor). All regressions include year (wave) specific effects. Omitted variables in the regressions for long-term individual income (2007-2020; PPP2020): Performance in Maths/Language at the age of 10 (did not go to school), self-reported health status at the age of 10 (varied from time to time), number of books at home at the age of 10 (more than 200 books), education (not completed primary education).Caregiving is instrumented using the following variables: being the eldest child, being the youngest child, being single child, number of brothers and number of sisters. Current individual income (PPP2020) is instrumented using income from partner and income from other household members. Long-term individual income (s007-2020; PPP2020) is instrumented using long-term income from partner y long-term income from other household members.

Standard errors are adjusted for clustering on country and year level. ***denotes significance at the 99% level, **at the 95% level, *at the 90% level.

**Table B3. Regression results for Fixed Effects Filtered Panel Models by country**

|  | Total | | Men | | Women | |
| --- | --- | --- | --- | --- | --- | --- |
|  | R1 | R2 | R1 | R2 | R1 | R2 |
| **Austria** |  |  |  |  |  |  |
| **First-step: Fixed effects model with time varying regressors** |  |  |  |  |  |  |
| IV(Caregiver) | -0.1739*** |  | -0.0786*** |  | -0.1293*** |  |
|  | (0.024) |  | (0.035) |  | (0.025) |  |
| IV(Coresident caregiver) |  | -0.3024*** |  | -0.0971*** |  | -0.3768*** |
|  |  | (0.105) |  | (0.021) |  | (0.132) |
| IV(Non-coresident caregiver) |  | -0.0677*** |  | -0.0556*** |  | -0.1163*** |
|  |  | (0.029) |  | (0.011) |  | (0.032) |
| IV(log current individual income PPP2020) | 0.1217*** | 0.1123*** | 0.2002*** | 0.1103*** | 0.1123*** | 0.1083 *** |
|  | (0.037) | (0.037) | (0.128) | (0.128) | (0.036) | (0.036) |
| N | 1,190 | 1,190 | 447 | 447 | 743 | 7.3 |
| R2 | 0.2416 | 0.2433 | 0.2852 | 0.2851 | 0.2556 | 0.2579 |
| F | 148.353 | 143.851 | 70.163 | 67.157 | 98.494 | 95.700 |
| p | 0.0000 | 0.0000 | 0.0000 | 0.0000 | 0.0000 | 0.0000 |
| **Second step: Between model with time-invariant regressors** |  |  |  |  |  |  |
| IV( Log long-term individual income (PPP2020)) | 0.1881*** | 0.1804*** | 0.2133** | 0.2116** | 0.1353*** | 0.1494*** |
|  | (0.023) | (0.023) | (0.029) | (0.029) | (0.007) | (0.007) |
| R2 | 0.2949 | 0.2951 | 0.6072 | 0.6074 | 0.4349 | 0.4299 |
| F | 108.698 | 108.803 | 149.921 | 149.988 | 134.887 | 132.147 |
| p | 0.0000 | 0.0000 | 0.0000 | 0.0000 | 0.0000 | 0.0000 |
| **Germany** |  |  |  |  |  |  |
| **First-step: Fixed effects model with time varying regressors** |  |  |  |  |  |  |
| IV(Caregiver) | -0.1304** |  | -0.1445*** |  | -0.1320* |  |
|  | (0.057) |  | (0.054) |  | (0.077) |  |
| IV(Coresident caregiver) |  | -0.5621*** |  | -0.7671*** |  | -0.4369*** |
|  |  | (0.111) |  | (0.173) |  | (0.146) |
| IV(Non-coresident caregiver) |  | -0.0632*** |  | -0.0410*** |  | -0.0932*** |
|  |  | (0.029) |  | (0.017) |  | (0.020) |
| IV(log current individual income PPP2020) | 0.0675** | 0.0607* | 0.1546*** | 0.1497*** | 0.0156*** | 0.0115** |
|  | (0.032) | (0.032) | (0.055) | (0.055) | (0.005) | (0.005) |
| N | 2,885 | 2,885 | 1,356 | 1,356 | 1,529 | 1,529 |
| R2 | 0.1925 | 0.1986 | 0.2463 | 0.2557 | 0.1674 | 0.1716 |
| F | 272.655 | 272.422 | 189.218 | 190.572 | 120.850 | 119.667 |
| p | 0.0000 | 0.0000 | 0.0000 | 0.0000 | 0.0000 | 0.0000 |
| **Second step: Between model with time-invariant regressors** |  |  |  |  |  |  |
| IV( Log long-term individual income (PPP2020)) | 0.1026*** | 0.1047*** | 0.1120*** | 0.1140*** | 0.0940*** | 0.0845*** |
|  | (0.026) | (0.026) | (0.045) | (0.004) | (0.031) | (0.031) |
| R2 | 0.1689 | 0.1682 | 0.2472 | 0.2495 | 0.2269 | 0.2243 |
| F | 122.648 | 122.093 | 92.523 | 93.626 | 97.598 | 96.166 |
| p | 0.0000 | 0.0000 | 0.0000 | 0.0000 | 0.0000 | 0.0000 |
| **Sweden** |  |  |  |  |  |  |
| **First-step: Fixed effects model with time varying regressors** |  |  |  |  |  |  |
| IV(Caregiver) | -0.1223** |  | -0.1205* |  | -0.1148*** |  |
|  | (0.051) |  | (0.071) |  | (0.022) |  |
| IV(Coresident caregiver) |  | -0.5423*** |  | -0.4251** |  | -0.6209*** |
|  |  | (0.130) |  | (0.194) |  | (0.174) |
| IV(Non-coresident caregiver) |  | -0.0724*** |  | -0.0699*** |  | -0.0588*** |
|  |  | (0.022) |  | (0.023) |  | (0.013) |
| IV(log current individual income PPP2020) | 0.0415*** | 0.0317*** | 0.0810*** | 0.0686*** | 0.0095*** | 0.0053*** |
|  | (0.009) | (0.009) | (0.014) | (0.014) | (0.002) | (0.002) |
| N | 2,832 | 2,832 | 1,169 | 1,169 | 1,663 | 1,663 |
| R2 | 0.1454 | 0.1497 | 0.2027 | 0.2048 | 0.1404 | 0.1463 |
| F | 190.912 | 189.953 | 121.181 | 117.762 | 106.937 | 107.826 |
| p | 0.0000 | 0.0000 | 0.0000 | 0.0000 | 0.0000 | 0.0000 |
| **Second step: Between model with time-invariant regressors** |  |  |  |  |  |  |
| IV( Log long-term individual income (PPP2020)) | 0.1599*** | 0.1571*** | 0.2137*** | 0.2089*** | 0.0539*** | 0.0462*** |
|  | (0.043) | (0.043) | (0.061) | (0.061) | (0.018) | (0.017) |
| R2 | 0.1513 | 0.1494 | 0.3035 | 0.3011 | 0.2139 | 0.2095 |
| F | 101.013 | 99.535 | 104.566 | 103.408 | 92.469 | 90.090 |
| p | 0.0000 | 0.0000 | 0.0000 | 0.0000 | 0.0000 | 0.0000 |
| **Spain** |  |  |  |  |  |  |
| **First-step: Fixed effects model with time varying regressors** |  |  |  |  |  |  |
| IV(Caregiver) | -0.3119*** |  | -0.2460*** |  | -0.2646** |  |
|  | (0.082) |  | (0.107) |  | (0.126) |  |
| IV(Coresident caregiver) |  | -0.5458*** |  | -0.6613*** |  | -0.4198** |
|  |  | (0.104) |  | (0.131) |  | (0.166) |
| IV(Non-coresident caregiver) |  | -0.0070 |  | -0.1852 |  | -0.1114 |
|  |  | (0.111) |  | (0.156) |  | (0.159) |
| IV(log current individual income PPP2020) | 0.0618** | 0.0673** | 0.0752*** | 0.0714*** | 0.0476*** | 0.0440*** |
|  | (0.027) | (0.027) | (0.032) | (0.032) | (0.017) | (0.017) |
| N | 2,741 | 2,741 | 1,469 | 1,469 | 1,272 | 1,272 |
| R2 | 0.1519 | 0.1559 | 0.1251 | 0.1347 | 0.1641 | 0.1658 |
| F | 194.525 | 192.834 | 86.026 | 89.860 | 97.834 | 95.204 |
| p | 0.0000 | 0.0000 | 0.0000 | 0.0000 | 0.0000 | 0.0000 |
| **Second step: Between model with time-invariant regressors** |  |  |  |  |  |  |
| IV( Log long-term individual income (PPP2020)) | 0.0750** | 0.0703*** | 0.0815*** | 0.0961*** | 0.0522*** | 0.0578*** |
|  | (0.023) | (0.022) | (0.030) | (0.030) | (0.017) | (0.017) |
| R2 | 0.1206 | 0.1209 | 0.2160 | 0.2084 | 0.1838 | 0.1863 |
| F | 94.284 | 94.554 | 95.214 | 90.976 | 83.057 | 84.423 |
| p | 0.0000 | 0.0000 | 0.0000 | 0.0000 | 0.0000 | 0.0000 |
| **Italy** |  |  |  |  |  |  |
| **First-step: Fixed effects model with time varying regressors** |  |  |  |  |  |  |
| IV(Caregiver) | -0.2401*** |  | -0.1057** |  | -0.2948*** |  |
|  | (0.056) |  | (0.078) |  | (0.081) |  |
| IV(Coresident caregiver) |  | -0.4750*** |  | -0.3700*** |  | -0.5299*** |
|  |  | (0.082) |  | (0.122) |  | (0.112) |
| IV(Non-coresident caregiver) |  | -0.1655*** |  | -0.0185*** |  | -0.1322 |
|  |  | (0.025) |  | (0.068) |  | (0.096) |
| IV(log current individual income PPP2020) | 0.0672*** | 0.0605*** | 0.0659**** | 0.0603**** | 0.0598*** | 0.0613*** |
|  | (0.030) | (0.029) | (0.025) | (0.025) | (0.018) | (0.018) |
| N | 3,910 | 3,910 | 1,901 | 1,901 | 2,009 | 2,009 |
| R2 | 0.1520 | 0.1556 | 0.1189 | 0.1214 | 0.1727 | 0.1772 |
| F | 278.499 | 275.281 | 101.237 | 99.571 | 172.543 | 170.824 |
| p | 0.0000 | 0.0000 | 0.0000 | 0.0000 | 0.0000 | 0.0000 |
| **Second step: Between model with time-invariant regressors** |  |  |  |  |  |  |
| IV( Log long-term individual income (PPP2020)) | 0.0831*** | 0.0822*** | 0.0653*** | 0.0678*** | 0.0935*** | 0.0911*** |
|  | (0.021) | (0.021) | (0.026) | (0.026) | (0.027) | (0.027) |
| R2 | 0.1114 | 0.1075 | 0.1648 | 0.1659 | 0.1576 | 0.1508 |
| F | 119.586 | 114.894 | 87.751 | 88.489 | 103.296 | 98.096 |
| p | 0.0000 | 0.0000 | 0.0000 | 0.0000 | 0.0000 | 0.0000 |
| **France** |  |  |  |  |  |  |
| **First-step: Fixed effects model with time varying regressors** |  |  |  |  |  |  |
| IV(Caregiver) | -0.1882*** |  | -0.2284** |  | -0.1614*** |  |
|  | (0.053) |  | (0.075) |  | (0.054) |  |
| IV(Coresident caregiver) |  | -0.2575** |  | -0.3317** |  | -0.2163*** |
|  |  | (0.103) |  | (0.137) |  | (0.091) |
| IV(Non-coresident caregiver) |  | -0.1335** |  | -0.1400*** |  | -0.1158*** |
|  |  | (0.056) |  | (0.060) |  | (0.048) |
| IV(log current individual income PPP2020) | 0.0894*** | 0.0882*** | 0.1735*** | 0.1666*** | 0.0596** | 0.0574** |
|  | (0.024) | (0.024) | (0.049) | (0.048) | (0.029) | (0.029) |
| N | 3,042 | 3,042 | 1,297 | 1,297 | 1,745 | 1,745 |
| r2 | 0.1720 | 0.1733 | 0.1451 | 0.1483 | 0.1892 | 0.1898 |
| F | 250.534 | 243.015 | 86.271 | 85.025 | 160.421 | 154.834 |
| p | 0.0000 | 0.0000 | 0.0000 | 0.0000 | 0.0000 | 0.0000 |
| **Second step: Between model with time-invariant regressors** |  |  |  |  |  |  |
| IV( Log long-term individual income (PPP2020)) | 0.1132*** | 0.1124*** | 0.0650*** | 0.0677*** | 0.1430*** | 0.1417*** |
|  | (0.034) | (0.034) | (0.010) | (0.010) | (0.048) | (0.048) |
| R2 | 0.1703 | 0.1707 | 0.2890 | 0.2894 | 0.2241 | 0.2243 |
| F | 127.060 | 127.408 | 107.246 | 107.424 | 105.679 | 105.754 |
| p | 0.0000 | 0.0000 | 0.0000 | 0.0000 | 0.0000 | 0.0000 |
| **Denmark** |  |  |  |  |  |  |
| **First-step: Fixed effects model with time varying regressors** |  |  |  |  |  |  |
| IV(Caregiver) | -0.1300*** |  | -0.0325*** |  | -0.1866*** |  |
|  | (0.018) |  | (0.006) |  | (0.012) |  |
| IV(Coresident caregiver) |  | -0.1604*** |  | -0.0556*** |  | -0.2735** |
|  |  | (0.070) |  | (0.027) |  | (0.108) |
| IV(Non-coresident caregiver) |  | -0.0931** |  | -0.0214*** |  | -0.1098** |
|  |  | (0.039) |  | (0.010) |  | (0.033) |
| IV(log current individual income PPP2020) | 0.0361*** | 0.0387*** | 0.0045*** | 0.0029*** | 0.0519*** | 0.0538 *** |
|  | (0.012) | (0.012) | (0.011) | (0.010) | (0.014) | (0.014) |
| N | 4,509 | 4,509 | 1,956 | 1,956 | 2,553 | 2,553 |
| R2 | 0.1448 | 0.1462 | 0.1454 | 0.1457 | 0.1646 | 0.1674 |
| F | 303.610 | 295.080 | 131.334 | 126.507 | 199.099 | 195.394 |
| p | 0.0000 | 0.0000 | 0.0000 | 0.0000 | 0.0000 | 0.0000 |
| **Second step: Between model with time-invariant regressors** |  |  |  |  |  |  |
| IV( Log long-term individual income (PPP2020)) | 0.1093*** | 0.1006*** | 0.1555*** | 0.1556*** | 0.0540*** | 0.0526*** |
|  | (0.029) | (0.029) | (0.041) | (0.041) | (0.010) | (0.010) |
| R2 | 0.2108 | 0.2113 | 0.2563 | 0.2561 | 0.1400 | 0.1410 |
| F | 117.365 | 117.973 | 145.926 | 145.833 | 90.864 | 91.628 |
| p | 0.0000 | 0.0000 | 0.0000 | 0.0000 | 0.0000 | 0.0000 |
| **Switzerland** |  |  |  |  |  |  |
| **First-step: Fixed effects model with time varying regressors** |  |  |  |  |  |  |
| IV(Caregiver) | -0.2575*** |  | -0.0939*** |  | -0.5910*** |  |
|  | (0.049) |  | (0.024) |  | (0.066) |  |
| IV(Coresident caregiver) |  | -0.4928*** |  | -0.1193*** |  | -0.6374*** |
|  |  | (0.135) |  | (0.025) |  | (0.178) |
| IV(Non-coresident caregiver) |  | -0.1135** |  | -0.0475*** |  | -0.3041 *** |
|  |  | (0.051) |  | (0.010) |  | (0.028) |
| IV(log current individual income PPP2020) | 0.0466*** | 0.0459*** | 0.1136*** | 0.1138*** | 0.0070*** | 0.0060*** |
|  | (0.016) | (0.016) | (0.040) | (0.040) | (0.028) | (0.028) |
| N | 2,732 | 2,732 | 1,145 | 1,145 | 1,587 | 1,587 |
| R2 | 0.1697 | 0.1724 | 0.1860 | 0.1864 | 0.1736 | 0.1824 |
| F | 221.294 | 216.790 | 102.283 | 98.506 | 131.125 | 133.882 |
| p | 0.0000 | 0.0000 | 0.0000 | 0.0000 | 0.0000 | 0.0000 |
| **Second step: Between model with time-invariant regressors** |  |  |  |  |  |  |
| IV( Log long-term individual income (PPP2020)) | 0.1266*** | 0.1264*** | 0.1463*** | 0.1461*** | 0.0591** | 0.0537* |
|  | (0.023) | (0.023) | (0.035) | (0.035) | (0.029) | (0.029) |
| R2 | 0.1704 | 0.1717 | 0.3354 | 0.3350 | 0.2302 | 0.2371 |
| F | 114.875 | 115.957 | 117.484 | 117.270 | 103.729 | 107.758 |
| p | 0.0000 | 0.0000 | 0.0000 | 0.0000 | 0.0000 | 0.0000 |
| **Belgium** |  |  |  |  |  |  |
| **First-step: Fixed effects model with time varying regressors** |  |  |  |  |  |  |
| IV(Caregiver) | -0.0741*** |  | -0.0517*** |  | -0.1113** |  |
|  | (0.017) |  | (0.011) |  | (0.054) |  |
| IV(Coresident caregiver) |  | -0.0302*** |  | -0.0614*** |  | -0.1487*** |
|  |  | (0.010) |  | (0.023) |  | (0.055) |
| IV(Non-coresident caregiver) |  | -0.1047*** |  | -0.0341*** |  | -0.0173*** |
|  |  | (0.038) |  | (0.009) |  | (0.006) |
| IV(log current individual income PPP2020) | 0.0403** | 0.0404** | 0.0515*** | 0.0510*** | 0.0328*** | 0.0331*** |
|  | (0.019) | (0.019) | (0.019) | (0.019) | (0.007) | (0.007) |
| N | 4,324 | 4,324 | 2,120 | 2,120 | 2,204 | 2,204 |
| R2 | 0.1473 | 0.1474 | 0.1338 | 0.1338 | 0.1503 | 0.1508 |
| F | 296.939 | 285.800 | 129.415 | 124.368 | 154.057 | 148.661 |
| p | 0.0000 | 0.0000 | 0.0000 | 0.0000 | 0.0000 | 0.0000 |
| **Second step: Between model with time-invariant regressors** |  |  |  |  |  |  |
| IV( Log long-term individual income (PPP2020)) | 0.0929*** | 0.0932*** | 0.0676*** | 0.0678*** | 0.1270*** | 0.1269*** |
|  | (0.013) | (0.013) | (0.022) | (0.022) | (0.017) | (0.017) |
| R2 | 0.1457 | 0.1457 | 0.2164 | 0.2168 | 0.1692 | 0.1702 |
| F | 173.844 | 173.935 | 133.030 | 133.376 | 112.872 | 113.663 |
| p | 0.0000 | 0.0000 | 0.0000 | 0.0000 | 0.0000 | 0.0000 |
| **Czech Republic** |  |  |  |  |  |  |
| **First-step: Fixed effects model with time varying regressors** |  |  |  |  |  |  |
| IV(Caregiver) | -0.2078*** |  | -0.0661*** |  | -0.2698*** |  |
|  | (0.063) |  | (0.002) |  | (0.082) |  |
| IV(Coresident caregiver) |  | -0.4536*** |  | -0.0817*** |  | -0.6355*** |
|  |  | (0.112) |  | (0.012) |  | (0.142) |
| IV(Non-coresident caregiver) |  | -0.0703*** |  | -0.0614*** |  | -0.0877*** |
|  |  | (0.016) |  | (0.014) |  | (0.016) |
| IV(log current individual income PPP2020) | 0.0957** | 0.0912** | 0.0338** | 0.0353** | 0.1137** | 0.1097** |
|  | (0.041) | (0.041) | (0.014) | (0.014) | (0.054) | (0.054) |
| N | 3,124 | 3,124 | 1,187 | 1,187 | 1,937 | 1,937 |
| R2 | 0.1419 | 0.1446 | 0.1359 | 0.1362 | 0.1572 | 0.1620 |
| F | 213.442 | 209.408 | 79.500 | 76.372 | 148.625 | 147.815 |
| p | 0.0000 | 0.0000 | 0.0000 | 0.0000 | 0.0000 | 0.0000 |
| **Second step: Between model with time-invariant regressors** |  |  |  |  |  |  |
| IV( Log long-term individual income (PPP2020)) | 0.2404*** | 0.2471*** | 0.1781** | 0.1780** | 0.2927*** | 0.3052*** |
|  | (0.051) | (0.051) | (0.089) | (0.089) | (0.064) | (0.064) |
| R2 | 0.1757 | 0.1775 | 0.2249 | 0.2260 | 0.2299 | 0.2317 |
| F | 145.108 | 146.937 | 78.919 | 79.434 | 131.775 | 133.159 |
| p | 0.0000 | 0.0000 | 0.0000 | 0.0000 | 0.0000 | 0.0000 |

Note: Explanatory variables in the regressions for life satisfaction: same as in Table B2

Explanator variables in the regressions for long-term individual income (2007-2020; PPP2020): same as in Table B2

Omitted variables in the regressions for life satisfaction: widow, other relation with economic activity, living in rural area, current health status (poor). All regressions include year (wave) specific effects.

Omitted variables in the regressions for long-term individual income (2007-2020; PPP2020): Performance in Maths/Language at the age of 10 (did not go to school), self-reported health status at the age of 10 (varied from time to time), number of books at home at the age of 10 (more than 200 books), education (not completed primary education).

Caregiving is instrumented using the following variables: being the eldest child, being the youngest child, being single child, number of brothers and number of sisters.

Current individual income (PPP2020) is instrumented using income from partner and income from other household members.

Long-term individual income (2007-2020; PPP2020) is instrumented using long-term income from partner y long-term income from other household members.

Standard errors are adjusted for clustering on year level.

***denotes significance at the 99% level, **at the 95% level, *at the 90% level.

**Table B4. Comparison of estimations of the fixed effects filtered model with and without IV**

|  | With IV | | | | | | Without IV | | | | | |  |
| --- | --- | --- | --- | --- | --- | --- | --- | --- | --- | --- | --- | --- | --- |
|  | Total | | Men | | Women | | Total | | Men | | Women | |  |
|  | R1 | R2 | R1 | R1 | R1 | R1 | R2 | R2 | R2 | R2 | R1 | R2 | |
| **All sample** |  |  |  |  |  |  |  |  |  |  |  |  | |
| Caregiver | -0.0780*** |  | -0.0403* |  | -0.1049*** |  | -0.0902*** |  | -0.0435*** |  | -0.1269*** |  | |
|  | (0.017) |  | (0.024) |  | (0.024) |  | (0.0164) |  | (0.0228) |  | (0.0228) |  | |
| Coresid. Careg. |  | -0.4322*** |  | -0.3603*** |  | -0.4860*** |  | -0.4696*** |  | -0.3863*** |  | -0.5332*** | |
|  |  | (0.033) |  | (0.048) |  | (0.046) |  | (0.0308) |  | ((0.0434) |  | (0.0418) | |
| Non-coresid.Careg. |  | -0.0224*** |  | -0.0174*** |  | -0.0273*** |  | -0.0234*** |  | -0.0180*** |  | -0.0288*** | |
|  |  | (0.008) |  | (0.011) |  | (0.002) |  | (0.0079) |  | (0.0108) |  | (0.0020) | |
| Current income | 0.0247*** | 0.0262*** | 0.0331*** | 0.0346*** | 0.0247*** | 0.0264*** | 0.0259*** | 0.0276*** | 0.0353*** | 0.0370*** | 0.0259*** | 0.0278*** | |
|  | (0.007) | (0.007) | (0.012) | (0.012) | (0.010) | (0.010) | (0.0069) | (0.0069) | (0.0116) | (0.0116) | (0.0097) | (0.0097) | |
| Long-term income | 0.0904*** | 0.0846*** | 0.1002*** | 0.0968*** | 0.0896*** | 0.0848*** | 0.1067*** | 0.0989*** | 0.1203*** | 0.1155*** | 0.1057*** | 0.0992*** | |
|  | (0.010) | (0.010) | (0.015) | (0.015) | (0.015) | (0.015) | (0.0097) | (0.0097) | (0.0143) | (0.0143) | (0.0143) | (0.0143) | |
| **Austria** |  |  |  |  |  |  |  |  |  |  |  |  | |
| Caregiver | -0.1739*** |  | -0.0786*** |  | -0.1293*** |  | -0.2344*** |  | -0.0910*** |  | -0.1627*** |  | |
|  | (0.024) |  | (0.035) |  | (0.025) |  | (0.0228) |  | (0.0326) |  | (0.0238) |  | |
| Coresid. Careg. |  | -0.3024*** |  | -0.0971*** |  | -0.3768*** |  | -0.3207*** |  | -0.0990*** |  | -0.4052*** | |
|  |  | (0.105) |  | (0.021) |  | (0.132) |  | (0.0830) |  | (0.0201) |  | (0.0972) | |
| Non-coresid.Careg. |  | -0.0677*** |  | -0.0556*** |  | -0.1163*** |  | -0.0769*** |  | -0.0618*** |  | -0.1434*** | |
|  |  | (0.029) |  | (0.011) |  | (0.032) |  | (0.0273) |  | (0.0108) |  | (0.0300) | |
| Current income | 0.1217*** | 0.1123*** | 0.2002*** | 0.1103*** | 0.1123*** | 0.1083 *** | 0.1513*** | 0.1375*** | 0.2804*** | 0.1346*** | 0.1375*** | 0.1318*** | |
|  | (0.037) | (0.037) | (0.128) | (0.128) | (0.036) | (0.036) | (0.0329) | (0.0329) | (0.0788) | (0.0788) | (0.0321) | (0.0321) | |
| Long-term income | 0.1881*** | 0.1804*** | 0.2133** | 0.2116** | 0.1353*** | 0.1494*** | 0.2589*** | 0.2455*** | 0.3043*** | 0.3011*** | 0.1719*** | 0.1940*** | |
|  | (0.023) | (0.023) | (0.029) | (0.029) | (0.007) | (0.007) | (0.0214) | (0.0214) | (0.0265) | (0.0265) | (0.0069) | (0.0069) | |
| **Germany** |  |  |  |  |  |  |  |  |  |  |  |  | |
| Caregiver | -0.1304** |  | -0.1445*** |  | -0.1320* |  | -0.1644*** |  | -0.1863*** |  | -0.1668*** |  | |
|  | (0.057) |  | (0.054) |  | (0.077) |  | (0.0505) |  | (0.0482) |  | (0.0651) |  | |
| Coresid. Careg. |  | -0.5621*** |  | -0.7671*** |  | -0.4369*** |  | -0.6253*** |  | -0.8848*** |  | -0.4751*** | |
|  |  | (0.111) |  | (0.173) |  | (0.146) |  | (0.0864) |  | (0.1131) |  | (0.1034) | |
| Non-coresid.Careg. |  | -0.0632*** |  | -0.0410*** |  | -0.0932*** |  | -0.0712*** |  | -0.0444*** |  | -0.1106*** | |
|  |  | (0.029) |  | (0.017) |  | (0.020) |  | (0.0273) |  | (0.0164) |  | (0.0192) | |
| Current income | 0.0675** | 0.0607* | 0.1546*** | 0.1497*** | 0.0156*** | 0.0115** | 0.0766*** | 0.0681*** | 0.2024*** | 0.1945*** | 0.0161*** | 0.0118*** | |
|  | (0.032) | (0.032) | (0.055) | (0.055) | (0.005) | (0.005) | (0.0289) | (0.0289) | (0.0459) | (0.0459) | (0.0049) | (0.0049) | |
| Long-term income | 0.1026*** | 0.1047*** | 0.1120*** | 0.1140*** | 0.0940*** | 0.0845*** | 0.1237*** | 0.1266*** | 0.1371*** | 0.1400*** | 0.1117*** | 0.0988*** | |
|  | (0.026) | (0.026) | (0.045) | (0.004) | (0.031) | (0.031) | (0.0240) | (0.0240) | (0.0389) | (0.0040) | (0.0281) | (0.0281) | |
| **Sweden** |  |  |  |  |  |  |  |  |  |  |  |  | |
| Caregiver | -0.1223** |  | -0.1205* |  | -0.1148*** |  | -0.1522*** |  | -0.1495*** |  | -0.1412*** |  | |
|  | (0.051) |  | (0.071) |  | (0.022) |  | (0.0458) |  | (0.0609) |  | (0.0210) |  | |
| Coresid. Careg. |  | -0.5423*** |  | -0.4251** |  | -0.6209*** |  | -0.6011*** |  | -0.4612*** |  | -0.6980*** | |
|  |  | (0.130) |  | (0.194) |  | (0.174) |  | (0.0962) |  | (0.1187) |  | (0.1134) | |
| Non-coresid.Careg. |  | -0.0724*** |  | -0.0699*** |  | -0.0588*** |  | -0.0829*** |  | -0.0797*** |  | -0.0657*** | |
|  |  | (0.022) |  | (0.023) |  | (0.013) |  | (0.0210) |  | (0.0219) |  | (0.0127) | |
| Current income | 0.0415*** | 0.0317*** | 0.0810*** | 0.0686*** | 0.0095*** | 0.0053*** | 0.0449*** | 0.0337*** | 0.0941*** | 0.0780*** | 0.0097*** | 0.0054*** | |
|  | (0.009) | (0.009) | (0.014) | (0.014) | (0.002) | (0.002) | (0.0088) | (0.0088) | (0.0134) | (0.0134) | (0.0020) | (0.0018) | |
| Long-term income | 0.1599*** | 0.1571*** | 0.2137*** | 0.2089*** | 0.0539*** | 0.0462*** | 0.2110*** | 0.2065*** | 0.3050*** | 0.2962*** | 0.0597*** | 0.0505*** | |
|  | (0.043) | (0.043) | (0.061) | (0.061) | (0.018) | (0.017) | (0.0375) | (0.0375) | (0.0498) | (0.0498) | (0.0170) | (0.0161) | |
| **Spain** |  |  |  |  |  |  |  |  |  |  |  |  | |
| Caregiver | -0.3119*** |  | -0.2460*** |  | -0.2646** |  | -0.5065*** |  | -0.3670*** |  | -0.4046*** |  | |
|  | (0.082) |  | (0.107) |  | (0.126) |  | (0.0686) |  | (0.0841) |  | (0.0942) |  | |
| Coresid. Careg. |  | -0.5458*** |  | -0.6613*** |  | -0.4198** |  | -0.6054 |  | -0.7488*** |  | -0.4550*** | |
|  |  | (0.104) |  | (0.131) |  | (0.166) |  | (0.0824) |  | (0.0967) |  | (0.110)9 | |
| Non-coresid.Careg. |  | -0.0070 |  | -0.1852 |  | -0.1114 |  | -0.0071*** |  | -0.2538*** |  | -0.1362*** | |
|  |  | (0.111) |  | (0.156) |  | (0.159) |  | (0.0864) |  | (0.1073) |  | (0.1084) | |
| Current income | 0.0618** | 0.0673** | 0.0752*** | 0.0714*** | 0.0476*** | 0.0440*** | 0.0694*** | 0.0764*** | 0.0865*** | 0.0816*** | 0.0521*** | 0.0479*** | |
|  | (0.027) | (0.027) | (0.032) | (0.032) | (0.017) | (0.017) | (0.0248) | (0.0248) | (0.0289) | (0.0289) | (0.0161) | (0.0161) | |
| Long-term income | 0.0750** | 0.0703*** | 0.0815*** | 0.0961*** | 0.0522*** | 0.0578*** | 0.0863*** | 0.0802*** | 0.0948*** | 0.1146*** | 0.0576*** | 0.0645*** | |
|  | (0.023) | (0.022) | (0.030) | (0.030) | (0.017) | (0.017) | (0.0214) | (0.0205) | (0.0273) | (0.0273) | (0.0161) | (0.0161) | |
| **Italy** |  |  |  |  |  |  |  |  |  |  |  |  | |
| Caregiver | -0.2401*** |  | -0.1057** |  | -0.2948*** |  | -0.3554*** |  | -0.1280*** |  | -0.4686*** |  | |
|  | (0.056) |  | (0.078) |  | (0.081) |  | (0.0497) |  | (0.0658) |  | (0.0679) |  | |
| Coresid. Careg. |  | -0.4750*** |  | -0.3700*** |  | -0.5299*** |  | -0.5201*** |  | -0.3974*** |  | -0.5861*** | |
|  |  | (0.082) |  | (0.122) |  | (0.112) |  | (0.0686) |  | (0.0922) |  | (0.0869) | |
| Non-coresid.Careg. |  | -0.1655*** |  | -0.0185*** |  | -0.1322 |  | -0.2203*** |  | -0.0192*** |  | -0.1672*** | |
|  |  | (0.025) |  | (0.068) |  | (0.096) |  | (0.0238) |  | (0.0588) |  | (0.0776) | |
| Current income | 0.0672*** | 0.0605*** | 0.0659**** | 0.0603**** | 0.0598*** | 0.0613*** | 0.0762*** | 0.0678*** | 0.0746*** | 0.0676*** | 0.0670*** | 0.0688*** | |
|  | (0.030) | (0.029) | (0.025) | (0.025) | (0.018) | (0.018) | (0.0273) | (0.0265) | (0.0231) | (0.0231) | (0.0170) | (0.0170) | |
| Long-term income | 0.0831*** | 0.0822*** | 0.0653*** | 0.0678*** | 0.0935*** | 0.0911*** | 0.0969*** | 0.0957*** | 0.0738*** | 0.0770*** | 0.1110*** | 0.1077*** | |
|  | (0.021) | (0.021) | (0.026) | (0.026) | (0.027) | (0.027) | (0.0197) | (0.0197) | (0.0240) | (0.0240 | (0.0248) | (0.0248) | |
| **France** |  |  |  |  |  |  |  |  |  |  |  |  | |
| Caregiver | -0.1882*** |  | -0.2284** |  | -0.1614*** |  | -0.2590*** |  | -0.3327*** |  | -0.2135*** |  | |
|  | (0.053) |  | (0.075) |  | (0.054) |  | (0.0474) |  | (0.0638) |  | (0.0482) |  | |
| Coresid. Careg. |  | -0.2575** |  | -0.3317** |  | -0.2163*** |  | -0.2708*** |  | -0.3537*** |  | -0.2257*** | |
|  |  | (0.103) |  | (0.137) |  | (0.091) |  | (0.0818) |  | (0.0995) |  | (0.0744) | |
| Non-coresid.Careg. |  | -0.1335** |  | -0.1400*** |  | -0.1158*** |  | -0.1691*** |  | -0.1792*** |  | -0.1426*** | |
|  |  | (0.056) |  | (0.060) |  | (0.048) |  | (0.0497) |  | (0.0528) |  | (0.0434) | |
| Current income | 0.0894*** | 0.0882*** | 0.1735*** | 0.1666*** | 0.0596** | 0.0574** | 0.1054*** | 0.1038*** | 0.2337*** | 0.2221*** | 0.0667*** | 0.0640*** | |
|  | (0.024) | (0.024) | (0.049) | (0.048) | (0.029) | (0.029) | (0.0223) | (0.0223) | (0.0418) | (0.0411) | (0.0265) | (0.0265) | |
| Long-term income | 0.1132*** | 0.1124*** | 0.0650*** | 0.0677*** | 0.1430*** | 0.1417*** | 0.1388*** | 0.1377*** | 0.0735*** | 0.0769*** | 0.1839*** | 0.1819*** | |
|  | (0.034) | (0.034) | (0.010) | (0.010) | (0.048) | (0.048) | (0.0305) | (0.0305) | (0.0097) | (0.0097) | (0.0411) | (0.0411) | |
| **Denmark** |  |  |  |  |  |  |  |  |  |  |  |  | |
| Caregiver | -0.1300*** |  | -0.0325*** |  | -0.1866*** |  | -0.1638*** |  | -0.0346*** |  | -0.2562*** |  | |
|  | (0.018) |  | (0.006) |  | (0.012) |  | (0.0174) |  | (0.0059) |  | (0.0117) |  | |
| Coresid. Careg. |  | -0.1604*** |  | -0.0556*** |  | -0.2735** |  | -0.1655*** |  | -0.0562*** |  | -0.2885*** | |
|  |  | (0.070) |  | (0.027) |  | (0.108) |  | (0.0602) |  | (0.0255) |  | (0.0847) | |
| Non-coresid.Careg. |  | -0.0931** |  | -0.0214*** |  | -0.1098** |  | -0.1104*** |  | -0.0223*** |  | -0.1339*** | |
|  |  | (0.039) |  | (0.010) |  | (0.033) |  | (0.0360) |  | (0.0098) |  | (0.0308) | |
| Current income | 0.0361*** | 0.0387*** | 0.0045*** | 0.0029*** | 0.0519*** | 0.0538 *** | 0.0387*** | 0.0417*** | 0.0045*** | 0.0029*** | 0.0573*** | 0.0596*** | |
|  | (0.012) | (0.012) | (0.011) | (0.010) | (0.014) | (0.014) | (0.0116) | (0.0116) | v0.0106) | (0.0097) | (0.0134) | (0.0134) | |
| Long-term income | 0.1093*** | 0.1006*** | 0.1555*** | 0.1556*** | 0.0540*** | 0.0526*** | 0.1332*** | 0.1208*** | 0.2039*** | 0.2040*** | 0.0598*** | 0.0581*** | |
|  | (0.029) | (0.029) | (0.041) | (0.041) | (0.010) | (0.010) | (0.0265) | (0.0265) | (0.0360) | (0.0360) | (0.0097) | (0.0097) | |
| **Switzerland** |  |  |  |  |  |  |  |  |  |  |  |  | |
| Caregiver | -0.2575*** |  | -0.0939*** |  | -0.5910*** |  | -0.3901*** |  | -0.1115*** |  | -1.2896*** |  | |
|  | (0.049) |  | (0.024) |  | (0.066) |  | (0.0442) |  | (0.0228) |  | (0.0573) |  | |
| Coresid. Careg. |  | -0.4928*** |  | -0.1193*** |  | -0.6374*** |  | -0.5414*** |  | -0.1221*** |  | -0.7187*** | |
|  |  | (0.135) |  | (0.025) |  | (0.178) |  | (0.0986) |  | (0.0238) |  | (0.1146) | |
| Non-coresid.Careg. |  | -0.1135** |  | -0.0475*** |  | -0.3041 *** |  | -0.1393*** |  | -0.0520*** |  | -0.4891*** | |
|  |  | (0.051) |  | (0.010) |  | (0.028) |  | (0.0458) |  | (0.0098) |  | (0.0264) | |
| Current income | 0.0466*** | 0.0459*** | 0.1136*** | 0.1138*** | 0.0070*** | 0.0060*** | 0.0509*** | 0.0501*** | 0.1394*** | 0.1397*** | 0.0071*** | 0.0061*** | |
|  | (0.016) | (0.016) | (0.040) | (0.040) | (0.028) | (0.028) | (0.0152) | (0.0152) | (0.0352) | (0.0352) | (0.0256) | (0.0256) | |
| Long-term income | 0.1266*** | 0.1264*** | 0.1463*** | 0.1461*** | 0.0591** | 0.0537* | 0.1587*** | 0.1584*** | 0.1891*** | 0.1888*** | 0.0661*** | 0.0595*** | |
|  | (0.023) | (0.023) | (0.035) | (0.035) | (0.029) | (0.029) | (0.0214 | (0.0214) | (0.0313) | (0.0313) | (0.0265) | (0.0265) | |
| **Belgium** |  |  |  |  |  |  |  |  |  |  |  |  | |
| Caregiver | -0.0741*** |  | -0.0517*** |  | -0.1113** |  | -0.0851*** |  | -0.0570*** |  | -0.1361*** |  | |
|  | (0.017) |  | (0.011) |  | (0.054) |  | (0.0164) |  | (0.0108) |  | (0.0482) |  | |
| Coresid. Careg. |  | -0.0302*** |  | -0.0614*** |  | -0.1487*** |  | -0.0304*** |  | -0.0622*** |  | -0.1531*** | |
|  |  | (0.010) |  | (0.023) |  | (0.055) |  | (0.0098) |  | (0.0219) |  | (0.0490) | |
| Non-coresid.Careg. |  | -0.1047*** |  | -0.0341*** |  | -0.0173*** |  | -0.1266*** |  | -0.0364*** |  | -0.0179*** | |
|  |  | (0.038) |  | (0.009) |  | (0.006) |  | (0.0351) |  | (0.0088) |  | (0.0059) | |
| Current income | 0.0403** | 0.0404** | 0.0515*** | 0.0510*** | 0.0328*** | 0.0331*** | 0.0435*** | 0.0437*** | 0.0568*** | 0.0562*** | 0.0350*** | 0.0353*** | |
|  | (0.019) | (0.019) | (0.019) | (0.019) | (0.007) | (0.007) | (0.0179) | (0.0179) | (0.0179) | (0.0179) | (0.0069) | (0.0069) | |
| Long-term income | 0.0929*** | 0.0932*** | 0.0676*** | 0.0678*** | 0.1270*** | 0.1269*** | 0.1102*** | 0.1106*** | 0.0767*** | 0.0770*** | 0.1593*** | 0.1591*** | |
|  | (0.013) | (0.013) | (0.022) | (0.022) | (0.017) | (0.017) | (0.0125) | (0.0125) | (0.0205) | (0.0205) | (0.0161) | (0.0161) | |
| **Czech Republic** |  |  |  |  |  |  |  |  |  |  |  |  | |
| Caregiver | -0.2078*** |  | -0.0661*** |  | -0.2698*** |  | -0.2942*** |  | -0.0748*** |  | -0.3426*** |  | |
|  | (0.063) |  | (0.002) |  | (0.082) |  | (0.0551) |  | (0.0020) |  | (0.0753) |  | |
| Coresid. Careg. |  | -0.4536*** |  | -0.0817*** |  | -0.6355*** |  | -0.4948*** |  | -0.0830*** |  | -0.7163*** | |
|  |  | (0.112) |  | (0.012) |  | (0.142) |  | (0.0869) |  | (0.0117) |  | (0.1017) | |
| Non-coresid.Careg. |  | -0.0703*** |  | -0.0614*** |  | -0.0877*** |  | -0.0802*** |  | -0.0689*** |  | -0.1031*** | |
|  | (0.041) | (0.041) | (0.014) | (0.014) | (0.054) | (0.054) |  | (0.0376) |  | (0.0136) |  | (0.0482) | |
| Current income | 0.0957** | 0.0912** | 0.0338** | 0.0353** | 0.1137** | 0.1097** | 0.1140*** | 0.1078*** | 0.0361*** | 0.0378*** | 0.1396*** | 0.1338*** | |
|  | (0.041) | (0.041) | (0.014) | (0.014) | (0.054) | (0.054) | (0.0360) | (0.0360) | (0.0134) | (0.0134) | (0.0453) | (0.0453) | |
| Long-term income | 0.2404*** | 0.2471*** | 0.1781** | 0.1780** | 0.2927*** | 0.3052*** | 0.3560*** | 0.3692*** | 0.2415*** | 0.2414*** | 0.4640*** | 0.4915*** | |
|  | (0.051) | (0.051) | (0.089) | (0.089) | (0.064) | (0.064) | (0.0432) | 0.0432) | (0.0652) | (0.0652) | (0.0517) | (0.0517) | |

Note: The left part of the table shows the estimated coefficients of the FEF model without using instrumental variables (Table 1 for the total sample and Table B2 for each of the countries). The right side shows the results of the FEF without using instrumental variables (explanatory variables in the regressions for life satisfaction: same as in Table B2; explanator variables in the regressions for long-term individual income (2007-2020; PPP2020): same as in Table B2).Caregiving is instrumented using the following variables: being the eldest child, being the youngest child, being single child, number of brothers and number of sisters.

Current individual income (PPP2020) is instrumented using income from partner and income from other household members.

Long-term individual income (s007-2020; PPP2020) is instrumented using long-term income from partner y long-term income from other household members.

Standard errors are adjusted for clustering on year level.

***denotes significance at the 99% level, **at the 95% level, *at the 90% level.

**Table B5. Effect of long-term care expenditure over life satisfaction**

|  | **All sample** | | | **Noncaregivers** | | | **Informal caregivers** | | |
| --- | --- | --- | --- | --- | --- | --- | --- | --- | --- |
|  | **Total** | **Men** | **Women** | **Total** | **Men** | **Women** | **Total** | **Men** | **Women** |
| LTC expenditure (1000€; PPP2020) | 0.04686*** | 0.02245*** | 0.06452*** | 0.04126*** | 0.01658* | 0.05911*** | 0.06451*** | 0.04175*** | 0.08096*** |
|  | (0.005) | (0.008) | (0.007) | (0.007) | (0.010) | (0.009) | (0.009) | (0.013) | (0.013) |
| N | 540.800 | 223.700 | 317.100 | 366.300 | 148.300 | 218 | 174.500 | 75.400 | 99.100 |
| R2 | 0.14884 | 0.13810 | 0.15799 | 0.14475 | 0.13324 | 0.15693 | 0.17304 | 0.17443 | 0.18072 |
| F | 5.235.208 | 2.091.499 | 3.480.108 | 3.426.340 | 1.324.669 | 2.367.322 | 2.006.500 | 914.710 | 1.262.542 |
| p | 0.00000 | 0.00000 | 0.00000 | 0.00000 | 0.00000 | 0.00000 | 0.00000 | 0.00000 | 0.00000 |

Note: Explanatory variables: age, marital status, level of education, size of municipality, Charlston Comorbidity Index, relation with economic activity, current health status, log (Wealth adjusted by household size PPP2020), period during which were happier than during the rest of his/her life, age when happiness period started, length of happiness period (years),period during which were under more stress than during the rest of his/her life, age when stress period started, length of stress period (years),period during which health was poorer compared to the rest of his/her your life ,age when poor health period started, length of poor health period (years),period during which there was a distinct financial hardship ,age when financial hardship started, length of financial hardship period (years),period during which suffered from hunger ,age when hunger period started, length of hunger period (years),ever been the victim of such persecution or discrimination, health during childhood, during your childhood. ever in hospital at least one month,a physical injury that has led to any permanent handicap, number of books at home at age 10, performance in maths at the age of 10, performance in language at the age of 10, lived with biological father, lived with biological mother, lived with stepfather, lived with stepmother, fixed bath, cold running water supply, hot running water supply, inside toilet, central heating, lived in a children’s home, been fostered with another family, evacuated or relocated during a war, lived in a prisoner of war camp, lived in a prison, lived in a labor camp, lived in a concentration camp, been an inpatient in a tuberculosis institution, stayed in a psychiatric hospital, been homeless for 1 month or more, born in other country, country and year fixed effects. ***denotes significance at the 99% level, **at the 95% level, *at the 90% level.

**Table B6. Classification of long-term care regimes**

|  | Demand for care | Provision of informal care | Provision of formal care | **Countries** |
| --- | --- | --- | --- | --- |
| Standard care mix | High | Medium/low | Medium | **Belgium, Germany, Austria, France, Switzerland** |
| Universal Nordic | Medium | Low | High | **Sweden** |
| Family based | High | High | Low | **Spain, Italy** |
| Transition | Medium | High | Medium/low | **Czech Republic** |

Source: own work using Ilinca et al., (2015, 2022), Jiménez-Martín and Vilaplana-Prieto (2015).

Low, intermediate and high spending levels correspond to the terciles of long-term care spending as a percentage of GDP.

**Appendix C**

**Figure C1. Difference between short-term and long-term compensating surplus using Bartik instrument and using the initial instruments for short-term and long-term individual income**

|  |
| --- |

Note: Blue bars: individual long-term CS using Bartik instrument minus individual long-term CS using initial instruments for income. Red bars: individual short-term CS using Bartik instrument minus individual short-term CS using initial instruments for income

**Table C1. Individual short and long-term compensating surplus using Bartik instrument**

|  | Total | | | Men | | | Women | | |
| --- | --- | --- | --- | --- | --- | --- | --- | --- | --- |
|  | Caregiver | Coresid.  caregiver | No coresid  caregiver | Caregiver | Coresid.  caregiver | No coresid  caregiver | Caregiver | Coresid.  caregiver | No coresid  caregiver |
| **Individual short-term CS (Euros PPP 2020)** |  |  |  |  |  |  |  |  |  |
| Austria | **13,439** | **35,816** | **8,350** | **21,649** | **66,354** | **11,236** | **11,559** | **28,542** | **7,687** |
| Belgium | **14,918** | **50,178** | **8,656** | **17,748** | **56,026** | **10,762** | **14,408** | **48,923** | **8,276** |
| Czechia | **12,456** | **28,447** | **7,966** | **20,252** | **47,458** | **12,433** | **10,258** | **22,893** | **6,705** |
| Denmark | **8,878** | **46,358** | **5,065** | **11,884** | **67,761** | **6,081** | **8,567** | **43,998** | **4,960** |
| France | **17,444** | **50,301** | **10,356** | **21,427** | **55,664** | **13,810** | **16,574** | **48,887** | **9,601** |
| Germany | **9,253** | **25,676** | **5,727** | **11,767** | **34,788** | **6,748** | **8,709** | **23,620** | **5,506** |
| Italy | **14,081** | **18,865** | **11,197** | **19,974** | **27,046** | **15,558** | **10,531** | **13,785** | **8,566** |
| Spain | **28,594** | **25,114** | **32,960** | **33,661** | **28,606** | **40,013** | **22,294** | **20,765** | **24,209** |
| Sweden | **7,251** | **37,172** | **4,196** | **11,810** | **72,104** | **5,547** | **6,780** | **33,423** | **4,056** |
| Switzerland | **14,241** | **34,941** | **12,027** | **18,348** | **23,780** | **17,742** | **13,798** | **36,128** | **11,412** |
| **Individual long-term CS (2007-2020)** |  |  |  |  |  |  |  |  |  |
| Austria | **213,831** | **565,428** | **133,051** | **343,655** | **1,057,883** | **178,904** | **184,022** | **452,194** | **122,515** |
| Belgium | **237,273** | **788,536** | **137,915** | **282,047** | **893,483** | **171,366** | **229,195** | **770,693** | **131,879** |
| Czechia | **198,244** | **450,164** | **126,957** | **321,617** | **757,035** | **197,889** | **163,369** | **363,272** | **106,896** |
| Denmark | **141,444** | **729,392** | **80,788** | **189,185** | **1,080,263** | **96,963** | **136,511** | **694,053** | **79,116** |
| France | **277,250** | **790,445** | **164,934** | **340,159** | **887,729** | **219,710** | **263,476** | **770,136** | **152,940** |
| Germany | **147,403** | **406,682** | **91,333** | **187,322** | **555,137** | **107,587** | **138,770** | **374,740** | **87,817** |
| Italy | **224,014** | **299,465** | **178,275** | **317,221** | **431,684** | **247,405** | **167,700** | **219,320** | **136,497** |
| Spain | **450,611** | **396,412** | **518,369** | **529,221** | **450,806** | **627,257** | **352,367** | **328,428** | **382,292** |
| Sweden | **115,580** | **586,575** | **66,943** | **188,005** | **1,149,363** | **88,466** | **108,094** | **528,803** | **64,718** |
| Switzerland | **226,542** | **551,761** | **191,454** | **291,541** | **379,597** | **281,955** | **219,533** | **571,159** | **181,697** |

Note: CS long-term: monetary change necessary to hold utility constant for caregivers in the period 2007-2020.

**Table C2. Effect of instruments for informal care on life satisfaction**

|  | **All sample** | | |
| --- | --- | --- | --- |
|  | **Total** | **Men** | **Women** |
| Number of brothers | 0.026* | 0.012 | 0.034* |
|  | (0.014) | (0.009) | (0.0018) |
| Number of sisters | -0.003 | 0.011 | -0.012 |
|  | (0.008) | (0.012) | (0.010) |
| Single child | -0.055 | -0.087 | -0.027 |
|  | (0.048) | (0.067) | (0.068) |
| Eldest child | 0.047 | 0.020 | 0.060 |
|  | (0.051) | (0.073) | (0.070) |
| Youngest child | -0.043 | 0.012 | -0.067 |
|  | (0.053) | (0.078) | (0.071) |
| N | 42,248 | 17,777 | 24,471 |
| R2 | 0.201 | 0.190 | 0.213 |
| F | 101.231 | 40.353 | 63.588 |
| p | 0.000 | 0.000 | 0.000 |
|  | **Informal caregivers** | | |
|  | **Total** | **Men** | **Women** |
|  |  |  |  |
| Number of brothers | 0.018 | -0.007 | 0.013 |
|  | (0.012) | (0.014) | (0.014) |
| Number of sisters | 0.018 | 0.018 | 0.018 |
|  | (0.013) | (0.021) | (0.017) |
| Single child | 0.032 | -0.017 | 0.084 |
|  | (0.078) | (0.103) | (0.116) |
| Eldest child | 0.022 | 0.034 | -0.002 |
|  | (0.075) | (0.111) | (0.101) |
| Youngest child | -0.101 | 0.006 | -0.145 |
|  | (0.080) | (0.125) | (0.104) |
| N | 14,329 | 5,893 | 8,436 |
| R2 | 0.232 | 0.229 | 0.246 |
| F | 40.886 | 16.698 | 26.371 |
| p | 0.000 | 0.000 | 0.000 |
|  | **Non-caregivers** | | |
|  | **Total** | **Men** | **Women** |
| Number of brothers | 0.012* | 0.024 | 0.024** |
|  | (0.007) | (0.015) | (0.013) |
| Number of sisters | -0.015 | 0.007 | -0.027** |
|  | (0.010) | (0.014) | (0.013) |
| Single child | -0.098 | -0.123 | -0.079 |
|  | (0.061) | (0.087) | (0.084) |
| Eldest child | 0.072 | 0.015 | 0.120 |
|  | (0.068) | (0.097) | (0.095) |
| Youngest child | 0.002 | 0.020 | 0.002 |
|  | (0.070) | (0.101) | (0.096) |
| N | 27,919 | 11,884 | 16,035 |
| R2 | 0.194 | 0.182 | 0.210 |
| F | 63.654 | 25.378 | 40.658 |
| p | 0.000 | 0.000 | 0.000 |

Note: Explanatory variables: age, marital status, level of education, size of municipality, Charlston Comorbidity Index, relation with economic activity, current health status, log (Wealth adjusted by household size PPP2020), period during which were happier than during the rest of his/her life, age when happiness period started, length of happiness period (years),period during which were under more stress than during the rest of his/her life ,age when stress period started, length of stress period (years),period during which health was poorer compared to the rest of his/her your life ,age when poor health period started, length of poor health period (years),period during which there was a distinct financial hardship ,age when financial hardship started, length of financial hardship period (years),period during which suffered from hunger ,age when hunger period started, length of hunger period (years),ever been the victim of such persecution or discrimination, health during childhood, during your childhood. ever in hospital at least one month,a physical injury that has led to any permanent handicap, number of books at home at age 10, performance in maths at the age of 10, performance in language at the age of 10,lived with biological father, lived with biological mother, lived with stepfather, lived with stepmother, fixed bath, cold running water supply, hot running water supply, inside toilet, central heating, lived in a children’s home, been fostered with another family, evacuated or relocated during a war, lived in a prisoner of war camp, lived in a prison, lived in a labor camp, lived in a concentration camp, been an inpatient in a tuberculosis institution, stayed in a psychiatric hospital, been homeless for 1 month or more, born in other country, country and year fixed effects. ***denotes significance at the 99% level, **at the 95% level, *at the 90% level.

**Table C3. Effect of instruments for income instruments on life satisfaction**

|  | **All sample** | | |
| --- | --- | --- | --- |
|  | **Total** | **Men** | **Women** |
| Log(income partner PPP2020) | 0.036 | 0.037 | 0.021 |
|  | (0.036) | (0.064) | (0.049) |
| Log(income other household members PPP2020) | 0.024 | 0.079 | 0.085 |
|  | (0.037) | (0.058) | (0.052) |
| N | 42,248 | 17,777 | 24,471 |
| R2 | 0.366 | 0.470 | 0.506 |
| F | 3,509.23 | 1,923.73 | 3,167.75 |
| p | 0.000 | 0.000 | 0.000 |
|  | **Informal caregivers** | | |
|  | **Total** | **Men** | **Women** |
|  |  |  |  |
| Log(income partner PPP2020) | 0.022 | 0.010 | 0.038 |
|  | (0.056) | (0.114) | (0.091) |
| Log(income other household members PPP2020) | 0.093 | 0.092 | 0.097 |
|  | (0.063) | (0.093) | (0.087) |
| N | 14,329 | 5,893 | 8,436 |
| R2 | 0.501 | 0.746 | 0.697 |
| F | 2,565.49 | 2,102.99 | 2,488.03 |
| p | 0.000 | 0.000 | 0.000 |
|  | **Noncaregivers** | | |
|  | **Total** | **Men** | **Women** |
| Log(income partner PPP2020) | 0.044 | 0.094 | 0.024 |
|  | (0.065) | (0.110) | (0.077) |
| Log(income other household members PPP2020) | 0.088 | 0.108 | 0.081 |
|  | (0.079) | (0.105) | (0.089) |
| N | 27,919 | 11,884 | 16,035 |
| R2 | 0.480 | 0.755 | 0.644 |
| F | 2,340.71 | 1,831.54 | 1,990.34 |
| p | 0.000 | 0.000 | 0.000 |

Note: Explanatory variables: age, marital status, level of education, size of municipality, Charlston Comorbidity Index, relation with economic activity, current health status, log (Wealth adjusted by household size PPP2020), period during which were happier than during the rest of his/her life, age when happiness period started, length of happiness period (years),period during which were under more stress than during the rest of his/her life ,age when stress period started, length of stress period (years),period during which health was poorer compared to the rest of his/her your life ,age when poor health period started, length of poor health period (years),period during which there was a distinct financial hardship ,age when financial hardship started, length of financial hardship period (years),period during which suffered from hunger ,age when hunger period started, length of hunger period (years),ever been the victim of such persecution or discrimination, health during childhood, during your childhood. ever in hospital at least one month,a physical injury that has led to any permanent handicap, number of books at home age age 10, performance in maths at the age of 10, performance in language at the age of 10,lived with biological father, lived with biological mother, lived with stepfather, lived with stepmother, fixed bath, cold running water supply, hot running water supply, inside toilet, central heating, lived in a children’s home, been fostered with another family, evacuated or relocated during a war, lived in a prisoner of war camp, lived in a prison, lived in a labor camp, lived in a concentration camp, been an inpatient in a tuberculosis institution, stayed in a psychiatric hospital, been homeless for 1 month or more, born in other country, country and year fixed effects. ***denotes significance at the 99% level, **at the 95% level, *at the 90% level.

**Appendix D**

The aim of this annex is to contrast the estimates of the compensating surplus of informal care using an alternative source of information, the European Quality of Life Survey.

The European Quality of Life Survey (EQLS) is a monitoring tool to capture quality of life in multiple dimensions. Carried out in 2003, 2007, 2011 and 2016, the EQLS documents the living conditions and social situation of European citizens. It includes subjective and objective measures, reported attitudes and preferences, as well as resources and experiences. The fourth EQLS was carried out from September 2016 to March 2017 in all EU Member States and the five candidate countries (Albania, the former Yugoslav Republic of Macedonia, Montenegro, Serbia and Turkey). It was coordinated by Kantar Public, with local partners interviewing a total of nearly 37,000 people in the 33 different countries, with sample sizes ranging from 1,000 to 2,000 per country. High standards of quality assurance were applied to all stages of the survey’s implementation, and include an external quality assessment (Eurofund, 2017).

The main disadvantages of the EQLS are that: (i) it does not allow to distinguish between coresident and non-coresident informal caregivers; (ii) we cannot estimate the long-term income and (iii) Switzerland has not been included in the sample.

However, we consider that from the EQLS we can estimate the compensating surplus for all caregivers and also differentiating by age (under 50 and 50 and over) and sex. In this way we can perform a double comparison: (i) comparison of the compensating surplus for caregivers (men and women) aged 50 and over between SHARE and EQLS; (ii) comparison of the compensating surplus for the whole population with estimates of the value of informal care using the opportunity cost method (Peña-Longobardo et al., 2022).

To estimate the compensating surplus associated with informal care with the EQLS, a process similar to that used for SHARE has been followed, with the exception that we have a single cross-section and not a panel data sample. It should be noted that the instruments used for estimation with instrumental variables are different. For "informal caregiver" we have used the number of daughters and sons, and for "income" we have used the relationship with the economic activity of the partner and the number of working hours of the partner.

Table D1 shows the description of the EQLS(2016) sample for the nine countries that match those analysed in SHARE. Table D2 shows the descriptive statistics and Table D3 describes the characteristics of caregivers (by age and sex), and compares the percentage of caregivers aged 50 and over in SHARE and EQLS. There is a high degree of consistency between both surveys.

**Table D1. Description of the sample**

|  | Total | Age | | Men | Women | Percentage of informal caregivers aged 50 and older | | |
| --- | --- | --- | --- | --- | --- | --- | --- | --- |
|  |  | Younger than 50 years | 50+ |  |  | Total | Men | Women |
| Austria | 3,067 | 1,255 | 1,812 | 1,261 | 1,806 | 46.82 | 40.98 | 50.00 |
| Belgium | 3,115 | 1,553 | 1,562 | 1,481 | 1,634 | 58.99 | 57.47 | 60.29 |
| Czechia | 2,914 | 1,549 | 1,365 | 1,257 | 1,657 | 54.50 | 61.33 | 50.00 |
| Denmark | 3,466 | 1,845 | 1,621 | 1,641 | 1,825 | 60.91 | 62.86 | 59.28 |
| France | 4,902 | 2,248 | 2,654 | 2,160 | 2,742 | 48.04 | 49.20 | 47.15 |
| Germany | 6,117 | 3,272 | 2,845 | 2,747 | 3,370 | 55.06 | 54.17 | 55.63 |
| Italy | 4,237 | 2,114 | 2,123 | 1,613 | 2,624 | 59.34 | 63.53 | 57.10 |
| Spain | 2,766 | 1,290 | 1,476 | 1,264 | 1,502 | 56.08 | 50.91 | 59.14 |
| Sweden | 3,658 | 1,952 | 1,706 | 1,776 | 1,882 | 67.94 | 64.43 | 71.08 |

Source: own work using EQLS(2016)

**Table D2. Descriptive statistics**

|  | Total | Non informal caregivers | Informal caregivers |
| --- | --- | --- | --- |
| N | 45,688 | 43,011 | 2,677 |
| Men | 43.59 | 43.73 | 41.35 |
| Women | 56.41 | 56.27 | 58.65 |
| Age | 49.88 | 49.76 | 51.79 |
|  | (17.54) | (17.63) | (15.87) |
| Life satisfaction |  |  |  |
| Very dissatisfied | 1.4 | 1.39 | 1.46 |
| 2 | 1.29 | 1.31 | 0.97 |
| 3 | 2.54 | 2.56 | 2.09 |
| 4 | 3.18 | 3.21 | 2.76 |
| 5 | 8.88 | 8.84 | 9.6 |
| 6 | 9.96 | 9.87 | 11.47 |
| 7 | 19.4 | 19.27 | 21.48 |
| 8 | 26.75 | 26.81 | 25.78 |
| 9 | 14.06 | 14.17 | 12.33 |
| Very satisfied | 12.54 | 12.57 | 12.07 |
| Income (OECD equivalized scale; PPP€ 2020) | 1409.83 | 1668.42 | 1391.49 |
|  | (1654.41) | (1449.59) | (1666.48) |
| Size of municipality |  |  |  |
| Large town | 32.37 | 31.15 | 32.08 |
| Small or middle sized town | 38.08 | 36.02 | 40.41 |
| Rural area or village | 29.55 | 32.83 | 27.51 |
| Education |  |  |  |
| Lower secondary | 30.78 | 31.11 | 26.58 |
| Upper secondary or | 45.17 | 45.55 | 40.34 |
| Tertiary | 24.05 | 23.34 | 33.07 |
| Marital status |  |  |  |
| Never married | 26.32 | 26.79 | 24.84 |
| Married | 49.19 | 47.98 | 52.97 |
| Separated | 3.96 | 3.88 | 4.22 |
| Widowed | 9.9 | 10.58 | 7.77 |
| Divorced | 10.41 | 10.53 | 10.05 |
| Don’t know | 0.02 | 0.01 | 0.04 |
| Refusal | 0.2 | 0.23 | 0.11 |
| Self-reported health status |  |  |  |
| Very good | 22.14 | 22.18 | 21.67 |
| Good | 44.97 | 44.87 | 46.17 |
| Fair | 25.24 | 25.17 | 26.22 |
| Bad | 6.08 | 6.15 | 5.27 |
| Very bad | 1.41 | 1.46 | 0.67 |
| Don't know | 0.08 | 0.09 |  |
| Refusal | 0.08 | 0.09 |  |
| Chronic illness | 27.82 | 27.55 | 31.19 |
| Permannet limitation | 20.75 | 21.05 | 17.37 |
| Number of adults at household |  |  |  |
| 1 | 28.6 | 28.63 | 28.17 |
| 2 | 53.25 | 53.16 | 54.69 |
| 3 | 12.46 | 12.45 | 12.63 |
| 4 | 4.67 | 4.73 | 3.7 |
| 5 | 0.81 | 0.82 | 0.67 |
| 6 | 0.15 | 0.15 | 0.11 |
| 7 or more | 0.06 | 0.06 | 0.04 |
| Number of children at household |  |  |  |
| 1 | 64.19 | 63.93 | 68.23 |
| 2 | 17.04 | 17.18 | 14.79 |
| 3 | 14.28 | 14.34 | 13.39 |
| 4 | 3.51 | 3.58 | 2.45 |
| 5 | 0.73 | 0.73 | 0.85 |
| 6 | 0.20 | 0.19 | 0.25 |
| 7 or more | 0.04 | 0.03 | 0.05 |

Source: own work using EQLS(2016)

**Table D3. Percentage of informal caregivers (EQLS) and comparison with SHARE**

|  | EQLS (2016) | | | | | | | | | SHARE (waves 2, 4, 5, 6, 7 and 8) |
| --- | --- | --- | --- | --- | --- | --- | --- | --- | --- | --- |
|  | Total | | | Age: younger than 50 years | | | Age: 50+ | | | Caregivers  Age: 50+ |
|  | Total | Men | Women | Total | Men | Women | Total | Men | Women |  |
| Austria | 4.06 | 3.46 | 4.50 | 3.66 | 3.59 | 3.70 | 4.66 | 3.28 | 5.74 | 5.82 |
| Belgium | 9.37 | 9.26 | 9.46 | 7.64 | 7.95 | 7.38 | 11.11 | 10.55 | 11.61 | 5.11 |
| Czechia | 4.47 | 4.12 | 4.73 | 4.07 | 3.22 | 4.70 | 4.86 | 5.00 | 4.76 | 7.01 |
| Denmark | 7.60 | 7.54 | 7.64 | 6.67 | 6.40 | 6.90 | 8.33 | 8.43 | 8.25 | 4.31 |
| France | 7.18 | 7.16 | 7.21 | 7.06 | 6.83 | 7.24 | 7.32 | 7.52 | 7.16 | 6.26 |
| Germany | 3.19 | 2.80 | 3.50 | 3.11 | 2.97 | 3.21 | 3.26 | 2.67 | 3.78 | 6.40 |
| Italy | 7.21 | 6.63 | 7.56 | 5.45 | 4.56 | 5.99 | 9.24 | 8.96 | 9.42 | 9.42 |
| Spain | 3.27 | 2.68 | 3.75 | 2.68 | 2.36 | 2.95 | 3.95 | 3.09 | 4.59 | 9.64 |
| Sweden | 7.73 | 7.67 | 7.79 | 5.34 | 5.83 | 4.88 | 9.82 | 9.29 | 10.29 | 3.47 |

Source: own work using EQLS(2016) and SHARE (waves 2, 4, 5, 6, 7 and 8)

**Table D4. Estimated coefficients for the life satisfaction model.**

|  | AT | BG | CZ | DK | FR | GE | IT | SE | SW |
| --- | --- | --- | --- | --- | --- | --- | --- | --- | --- |
| **Total sample** |  |  |  |  |  |  |  |  |  |
| Informal care | -0.921*** | -0.447*** | -0.617*** | -0.345*** | -0.488*** | -0.214*** | -0.647*** | -2.624*** | -0.283*** |
|  | (0.183) | (0.117) | (0.224) | (0.118) | (0.122) | (0.061) | (0.105) | (0.218) | (0.103) |
| Income (PPP) | 2.236*** | 1.496*** | 1.727*** | 2.134*** | 1.021*** | 0.755 | 1.755*** | 2.110*** | 2.051*** |
|  | (0.136) | (0.110) | (0.131) | (0.190) | (0.169) | (0.109) | (0.057) | (0.157) | (0.054) |
| N | 690 | 619 | 443 | 585 | 646 | 896 | 1.239 | 534 | 728 |
| R^2^ | 0.204 | 0.243 | 0.193 | 0.323 | 0.260 | 0.198 | 0.165 | 0.288 | 0.288 |
| F | 5.097 | 6.069 | 3.162 | 7.721 | 6.948 | 6.453 | 7.229 | 6.553 | 8.773 |
| p | 0.000 | 0.000 | 0.000 | 0.000 | 0.000 | 0.000 | 0.000 | 0.000 | 0.000 |
| **Men.** |  |  |  |  |  |  |  |  |  |
| Informal care | -0.467*** | -0.273*** | -0.506*** | -0.141*** | -0.189*** | -0.154*** | -0.244*** | -1.053*** | -0.153*** |
|  | (0.107) | (0.053) | (0.098) | (0.118) | (0.051) | (0.046) | (0.057) | (0.107) | (0.063) |
| Income (PPP) | 0.832*** | 0.787*** | 1.236*** | 1.132*** | 0.517*** | 0.428*** | 0.925*** | 1.222*** | 1.207*** |
|  | (0.160) | (0.138) | (0.227) | (0.130) | (0.078) | (0.137) | (0.176) | (0.201) | (0.085) |
| N | 461 | 323 | 238 | 301 | 396 | 448 | 616 | 281 | 299 |
| R^2^ | 0.257 | 0.343 | 0.302 | 0.380 | 0.290 | 0.242 | 0.253 | 0.352 | 0.383 |
| F | 5.135 | 5.710 | 3.360 | 4.955 | 5.347 | 4.295 | 6.169 | 4.522 | 5.995 |
| p | 0.000 | 0.000 | 0.000 | 0.000 | 0.000 | 0.000 | 0.000 | 0.000 | 0.000 |
| **Women** |  |  |  |  |  |  |  |  |  |
| Informal care | -0.454*** | -0.174*** | -0.111*** | -0.204*** | -0.299*** | -0.060*** | -0.403*** | -1.571*** | -0.131*** |
|  | (0.054) | (0.046) | (0.040) | (0.050) | (0.102) | (0.017) | (0.103) | (0.116) | (0.065) |
| Income (PPP) | 1.404*** | 0.709*** | 0.491*** | 1.002*** | 0.503*** | 0.327*** | 0.832*** | 0.888*** | 0.844*** |
|  | (0.259) | (0.185) | (0.172) | (0.127) | (0.143) | (0.075) | (0.088) | (0.253) | (0.069) |
| N | 229 | 296 | 205 | 284 | 250 | 448 | 623 | 253 | 429 |
| R^2^ | 0.261 | 0.215 | 0.184 | 0.367 | 0.374 | 0.202 | 0.125 | 0.352 | 0.291 |
| F | 2.516 | 2.716 | 2.548 | 5.504 | 4.906 | 3.398 | 3.017 | 4.529 | 5.652 |
| p | 0.000 | 0.000 | 0.000 | 0.000 | 0.000 | 0.000 | 0.000 | 0.000 | 0.000 |
| **Total. Less 50 years.** |  |  |  |  |  |  |  |  |  |
| cuidador | -0.463*** | -0.257*** | -0.091*** | -0.193*** | -0.173*** | -0.099*** | -0.331*** | -0.938*** | -0.141*** |
|  | (0.109) | (0.067) | (0.026) | (0.056) | (0.055) | (0.025) | (0.111) | (0.128) | (0.056) |
| lincome_2020 | 1.096*** | 0.964*** | 0.465*** | 1.208*** | 0.372*** | 0.249*** | 0.829*** | 1.413*** | 1.153*** |
|  | (0.188) | (0.134) | (0.046) | (0.111) | (0.024) | (0.039) | (0.095) | (0.239) | (0.068) |
| N | 262 | 319 | 201 | 304 | 286 | 394 | 514 | 244 | 399 |
| r2 | 0.266 | 0.299 | 0.319 | 0.406 | 0.334 | 0.241 | 0.237 | 0.454 | 0.365 |
| F | 2.788 | 4.091 | 2.879 | 6.975 | 4.421 | 3.841 | 4.989 | 6.662 | 6.562 |
| p | 0.000 | 0.000 | 0.000 | 0.000 | 0.000 | 0.000 | 0.000 | 0.000 | 0.000 |
| **Men. Less 50 years.** |  |  |  |  |  |  |  |  |  |
| Informal care | -0.331*** | -0.192*** | -0.046*** | -0.112*** | -0.051*** | -0.081*** | -0.119*** | -0.228*** | -0.076*** |
|  | (0.083) | (0.072) | (0.011) | (0.054) | (0.013) | (0.035) | (0.054) | (0.040) | (0.024) |
| Income (PPP) | 0.453*** | 0.508*** | 0.199*** | 0.918*** | 0.115*** | 0.088*** | 0.461*** | 0.639*** | 0.622*** |
|  | (0.141) | (0.061) | (0.046) | (0.167) | (0.045) | (0.024) | (0.143) | (0.138) | (0.028) |
| N | 158 | 150 | 91 | 149 | 164 | 170 | 217 | 122 | 149 |
| R^2^ | 0.379 | 0.464 | 0.303 | 0.513 | 0.342 | 0.214 | 0.363 | 0.575 | 0.537 |
| F | 2.944 | 4.293 | 1.344 | 4.946 | 2.743 | 1.650 | 3.981 | 5.198 | 4.963 |
| p | 0.000 | 0.000 | 0.177 | 0.000 | 0.000 | 0.038 | 0.000 | 0.000 | 0.000 |
| **Women. Less 50 years.** |  |  |  |  |  |  |  |  |  |
| Informal care | -0.132*** | -0.065*** | -0.044*** | -0.081*** | -0.122*** | -0.0183*** | -0.212*** | -0.711*** | -0.065*** |
|  | (0.048) | (0.027) | (0.010) | (0.021) | (0.041) | (0.002) | (0.086) | (0.061) | (0.026) |
| Income (PPP) | 0.643*** | 0.456*** | 0.266*** | 0.291*** | 0.257*** | 0.161*** | 0.368*** | 0.774*** | 0.531*** |
|  | (0.119) | (0.121) | (0.060) | (0.112) | (0.107) | (0.067) | (0.117) | (0.184) | (0.019) |
| N | 104 | 169 | 110 | 155 | 122 | 224 | 297 | 122 | 250 |
| R^2^ | 0.307 | 0.313 | 0.372 | 0.450 | 0.462 | 0.299 | 0.252 | 0.508 | 0.345 |
| F | 1.632 | 2.492 | 2.094 | 4.229 | 3.303 | 3.227 | 3.356 | 3.968 | 4.325 |
| p | 0.059 | 0.000 | 0.007 | 0.000 | 0.000 | 0.000 | 0.000 | 0.000 | 0.000 |
| **Total. 50+** |  |  |  |  |  |  |  |  |  |
| Informal care | -0.458*** | -0.191*** | -0.527*** | -0.152*** | -0.315*** | -0.115*** | -0.316*** | -1.686*** | -0.142*** |
|  | (0.046) | (0.033) | (0.124) | (0.063) | (0.060) | (0.042) | (0.032) | (0.287) | (0.062) |
| Income (PPP) | 1.141*** | 0.532*** | 1.262*** | 0.926*** | 0.648*** | 0.506*** | 0.926*** | 0.697*** | 0.898*** |
|  | (0.203) | (0.100) | (0.223) | (0.164) | (0.086) | (0.155) | (0.072) | (0.207) | (0.040) |
| N | 428 | 300 | 242 | 281 | 360 | 502 | 725 | 290 | 329 |
| R^2^ | 0.197 | 0.277 | 0.231 | 0.363 | 0.310 | 0.206 | 0.183 | 0.311 | 0.296 |
| F | 3.035 | 3.558 | 2.382 | 4.270 | 5.119 | 4.077 | 4.854 | 3.890 | 4.692 |
| p | 0.000 | 0.000 | 0.000 | 0.000 | 0.000 | 0.000 | 0.000 | 0.000 | 0.000 |
| **Men. 50+** |  |  |  |  |  |  |  |  |  |
| Informal care | -0.136*** | -0.081*** | -0.462*** | -0.029*** | -0.138*** | -0.073*** | -0.125*** | -0.825*** | -0.077*** |
|  | (0.013) | (0.021) | (0.100) | (0.025) | (0.023) | (0.028) | (0.018) | (0.191) | (0.027) |
| Income (PPP) | 0.379*** | 0.279*** | 1.037*** | 0.214*** | 0.402*** | 0.342*** | 0.464*** | 0.583*** | 0.585*** |
|  | (0.126) | (0.2024) | (0.289) | (0.073) | (0.079) | (0.118) | (0.096) | (0.164) | (0.023) |
| N | 303 | 173 | 147 | 152 | 232 | 278 | 399 | 159 | 150 |
| R^2^ | 0.229 | 0.400 | 0.402 | 0.421 | 0.337 | 0.331 | 0.251 | 0.306 | 0.396 |
| F | 2.901 | 3.585 | 3.255 | 2.931 | 3.833 | 4.391 | 4.253 | 2.140 | 3.248 |
| p | 0.000 | 0.000 | 0.000 | 0.000 | 0.000 | 0.000 | 0.000 | 0.002 | 0.000 |
| **Women. 50+** |  |  |  |  |  |  |  |  |  |
| Informal care | -0.322*** | -0.109*** | -0.067*** | -0.123*** | -0.177*** | -0.042*** | -0.191*** | -0.861*** | -0.065*** |
|  | (0.031) | (0.026) | (0.017) | (0.044) | (0.029) | (0.014) | (0.018) | (0.108) | (0.018) |
| Income (PPP) | 0.761*** | 0.253*** | 0.225*** | 0.712*** | 0.246*** | 0.166*** | 0.462*** | 0.114*** | 0.313*** |
|  | (0.132) | (0.051) | (0.049) | (0.179) | (0.106) | (0.042) | (0.115) | (0.038) | (0.013) |
| N | 125 | 127 | 95 | 129 | 128 | 224 | 326 | 131 | 179 |
| R^2^ | 0.313 | 0.237 | 0.273 | 0.439 | 0.516 | 0.181 | 0.149 | 0.507 | 0.339 |
| F | 4.633 | 4.252 | 5.231 | 6.229 | 4.574 | 5.475 | 5.934 | 4.915 | 3.289 |
| p | 0.000 | 0.000 | 0.000 | 0.000 | 0.000 | 0.000 | 0.000 | 0.002 | 0.000 |

Note: AT: Austria; BG: Belgium; CZ: Czech Republic; DK: Denmark; FR: France: GE: Germany; IT: Italy; SE: Spain; SW: Sweden.

Other explanatory variables: age, marital status, household size, size of municipality, relation with economic activity, current health status, having any permanent limitation and country fixed effects.Caregiving is instrumented using the following variables: number of daughters and number of sons.Current individual income (PPP2020) is instrumented using partner’s relation with economic activity and partner’s working hours.***denotes significance at the 99% level, **at the 95% level, *at the 90% level. Table D4 shows the estimated coefficients for informal care and income in the regressions for life satisfaction.

**Table D5. Compensating surplus, percentage of compensating surplus with respect to GDP, GDP per capita and annual wage.**

|  | Total | | | Age: younger than 50 years | | | Age: 50+ | | |
| --- | --- | --- | --- | --- | --- | --- | --- | --- | --- |
|  | Total | Men | Women | Total | Men | Women | Total | Men | Women |
| **Compensating surplus**  **(Euros PPP 2020)** |  |  |  |  |  |  |  |  |  |
| Austria | 13.372 | 16.992 | 10.950 | 13.670 | 19.616 | 7.600 | 13.082 | 12.680 | 13.294 |
| Belgium | 10.594 | 12.964 | 8.306 | 9.152 | 12.524 | 5.108 | 12.728 | 12.018 | 13.292 |
| Czechia | 11.246 | 10.680 | 8.332 | 6.706 | 7.652 | 5.890 | 12.598 | 10.242 | 11.228 |
| Denmark | 6.636 | 5.276 | 8.090 | 6.184 | 5.080 | 9.774 | 6.986 | 5.768 | 7.422 |
| France | 14.448 | 11.778 | 16.868 | 12.678 | 11.192 | 13.702 | 16.290 | 13.520 | 20.090 |
| Germany | 10.112 | 12.624 | 6.796 | 12.800 | 22.226 | 4.326 | 8.672 | 8.904 | 9.040 |
| Italy | 10.214 | 7.744 | 12.682 | 10.198 | 7.390 | 13.250 | 9.986 | 8.010 | 11.820 |
| Spain | 18.284 | 14.692 | 21.434 | 13.120 | 8.558 | 15.686 | 22.404 | 17.204 | 25.606 |
| Sweden | 5.820 | 5.588 | 6.216 | 5.146 | 5.092 | 5.200 | 6.638 | 5.972 | 8.026 |

Source: own work using EQLS (2016), Eurostat and ILOSTAT. Table D5 shows the compensating surplus (PPP €; 2020), the percentage of the compensating surplus (individual) to GDP per capita, the percentage of the compensating surplus (aggregated for all carers) to the GDP of the country. Finally, the percentage of the compensating surplus relative to the annual salary is also shown (in this case, the annual salary is different for men and women).

**Table D6. Comparison of compensating surplus with respect to GDP and value of informal care with respect to GDP (using the opportunity cost method)**

|  | Compensating surplus with respect to GDP (2016) | | | Estimation value informal care with respect to GDP (2016) |
| --- | --- | --- | --- | --- |
|  | All caregivers | Caregivers: younger than 50 years | Caregivers: 50+ |  |
| Austria | 2.12 | 1.11 | 1.02 | 1.90 |
| Belgium | 4.59 | 1.89 | 2.90 | 4.38 |
| Czechia | 3.87 | 1.28 | 1.93 | 2.05 |
| Denmark | 2.26 | 0.93 | 1.32 | 1.74 |
| France | 7.08 | 3.13 | 4.16 | 6.50 |
| Germany | 1.83 | 1.16 | 0.79 | 1.28 |
| Italy | 3.90 | 1.59 | 2.26 | 3.25 |
| Spain | 4.42 | 1.36 | 3.11 | 4.01 |
| Sweden | 1.98 | 0.80 | 1.18 | 1.20 |

Source: own work using EQLS (2016). Estimations of the value of informal care with respect to GDP are retrieved from Peña-Lonbgobardo et al. (2022). To value paid work time, they used the average gross hourly wage in purchasing power parity in each country, taking into account the caregiving hours provided by those caregivers who were employed. To value unpaid work time, they used the minimum gross hourly wage. Table D6 compares the ratio of CS to GDP with the value of informal care to GDP, (following estimates using the opportunity cost method carried out by Peña-Longobardo et al. (2022)). The ratio of CS to GDP for total carers is higher than the value of informal care in all countries, between 0.21pp (Belgium) and 1.82pp (Czech Republic), although in most countries it is half a percentage point higher. These results can be interpreted in two ways: (i) there is a relatively high degree of consistency between the two methods of valuing informal carers and (ii) the fact that (short-term) CS is higher seems to mean that there are feelings and costs (associated with carer burden) that are not captured by the opportunity cost method.

Reference:

Eurofund. 2017. *European Quality of Life Survey 2016: Quality of Life, Quality of Public Services, and Quality of Society*. Luxembourg: Publications Office of the European Union.
